# A fragment-based approach leading to the discovery of inhibitors of CK2α with a novel mechanism of action†

**DOI:** 10.1039/d2md00161f

**Published:** 2022-09-16

**Authors:** Paul Brear, Claudia De Fusco, Eleanor L. Atkinson, Jessica Iegre, Nicola J. Francis-Newton, Ashok R. Venkitaraman, Marko Hyvönen, David R. Spring

**Affiliations:** Department of Biochemistry, University of Cambridge Tennis Court Road CB2 1GA Cambridge UK mh256@cam.ac.uk; Yusuf Hamied Department of Chemistry, University of Cambridge Lensfield Road CB2 1EW Cambridge UK spring@ch.cam.ac.uk; Medical Research Council Cancer Unit, University of Cambridge, Hutchison/MRC Research Centre Cambridge CB2 0XZ UK; Cancer Science Institute of Singapore, National University of Singapore 14 Medical Drive, Singapore 117599 & DITL, IMCB, A*STAR, 8A Biomedical Grove 138648 Singapore

## Abstract

CK2 is a ubiquitous protein kinase with an anti-apoptotic role and is found to be overexpressed in multiple cancer types. To this end, the inhibition of CK2 is of great interest with regard to the development of novel anti-cancer therapeutics. ATP-site inhibition of CK2 is possible; however, this typically results in poor selectivity due to the highly conserved nature of the catalytic site amongst kinases. An alternative methodology for the modulation of CK2 activity is through allosteric inhibition. The recently identified αD site represents a promising binding site for allosteric inhibition of CK2α. The work presented herein describes the development of a series of CK2α allosteric inhibitors through iterative cycles of X-ray crystallography and enzymatic assays, in addition to both fragment growing and fragment merging design strategies. The lead fragment developed, fragment 8, exhibits a high ligand efficiency, displays no drop off in activity between enzymatic and cellular assays, and successfully engages CK2α in cells. Furthermore, X-ray crystallographic analysis provided indications towards a novel mechanism of allosteric inhibition through αD site binding. Fragments described in this paper therefore represent promising starting points for the development of highly selective allosteric CK2 inhibitors.

## Abstract 2

Development of a novel CK2α inhibitor from a fragment-based screen with a proposed novel mechanism of action.

## Graphical abstract

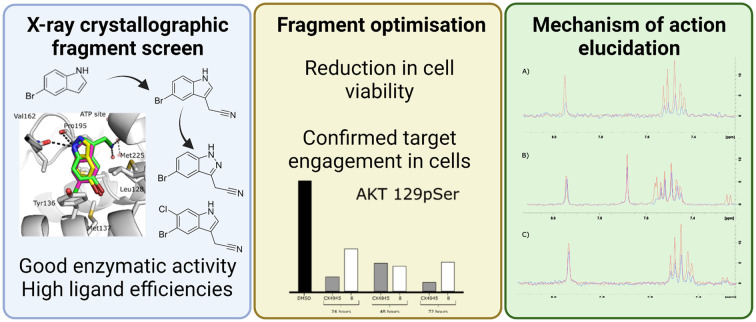

## Introduction

CK2 is a pleiotropic, constitutively active Ser/Thr kinase with one of the largest sets of target proteins of eukaryotic protein kinases.^1^ CK2α is found both as an isolated active CK2α subunit and as a larger complex, with a scaffolding β subunit, and is active in both forms. The role of CK2α is to recruit substrates and to control the intracellular localisation of the kinase.^2^ CK2 has been found to be overexpressed in numerous cancer cells, and many of its roles in cancer, such as modulation of cell proliferation and cell death, are attributable to its activity in the nucleus.^3^ There are no known mutations in CK2α implicated in cancer, in line with its constitutively active role, and rather than an oncoprotein, it is considered a facilitator that enhances tumorigenesis.^4^

One of the key roles of CK2α is to promote anti-apoptotic pathways through activation of the NF-κB and Wnt signalling pathways.^5^ As a pro-survival kinase, overexpression of CK2α appears to both facilitate the growth of tumour cells and provide a mechanism for the cells to avoid apoptosis. Therefore, cells rely on CK2α's activity to survive, becoming addicted to its non-oncogenic activity which is typically enhanced by increasing CK2α expression. This is particularly true for cells that are challenged with chemotherapeutic agents and the most promising uses for CK2α inhibitors stem from these observations.^6^

Drug resistance is a serious problem for cancer therapies, and agents that can reverse or prevent the emergence of drug resistance are desperately needed. By suppressing survival signals, CK2α inhibition shows great potential as a combination therapy agent that could be used with tumour-specific agents to both augment their efficacy and minimise the development of resistance, or even reverse it.^7–9^ Indeed, inhibition of CK2α has been demonstrated to help overcome resistance to specific chemotherapeutic agents. For example, imatinib-resistant CML cells were shown to become sensitive to the drug again when co-administered with CK2α inhibitors.^10–13^

There are also examples of tumours where CK2α could be effective as a monotherapy target. For example, glioblastomas, irrespective of their stage or p53 status, have been shown to be particularly vulnerable to CK2α inhibition. Glioblastomas are traditionally hard-to-treat cancers, highly malignant and with poor prognosis and thus novel treatments for these tumours is of great importance. On the contrary, non-cancerous glial cells appear to be unaffected by CK2α inhibition suggesting that selectivity for cancer cell death could be achieved with CK2-inhibiting therapies.^14^

ATP competitive inhibitors of CK2α have been extensively studied, but they tend to suffer from relatively poor kinase specificity, as exemplified by the clinical candidate CX-4945.^15–17^ Despite its limited selectivity, CX-4945 is well-tolerated, suggesting that inhibition of CK2α activity is a viable therapeutic target, notwithstanding the ubiquitous roles of CK2 in cells.^18^ However, promiscuous inhibitors are likely to have more serious side effects if used therapeutically and thus it is desirable to have more selective kinase inhibitors to probe the true potential of CK2α inhibition in the clinic.

A promising strategy for the development of more selective CK2α inhibitors is to target binding pockets outside of the conserved ATP binding site. Previously, we have used a fragment-based approach to discover a new, cryptic pocket located next to the ATP site, termed the αD site.^6,19,20^ This pocket provides an exciting opportunity for the development of more specific CK2α inhibitors with novel mechanisms of action. We initially utilised this new pocket to develop a proof-of-concept molecule by growing a fragment hit from the αD pocket into the ATP site by linking it to a low-affinity active site “warhead”. The resulting molecule (CAM4066) is a nanomolar CK2α inhibitor with better selectivity than any other previously described CK2α inhibitor and inhibits the growth of several cancer cell lines.^6,19^

In this work, using a fragment-based approach, we discovered novel molecules that could inhibit the activity of CK2α through binding in the αD pocket. The work described herein showcases how CK2 inhibition can be achieved without relying on interactions with the highly conserved ATP binding site and, therefore, paves the way towards the development of highly selective CK2α inhibitors.

## Results and discussion

**Fig. 1:**
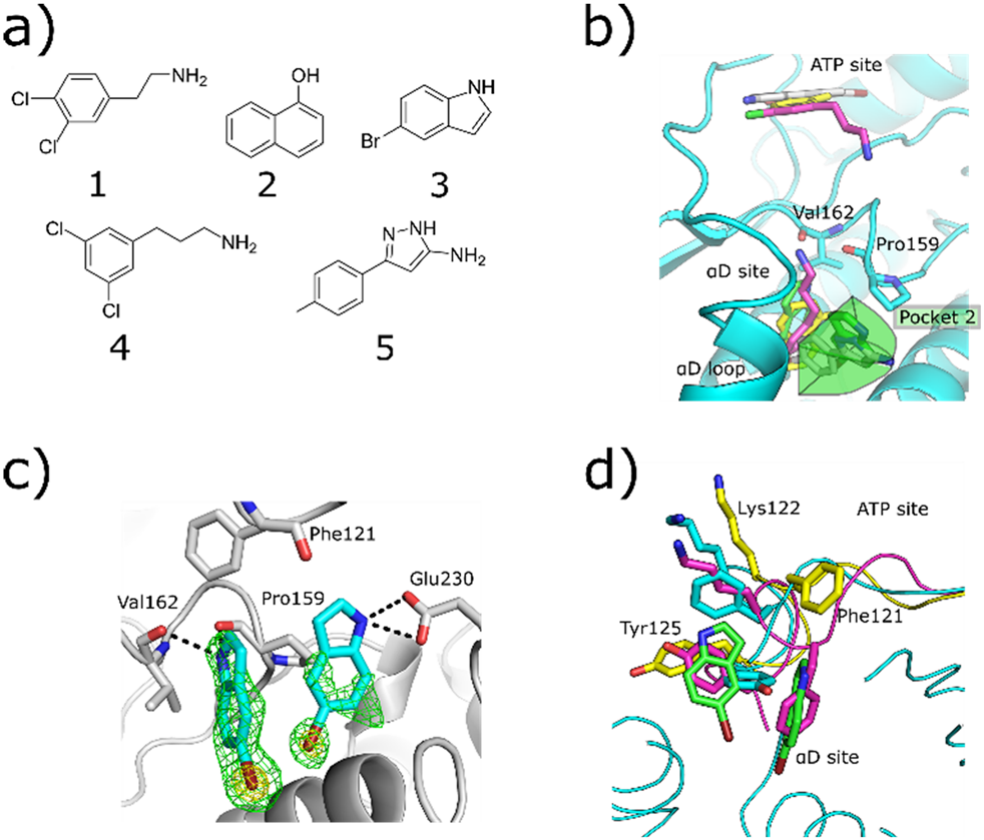
a) The structures of the five fragments found in the αD site from the fragment screen. b) The binding poses of the five αD-site fragments. 3 and 5 both bind in the main αD site and in pocket 2, which is highlighted in green. This pocket is created by displacement of Tyr125 (PDB: 7ZY2, 7ZY5, 7ZT8, 5CLP). c) The *F*_o_–*F*_c_ density maps are shown in green, and the anomalous difference map contoured at 10*σ* is shown in yellow. The hydrogen bonding interactions between 3 and the αD pocket are also highlighted with grey dotted lines (PDB: 7ZY2). d) The movement of the αD loop that allows the binding of 3 in the αD pocket. The closed conformation is shown in purple where Phe121 occupies the main αD pocket and Tyr125 fully occupies pocket 2 of the αD site. The partially open conformation is shown in light blue. In this conformation Tyr125 partially fills the αD pocket. The open conformation is shown in yellow. In this conformation, Phe121 blocks the top of the αD site and stacks with 3, forming the side of pocket 2 of the αD site.

The original fragment screens that led to the discovery of the αD pocket identified five fragments which bound in the αD pocket (Fig. 1a).^6^ The elaboration of fragment 1 into the inhibitor CAM4066 has been reported previously.^6,19^

X-ray crystallography showed that fragments 2, 4 and 5 bound in both the αD site and ATP site and so were of limited use for probing the αD site (Fig. 1b). Fragment 3, however, was observed to bind solely in the αD pocket and thus was selected for further optimisation. Fragment 3 is a relatively small molecule (196 Da) and thus a weak binder. Therefore, it was screened at high concentration (1 mM) in a kinase activity assay against CK2α to probe its activity. Fragment 3 showed weak inhibition of CK2α phosphorylation (50 ± 13% at 1 mM), and hence compound optimisation was undertaken.

**Fig. 2:**
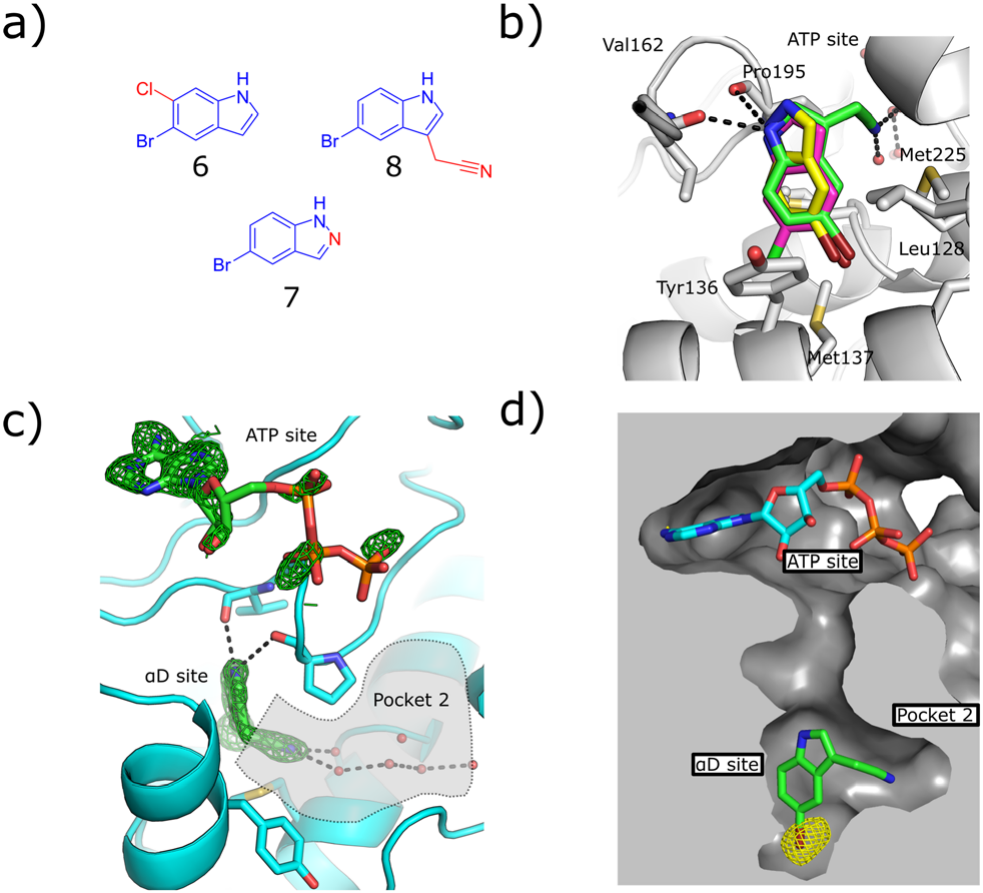
a) The structures of the three hit compounds from the screen of commercially available analogues of 3. The core structure of 3 is shown in blue and the extra sections are shown in red. b) An overlay of the crystal structures of the three fragments found bound in the virtual secondary screen. The conserved interactions with the backbone carbonyl of Pro195 and the new interactions formed by the nitrile binding in pocket 2 are shown (PDB: 7ZY2, 7ZY0, 7ZYD). c) The *F*_o_–*F*_c_ density map of fragment 8 is shown in green. The adenosine ring of ATP is clearly seen; however, the electron density for the triphosphate group is poorly defined. Pocket 2 is highlighted in grey, the interactions between the nitrile group and the conserved waters in pocket 2 are shown (PDB: 7ZY0). d) The anomalous diffraction map for 8, bound in the αD pocket, contoured at 5*σ*, and covering 8 and ATP is depicted in yellow. The map clearly shows the presence of the bromine in the αD site but not the ATP site, confirming that no bromine is bound in the ATP site to account for the inhibition. The surface of the pocket formed by the binding of 8 is shown (PDB: 7ZY0).

To this end, a virtual secondary screen was conducted whereby a series of commercially available and in-house analogues of 3 were docked using glide and the promising compounds from this virtual screen were tested against CK2α. This was performed initially as a crystallographic screen, after which any compounds that bound solely in the αD site were followed up by the kinase activity assay. From this process, three more fragments were discovered to bind only in the αD pocket (6, 7 and 8, Fig. 2a) and were therefore regarded as of great interest.

Fragment 8 bound in the primary αD pocket in a similar manner to 3, with the nitrile arm of 8 pointing into pocket 2. The nitrile formed two hydrogen bonding interactions with the water molecules in the pocket that bridge to Glu230 and Ser224 at the back of pocket 2 (Fig. 2d).

Reasonable density was observed for ATP in the ATP site, suggesting that the inhibition observed was due to binding in the αD site rather than displacement of ATP. This is supported also by our previous observations that even very weak fragments can displace ATP in these structures.^6^ A very good anomalous signal for bromine confirmed that fragment 8 bound only in the αD site and not in the ATP site. 8 showed disordered density in the ATP site that could not be properly assigned to ATP or 8, however, no anomalous signal was observed in the ATP site for 8, meanwhile a good anomalous signal was observed in the αD site. Fragments 6, 7 and 8 exhibited similar binding poses to 3 and maintained the key interactions observed previously.

The kinase assay revealed that 6 and 7 still caused only weak inhibition of CK2α (IC_50_ = 333 ± 24 μM and 410 ± 26 μM) whilst also exhibiting promising ligand efficiencies of 0.44 and 0.47, respectively (Table 1). Promisingly, 8 showed, for a fragment, good inhibition of CK2α (IC_50_ = 86 ± 24 μM) which again led to a promising ligand efficiency of 0.44 (Table 1).

**Table 1:** Structures of 3 and its three derivatives, 6, 7 and 8, alongside their PDB codes, % inhibitions, IC_50_ values, ligand efficiencies and GI_50_ values. IC_50_ values are given as the mean ± SEM from three repeats; GI_50_ values are given as the mean ± SEM from three experiments run in triplicate; N.S. = not soluble; N/A = not applicable; *under deposition

Compound	Structure	PDB	% inhibition at 1 mM	% inhibition at 500 μM	IC_50_ (μM)	Ligand efficiency (kJ mol^−1^)	GI_50_ HCT116 (μM)	GI_50_ Jurkat (μM)	GI_50_ A459 (μM)
3	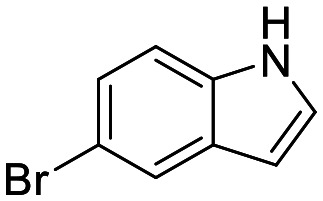	7ZY2	50 ± 13	22 ± 5	N/A	N/A	N/A	N/A	N/A
6	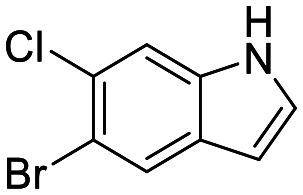	7ZYD	N.S.	93 ± 5	333 ± 24	0.44	N/A	N/A	N/A
7	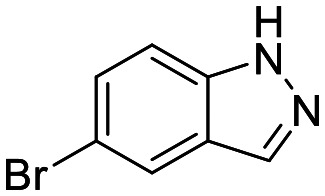	*	84 ± 6	66 ± 5	410 ± 26	0.47	N/A	N/A	N/A
8	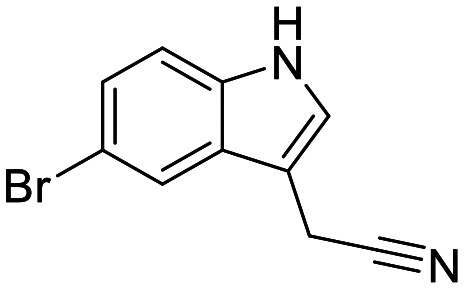	7ZY0	105 ± 9	87 ± 11	86 ± 24	0.44	88 ± 7	55 ± 2	29 ± 7

### Further fragment elaboration

**Fig. 3:**
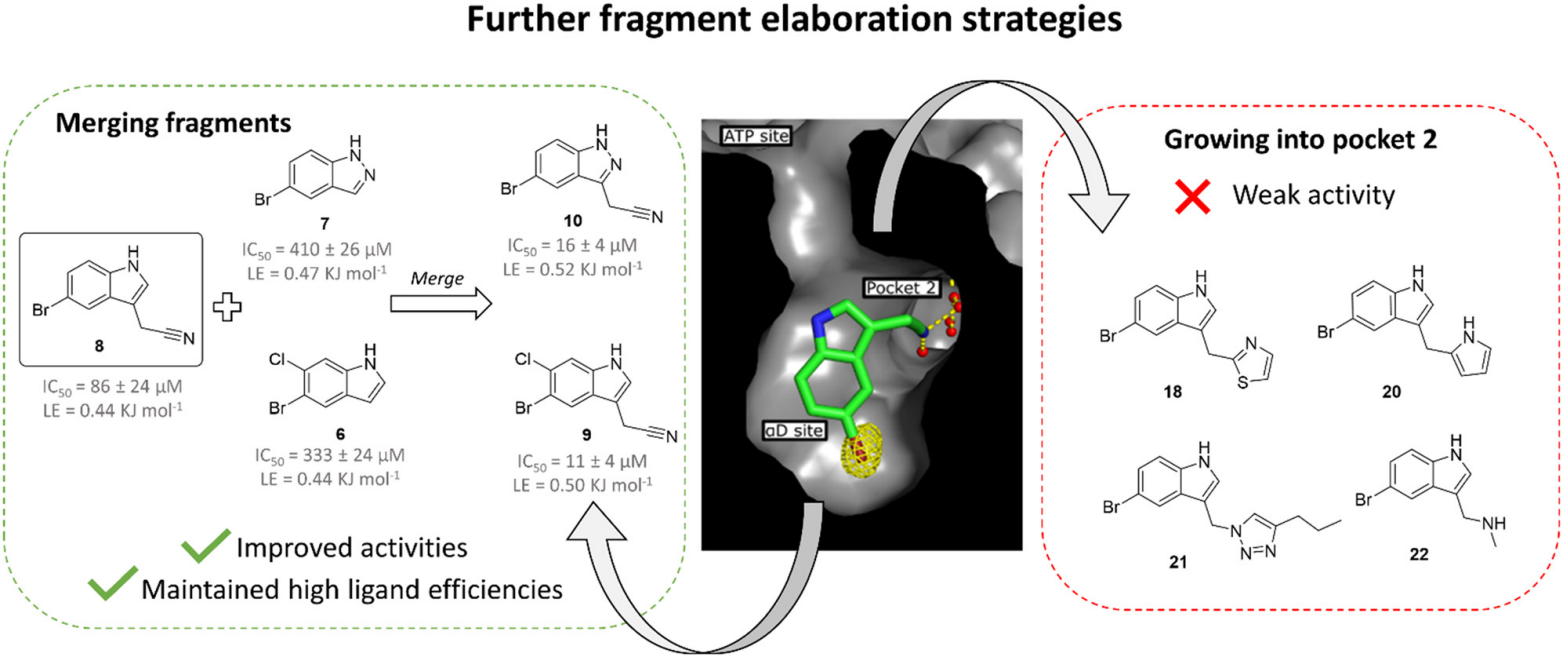
Overview of the two fragment elaboration strategies attempted: growth into pocket 2 and fragment merging. Compounds 18, 20, 21 and 22 derive from proposed growth of fragment 8 into pocket 2 but this series of compounds exhibited no activity improvement relative to fragment 8 (ESI† Table S2) and, thus, this strategy was not explored further. Fragments 9 and 10 were designed based on the merging of fragment 8 with 6 and 7, respectively. This strategy led to the identification of compounds with improved activities compared to 8 and high ligand efficiencies.

As fragment 8 showed both good ligand efficiency and kinase inhibition, further optimisation was focused on this compound for the development of a higher affinity inhibitor. This study employed two separate strategies (Fig. 3). Firstly, the effect of elaborating 8 from the 3 position of the indole, where it could grow into pocket 2, was investigated.

Secondly, fragment 8 was merged with the other two fragments identified in the virtual secondary screen. This was combined with the screening of analogues of 8 with different substitutions on the indole core.

### Growth into pocket 2

The pyrazole ring of 5 and the Tyr125 of the αD loop were observed to occupy pocket 2 of the αD site (Fig. 1b and d). The alignment of fragments 8 and 5 with Tyr125 suggested that fragment 8 could be grown into pocket 2. However, testing of a number of such compounds in crystal structures and in the inhibition assay did not lead to an increase in inhibition (ESI† Table S2) and so this strategy was not pursued further (Fig. 3).

### Fragment merging

Both fragments 6 and 7 bound in the αD pocket and crystal structures suggested that they could be merged. Initially, fragment 8 was separately merged with fragments 6 and 7 to give compounds 9 and 10, respectively (Fig. 3). The merged compounds exhibited moderately improved IC_50_ values of 11 ± 4 μM and 16 ± 4 μM (9 and 10, respectively) and maintained high ligand efficiencies (≥0.50). The bromine of fragment 8 was also systematically varied with a range of common substituents (H, F, Cl, CF_3_, compounds 11 to 14, ESI† Table S1). However, only mutation to CF_3_ maintained the inhibition seen with the bromo substituent. Nevertheless, this substitution led to a significant decrease in ligand efficiency. For these reasons, this strategy was not pursued further.

X-ray crystallography showed that compounds 11 to 14 all maintained the binding mode of 8. The anomalous density maps of 9 and 10 showed that bromine was only bound in the αD site, with ATP clearly bound in the active site (ESI† Fig. S2).

A series of analogues substituted in the 6 position of the indole were subsequently tested (ESI† Table S3). From these, only 15 led to a moderate improvement of inhibition of CK2α, with an IC_50_ value of 19 ± 15 μM. However, although all other structures thus far showed clear density for ATP, the structure with 15 showed weak density for the fragment in the ATP site as well as the αD site.

Thus, it is proposed that this series may partially bind in in the ATP site. Furthermore, the substituents in 15 significantly increased the lipophilicity of the compound, leading to solubility issues. Therefore, it was hypothesised that this series would not lead to a robust chemical tool.

A final attempt to increase the solubility of the series was made by merging fragment 10 with 9 and 15 to give compounds 16 and 17, respectively. Both 16 and 17 gave similar inhibition of CK2α (5 ± 1 μM and 6 ± 1 μM, respectively) as determined by the phosphorylation assay and similar binding affinities as determined by ITC (*K*_d_ = 10.2 μM and 15.8 μM, ESI† Fig. S3), but failed to show increased solubility.

### Cellular assays

Although compounds 9, 15, 16 and 17 were not soluble enough to be tested against the HCT116 cell line, fragment 8 did not present solubility issues and thus it could be investigated further in cellular assays.

**Fig. 4:**
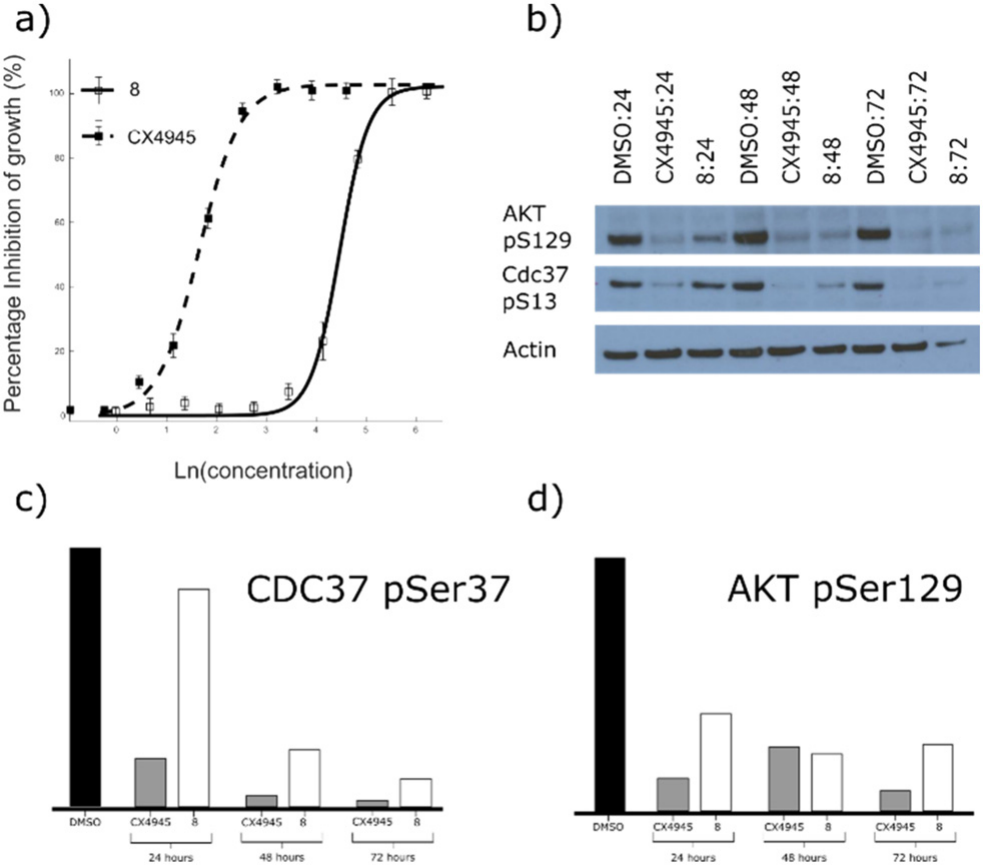
a) Dose response curve for the inhibition of the growth of HCT116 cells by 8 and CX4945. CX4945 has a GI_50_ of 4.8 μM ± 3 and fragment 8 has a GI_50_ of 88 ± 7 μM (graph shows the mean ± SEM of three experiments with each concentration in triplicate). b) Western blot analysis showing the specific CK2 phosphorylation targets of AKT1 phosphoserine 129 and CDC37 phosphoserine 13. HCT116 cells were treated with 2 x GI_50_ of CX4945 (10 μM) or 8 (170 μM) for 24, 48 or 72 hours. c) and d) quantitative results of the Western blot shown in (b) for AKT1 phosphoserine 129 and CDC37 phosphoserine 13, respectively. Data are normalised to actin loading control and to DMSO-only sample.

As fragment 8 demonstrated high inhibition, for a fragment, in addition to a novel mode of action, its ability to inhibit the growth of three different cell lines was investigated. In these assays, 8 showed good growth inhibition in HCT116, Jurkat and A549 cells, with GI_50_ values of 88 ± 7, 55 ± 2 and 29 ± 7 μM, respectively (Fig. 4a, ESI† Fig. S4). Unlike most other CK2α inhibitors, there was no drop off in activity between the kinase assay and the proliferation assay. This indicates that fragment 8 has very good cell permeability.

The target engagement of 8 was proven by following CK2α-dependent phosphorylation of Ser129 in AKT1 and Ser13 in CDC37. Fragment 8 showed good inhibition of the CK2α-dependent phosphorylation of Ser129 in AKT1 at twice the GI_50_ for all three time points investigated (Fig. 4b and d). Phosphorylation of Ser13 in CDC37 was significantly inhibited 48 h after incubation with 8 at twice the GI_50_ (Fig. 4c).

### NMR competition study

**Fig. 5:**
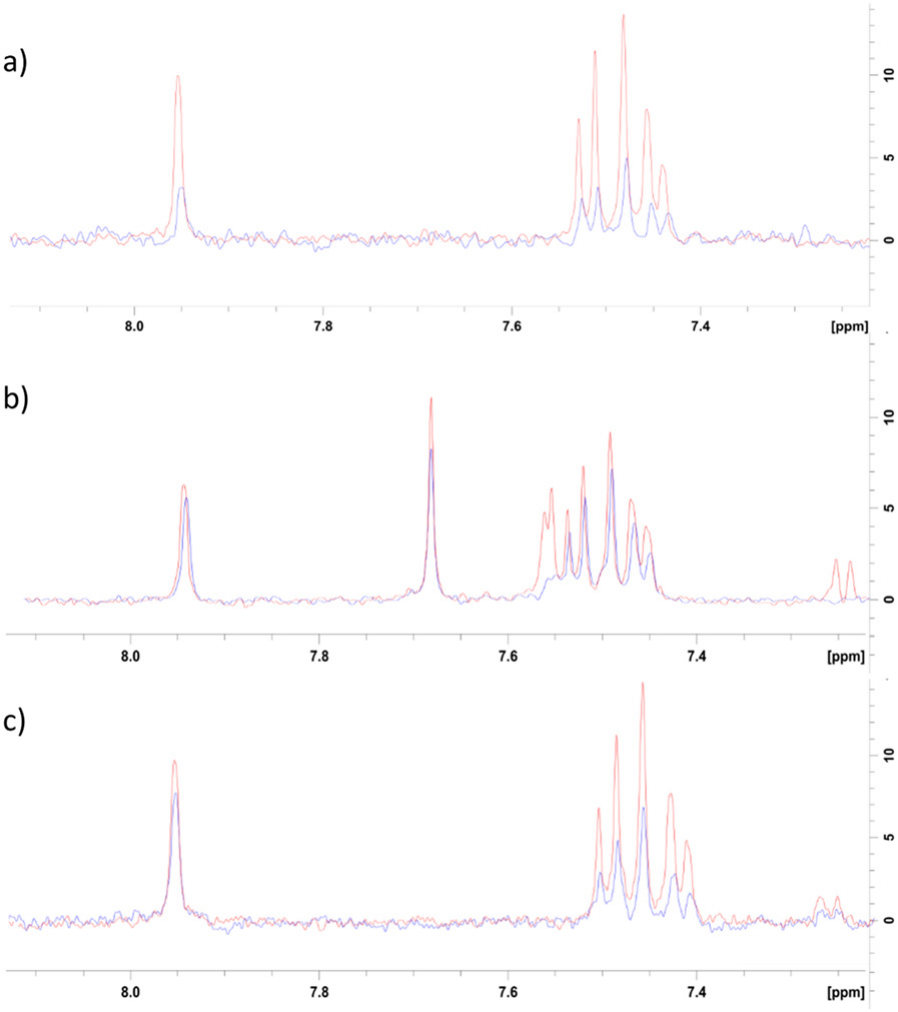
NMR competition study with known CK2 inhibitors. a) Red = 500 μM 8 + 0 μM CK2α, blue = 500 μM 8 + 7 μM CK2α. b) Red = 500 μM 8 + 500 μM αD-binder^20^ + 0 μM CK2α, blue = 500 μM 8 + 500 μM αD-binder + 7 μM CK2α. c) Red = 500 μM 8 + 50 μM CX4945 + 0 μM CK2α, Blue = 500 μM 8 + 50 μM CX4945 + 7 μM CK2α.

To further investigate the method by which 8 was inhibiting CK2α, an NMR competition study was performed whereby well-validated CK2α ligands were used to further characterise the binding mode of 8. Firstly, the binding of 8 to CK2α was evaluated by a ligand-observed CPMG experiment. In the presence of CK2α, the signal for 8 was reduced, confirming it binds to CK2α (Fig. 5a).

Two well-validated ligands that bind to specific sites of CK2α were then used in a competition study to identify the binding site for 8. These ligands were a previously reported CK2 αD site binder (compound 15 in the paper, herein referred to as αD-binder)^20^ and CX4945, which binds in the ATP site of CK2α.^21^ The CPMG experiment was performed at 500 μM of αD-binder and 500 μM of 8 (Fig. 5b and c).

As expected, the binding of fragment 8 was largely inhibited by the αD-binder, indicating that fragment 8 does indeed bind in the αD site (Fig. 5b).

The result from the experiment with CX4945 was less clear. In the presence of CX4945, the binding of fragment 8 was significantly reduced (Fig. 5c). This could be for a few reasons. Firstly, the difference could be due to some binding of 8 in the ATP site that was not observed in the crystal structures. Secondly, the reduction in binding could be because binding in the ATP site affects binding in the αD site and *vice versa*, which is likely if 8 and its analogues affect CK2α through an allosteric mechanism. Further study would be needed to explain this observation.

### Mechanism of inhibition

The crystal structures of all the key fragments studied herein clearly showed that these compounds bound to CK2α in the αD pocket and appear to inhibit the enzyme through allostery. The crystal structures yield several clues as to the possible mechanism of the allosteric inhibition.

#### 1) ATP binding modulation

Firstly, ATP binding may be altered by changes in the hinge region caused by the movement of the αD loop. Specifically, the movement of the hinge region causes Asn118 to rotate out from the mouth of the αD pocket into a position where it participates in a water bridge to ATP. Asn118 has previously been shown by Srinivasan *et al.* to be a crucial residue: when Asn118 was deleted, CK2α was no longer active.^22^ Asn118 is replaced upon its movement by a conserved water which sits at the mouth of the αD site where it interacts with the backbone carbonyls of Thr119 and Ile164.

#### 2) Preventing the transition between open and closed form

A second possible mechanism of inhibition could be the blocking of the transition between the open and closed form. As has been discussed previously, the αD loop is uniquely flexible in CK2α and has been observed in multiple conformations, most commonly the open and closed forms in crystallography.^6,23^ When ATP or its analogues are bound, the loop normally adopts an open conformation, but when the ATP site is empty, or occupied by a compound not interacting with the hinge region in the same way as ATP, the loop adopts a closed conformation. From this it can be inferred that when ATP leaves the ATP site, the loop will then adopt the closed conformation; the transition to the closed conformation may be important for causing ADP to leave the ATP site. However, when the αD site is occupied by the fragments presented herein, this transition cannot occur, possibly stalling the phosphorylation mechanism.

Many of the previously reported αD-site fragments did not display CK2α inhibition.^19^ This could be attributed to a number of different factors. Firstly, many of the previous fragments bound very weakly so inhibition would not be observable in the assay. Secondly, in the structures of many of these previous fragments (*e.g.* PDB entry 5CS6) the αD loop is displaced and enters a more disordered open conformation. On the contrary, when the allosteric fragments bind, the αD loop is locked in a more rigid, ordered conformation which is also reflected in lower B-factors for this loop (ESI† Fig. S5). The extra mobility of the αD loop may allow the mechanism of phosphorylation to progress for inactive fragments, whereas the rigid conformation adopted when these inhibitors bind to the αD pocket may block the enzyme activity.

## Conclusions

We have presented five diverse fragments bound in the αD site. One of these fragments (3) bound solely in the αD site and showed weak inhibition of the kinase activity of CK2α. This weakly binding fragment was optimised into a higher affinity molecule, fragment 8, that bound solely in the αD site and showed clear inhibition of CK2α (IC_50_ = 86 ± 24 μM). Furthermore, fragment 8 also showed promising inhibition of cell growth through inhibition of phosphorylation by CK2α (GI_50_ = 88 ± 7 μM). This fragment-sized tool molecule, alongside its derivatives, confirmed that the αD site can be successfully exploited to achieve inhibition of CK2. Although 8 is unlikely to display high kinase selectivity due to its small size, it opens the potential for further development to identify a selective tool compound for CK2. The binding site was confirmed in solution by ligand-based NMR and the crystal structures of the various fragments bound gave several indications as to the mechanism of allosteric inhibition, but more detailed analyses are needed to eliminate any orthosteric effect on the ATP site by 8. These molecules and the information we have presented herein represent a promising starting point for future development and structure-based design of allosteric CK2α inhibitors which operate by a novel mechanism of action.

## Conflicts of interest

There are no conflicts to declare.

## Supplementary Material

MD-013-D2MD00161F-s001
