# Supplementary material for: A fragment-based approach leading to the discovery of inhibitors of CK2α with a novel mechanism of action
Source: RSC Med Chem. 2022 Sep 16;13(11):1420–6. doi: 10.1039/d2md00161f (PMC9667777; doi:10.1039/d2md00161f)
Supplement: MD-013-D2MD00161F-s001 [file MD-013-D2MD00161F-s001.pdf]

Electronic Supplementary Material (ESI) for RSC Medicinal Chemistry.
This journal is © The Royal Society of Chemistry 2022

Supporting information

A fragment-based approach leading to the discovery of inhibitors of CK2α with a novel mechanism of action

Paul Brear, Claudia De Fusco, Eleanor L. Atkinson, Jessica Iegre, Nicola J. Francis-Newton, Ashok R. Venkotaraman, Marko Hyvönen and David R. Spring

Table of Contents

1. Figures, Tables and Schemes 3

2. Chemistry experimental 11

2.1 Experimental synthetic details 12

2.2 NMR spectra 27

3. Biophysical experiments 50

3.1 Protein expression and purification 50

3.2 Isothermal titration calorimetry 50

3.3 X-ray crystallography 50

3.4 NMR competition experiment 54

4. Cellular biology 54

4.1 Cell culture 54

4.2 Cell viability assay 54

4.3 Western Blotting 55

5. Modelling 56

6. References 57

1. Figures, Tables and Schemes

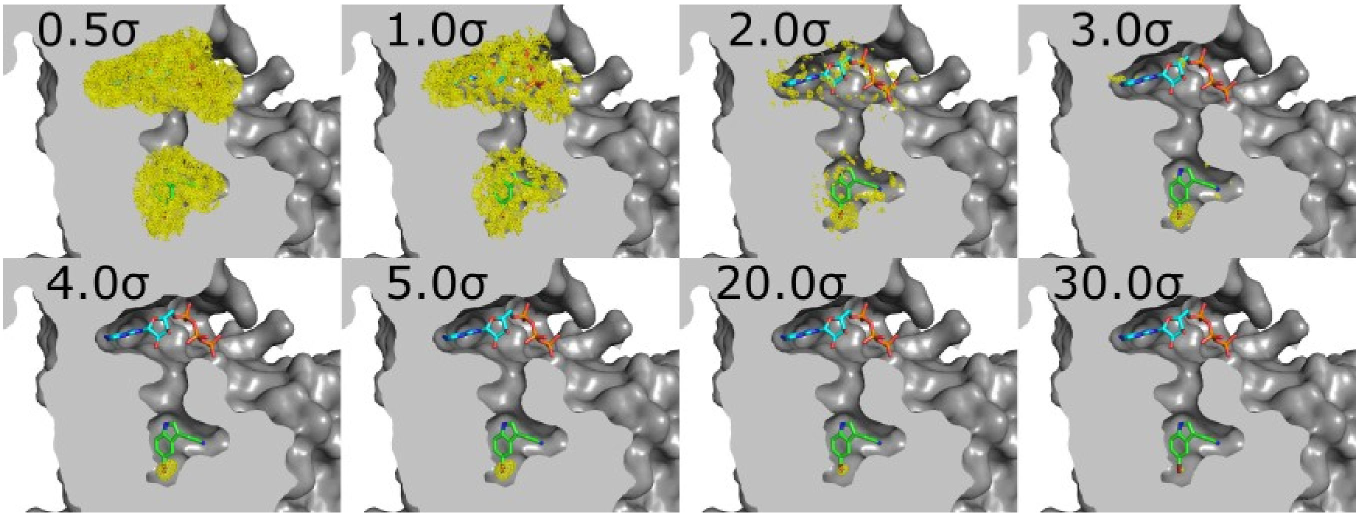

ESI Figure S1 The anomalous diffraction map for **8** bound in the αD pocket, contoured at 5σ to 30σ, and covering **8** and ATP is depicted in yellow (PDB:7ZY0). The map clearly shows the presence of the bromine in the αD site but not the ATP site, confirming that no bromine is bound in the ATP site to account for the inhibition. The surface of the pocket formed by the binding of **8** is shown.

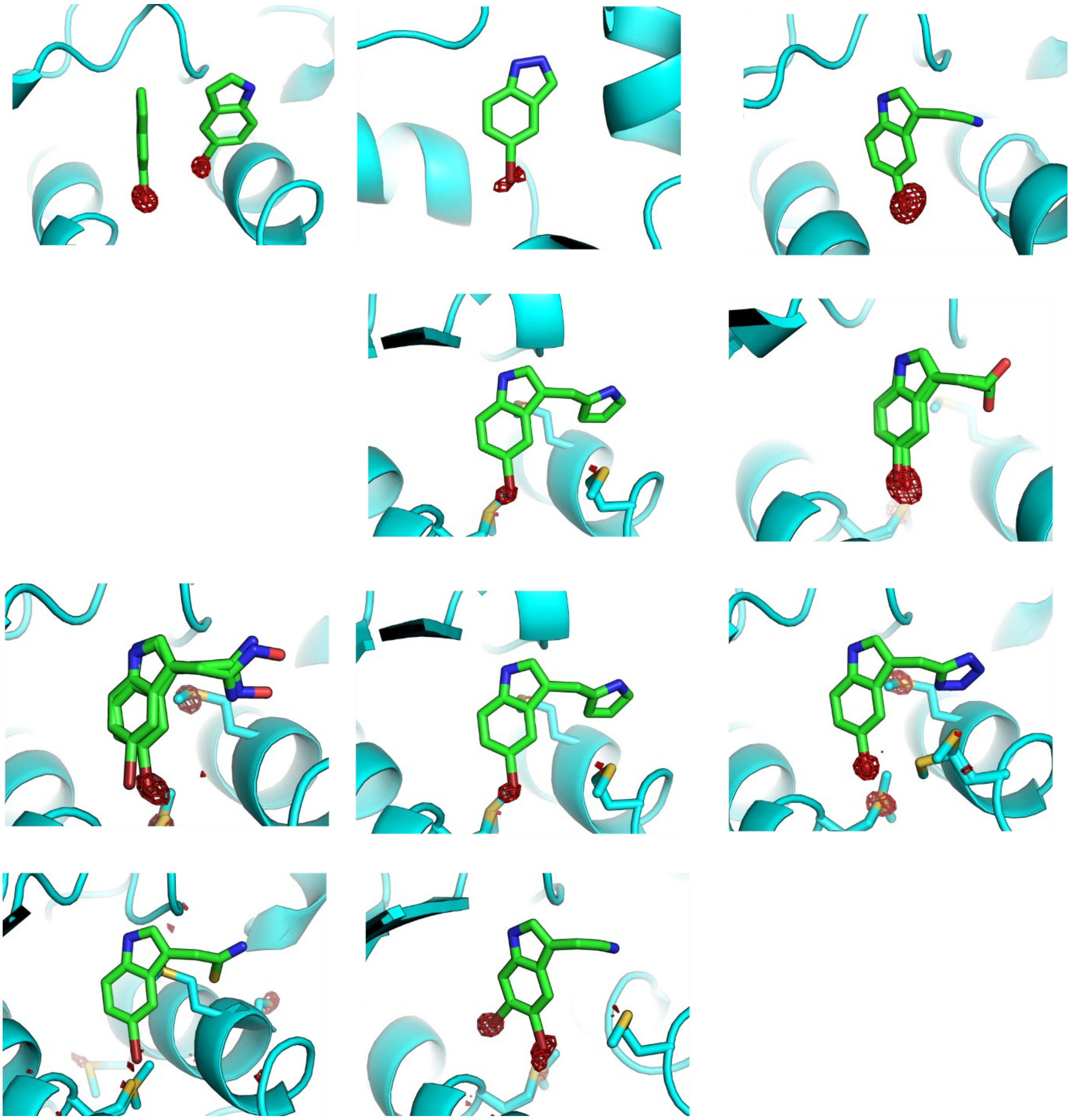

ESI Figure S2 Anomalous diffraction for data collected at the Br edge, λ = 0.9184, for various compounds

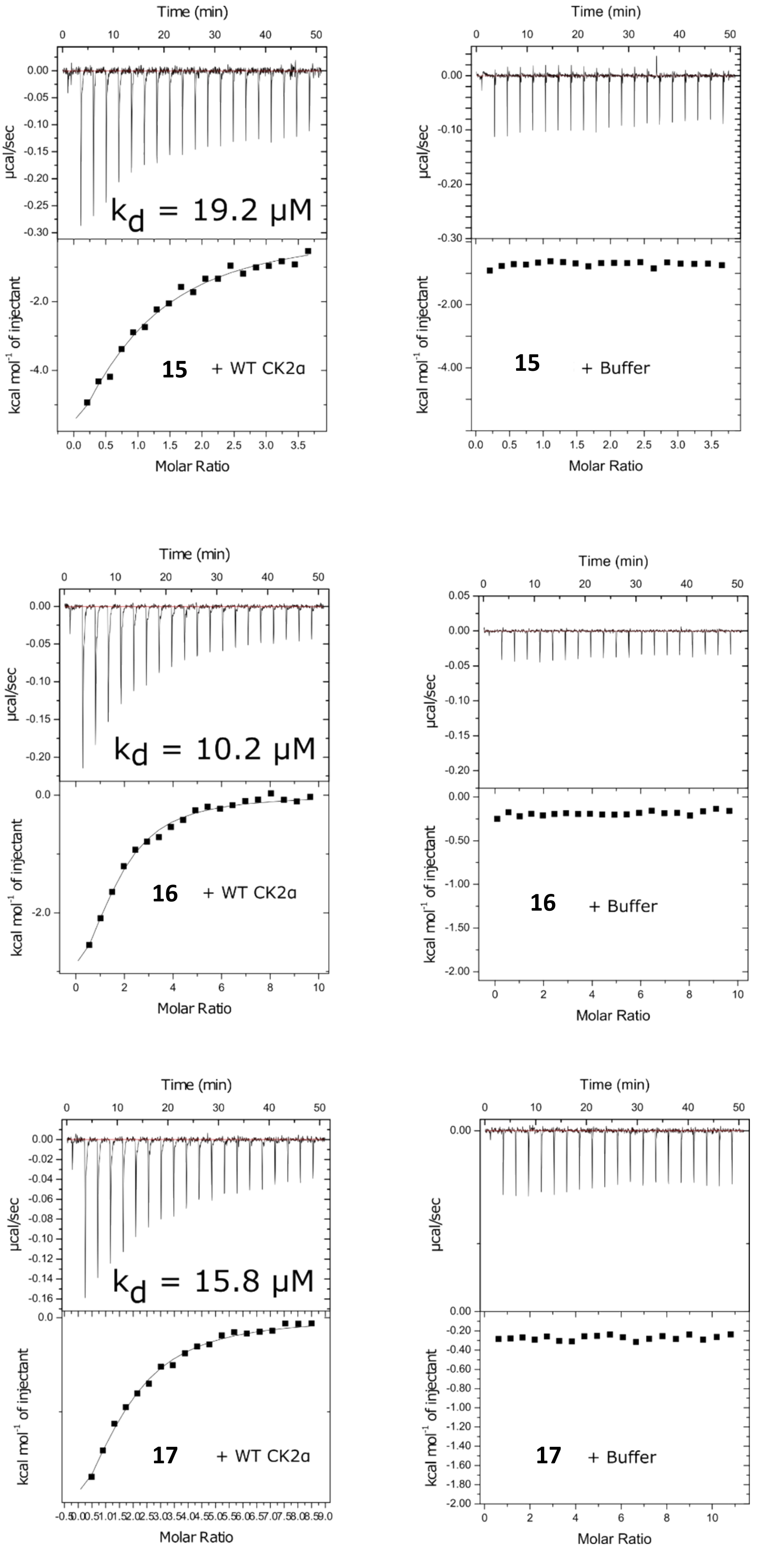

ESI Figure S3 Binding affinities of optimised allosteric compounds **15**, **16** and **17** for CK2α, as determined by ITC.

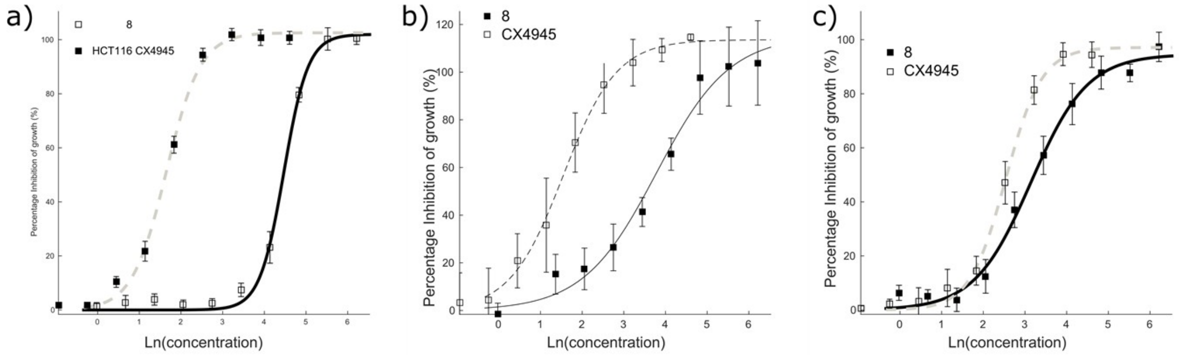

ESI Figure S4 Dose response curves for the inhibition of the growth of a) HCT116, b) Jurkat and c) A459 cells by **8** and CX4945. GI_{50} values are shown in Table 1. Graph shows the mean ± SEM of three experiments, with each concentration in triplicate.

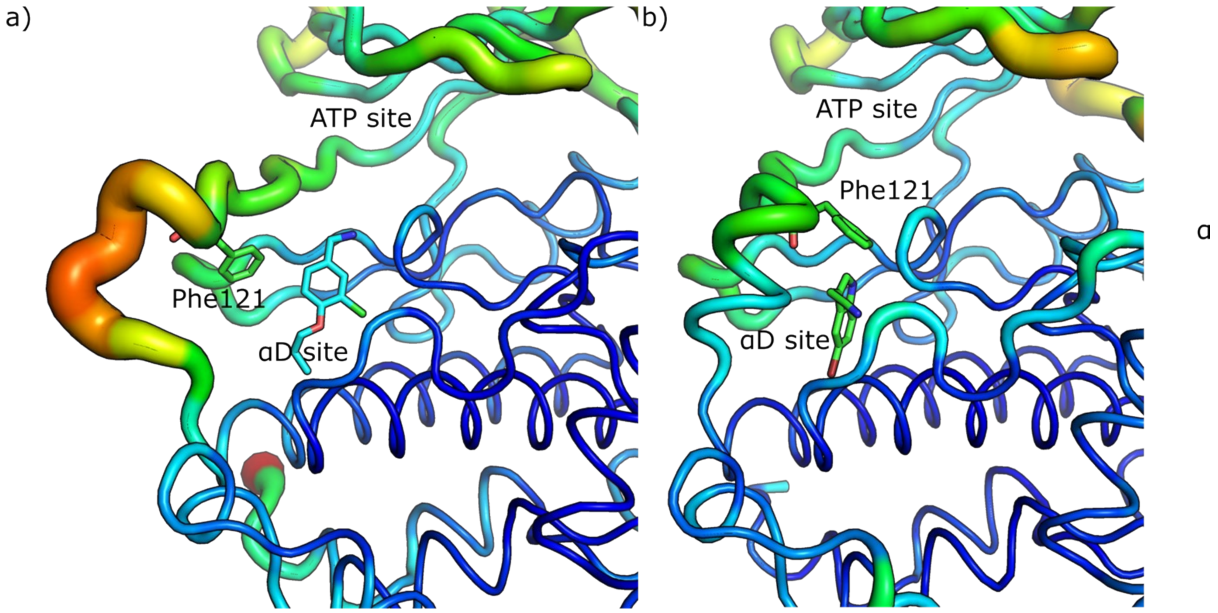

ESI Figure S5 a) The B-factor of PDB entry 5CS6 with a fragment bound in the αD site, causing the αD loop to be disordered. b) The B-factors of **8** bound to the αD site. The αD loop is in the more rigid ordered conformation.

ESI Table S1 Structures of derivatives of fragment **8** alongside their PDB codes, % inhibition at 50 µM, IC_{50}'s and ligand efficiencies. % inhibitions are given as the mean ± SEM from 3 repeats; IC_{50}'s are given as the mean ± SEM from 3 repeats; assays were run with CX4945 as a positive control and DMSO as a negative control; ND = not determined; *under deposition currently.

**Compound**	**Structure**	**PDB**	**% inhibition at 50 µM**	**IC_{50} (µM)**	**Ligand efficiency (kcal mol^{-1})**
**8**	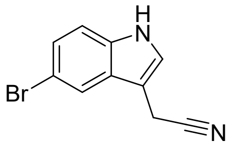	7ZY0	32 ± 7	86 ± 24	0.44
**11**	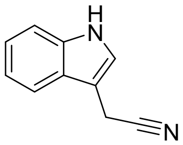	*	5 ± 2	ND	ND
**12**	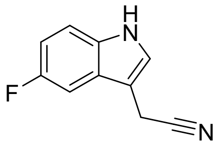	*	28 ± 8	ND	ND
**13**	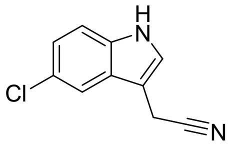	8AEM	21 ± 11	ND	ND
**14**	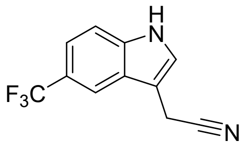	8AEK	39 ± 10	85 ± 5	0.36

ESI Table S2 Structures of fragment **8** derivatives alongside their PDB codes and % inhibition at 50 µM. % inhibitions are given as the mean ± SEM from 3 repeats; assay was run with CX4945 as a positive control and DMSO as a negative control; ND = not determined; *under deposition currently.

Compound	Structure	PDB	% inhibition at 50 µM
**8**	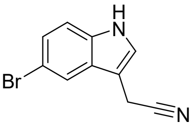	7ZY0	32 ± 7
**18**	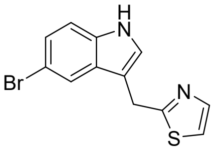	*	13 ± 4
**19**	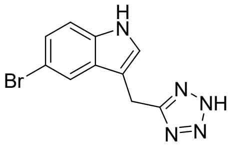	7ZYO	44 ± 4
**20**	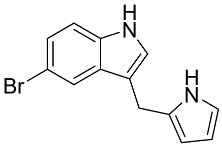	7ZYR	8 ± 3
**21**	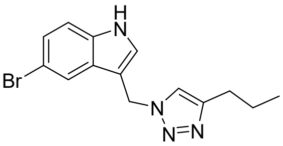	*	11 ± 6
**22**	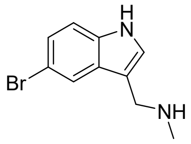	*	21 ± 5
**23**	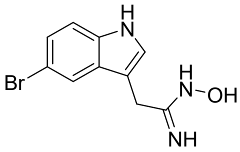	*	13 ± 5
**24**	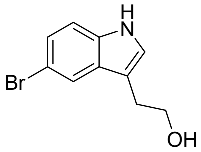	*	35 ± 2
**25**	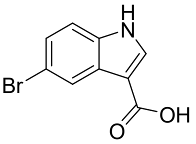	ATP site binder	51 ± 4

**26**	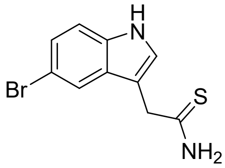	*	42 ± 7

ESI Table 3 Structures of derivatives of **8** alongside their PDB codes, % inhibition at 50 µM, IC_{50}'s, ligand efficiencies and GI_{50}'s. % inhibitions are given as the mean ± SEM from 3 repeats; IC_{50}'s are given as the mean ± SEM from 3 repeats; GI_{50}'s are given ± SEM from three experiments run in triplicate; assays were run with CX4945 as a positive control and DMSO as a negative control. ND = not determined; NS = not soluble under assay conditions; *under deposition currently.

**Compound**	**Structure**	**PDB**	**% inhibition at 50 µM**	**IC_{50} (µM)**	**Ligand efficiency (kcal mol^{-1})**	**GI_{50} (µM)**
**8**	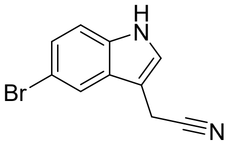	7ZY0	32 ± 7	86 ± 24	0.44	88 ± 7
**9**	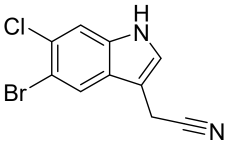	7ZYK	103 ± 6	11 ± 4	0.50	NS
**27**	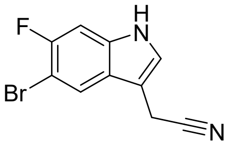	*	29 ± 1	81 ± 1	0.41	ND
**15**	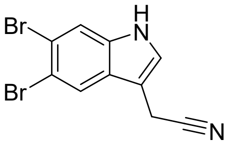	*	65 ± 10	19 ± 15	ND	NS
**10**	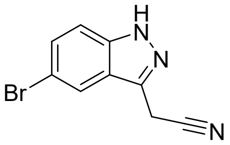	*	97 ± 3	16 ± 4	0.52	NS
**16**	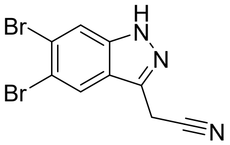	8AE7	95 ± 4	5 ± 1	0.53	NS
**17**	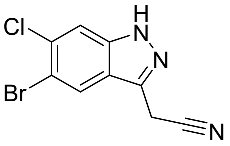	8AEC	98 ± 3	6 ± 1	0.52	NS
**CX4945**	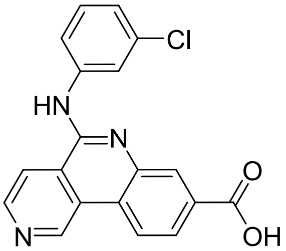	3PE1	102 ± 4	0.001	0.50	4

2. Chemistry experimental

All experiments were carried out in oven-dried glassware under an atmosphere of N_2 using distilled solvents unless otherwise stated.

Reagents: Chemicals were purchased from commercial sources and used without further purification.

Yield: refers to chromatographically and spectroscopically pure compounds unless otherwise stated and are reported as follows: mass, moles, percentage.

Temperature: Reaction temperatures of 0 °C were maintained using an ice-water bath; room temperature (rt) refers to 20-25 °C.

Flash chromatography: Analytical thin layer chromatography was carried out on SiO_2 Merck Kieselgel 60 F254 plates with visualisation either by ultraviolet light or by staining with potassium permanganate or ninhydrin dips made using standard procedures. Retention factors (R_f) are quoted to 0.01. Flash column chromatography was performed using silica gel 60 (230-400 mesh) under a positive pressure of N_2. Eluent systems are expressed in % v/v.

Nuclear Magnetic Resonance (NMR): ^{1}H and ^{13}C NMR spectra were recorded using an internal deuterium lock at ambient probe temperatures on the following instruments: Bruker Avance III 400 MHz HD Smart Probe Spectrometer, Bruker Avance III 400 MHz HD Spectrometer, Bruker 400 MHz QNP Cryoprobe Spectrometer, Bruker 500 MHz DCH Cryoprobe Spectrometer, Bruker Avance III 500 MHz HD Smart Probe Spectrometer. The following deuterated solvents were used: chloroform ($CDCl_3$) and dimethylsulfoxide (DMSO-d_6). ^{1}H-NMR chemical shifts (δ) are quoted in ppm to the nearest 0.01 ppm, relative to the residual non-deuterated solvent peak and coupling constants (*J*) are quoted to the nearest 0.1 Hertz (Hz). ^{13}C-NMR chemical shifts are quoted to the nearest 0.1 ppm, relative to the solvent peak and coupling constants are quoted to the nearest 0.1 Hz. Spectral data is reported as follows: chemical shift, integration, multiplicity (s, singlet; d, doublet; t, triplet; m, multiplet; br, broad; or as a combination of these e.g. br s), coupling constant(s) and assignment. The numbering system used in the assignments does not necessarily follow the IUPAC convention. Assignment of all spectra is supported by DEPT, COSY, HSQC and HMBC or by analogy to fully assigned spectra of closely related compounds.

Infrared spectroscopy (IR): Infrared spectra were recorded neat on a Perkin Elmer Spectrum One FT-IR spectrometer fitted with an Attenuated Total Reflectance (ATR) sampling accessory. Selected absorption maxima (ν_{max}) are quoted in wavenumbers (cm^{-1}) with the following abbreviations: w, weak; m, medium; s, strong; vs, very strong.

Liquid chromatography-mass spectrometry (LCMS): LCMS was carried out using a Waters ACQUITY H-Class UPLC with an ESCi Multi-Mode Ionisation Waters SQ Detector 2 spectrometer using MassLynx 4.1 software; EI refers to the electrospray ionisation technique; LC system: solvent A: 2 mM NH_4OAc in H_2O/MeCN (95:5); solvent B: MeCN; solvent C: 2% formic acid; column: ACQUITY UPLC® CSH C18 (2.1 mm × 50 mm, 1.7 µm, 130 Å) at 40 °C; gradient: 5-95% B with constant 5% C over 1 min at flow rate of 0.6 mL min^{-1}; Injection volume: 5 µL. Chromatographs were monitored by absorbance using diode array detection at a wavelength range of 190-600 nm, interval 1.2 nm.

Analytical HPLC: Chromatographs were obtained on an Agilent 1260 Infinity using a Supelcosil ABZ+PLUS column (150 mm x 4.6 mm, 3 µm) eluting with a linear gradient system (solvent A: 0.05% (v/v) TFA in water, solvent B: 0.05% (v/v) TFA in MeCN) over 15 min, unless otherwise stated, at a flow rate of 1 mL min^{-1}. HPLC was monitored by UV absorbance at 220 and 254 nm.

Preparative HPLC: Preparative HPLC was carried out on an Agilent 1260 Infinity using a Supelcosil ABZ+PLUS column (250 mm x 21.2 mm, 5 µm) eluting with a linear gradient system (solvent A: 0.1% (v/v) TFA in water, solvent B: 0.05% (v/v) TFA in MeCN) over 20 min at a flow rate of 20 mL min^{-1}. HPLC was monitored by UV absorbance at 220 and 254 nm.

Melting points: Melting points were measured using a Büchi melting point B545 apparatus and are uncorrected.

2.1 Experimental synthetic details

General Procedures:

General method 1: Synthesis of indolyl acetonitrile

a) To a solution of the indole (1 equiv) in CH_2Cl_2 (0.13 M) was added *N*,*N*-dimethyl methyleneiminium chloride (1.3 equiv). After the mixture was stirred at room temperature overnight, a 0.1 M aqueous NaOH solution (1.4 equiv) was added. The aqueous solution was extracted with ethyl acetate (x 3) and the organic extracts were dried (Na_2SO_4) and the solvent evaporated under reduced pressure to give the dimethylamine derivative (quantitative yield) that was carried to the following step without further purification.

b) A mixture of the dimethylamine derivative (1 equiv) and NaCN (3 equiv) in EtOAc and DMSO (1:2.5, 0.14 M) was heated at 80 °C for 24 h, then partitioned between EtOAc and H_2O. The organic extract was washed with brine, dried (Na_2SO_4) and the solvent evaporated under reduced pressure. The residue was purified by flash column chromatography over silica gel.

General method 2: Boc-protection

To a solution of the indole/indazole (1 equiv) in CH_2Cl_2 (0.5 M) were added Et_3N (1.3 equiv) and DMAP (0.1 equiv) at 0 ˚C under a nitrogen atmosphere. After 10 min, di-*tert*-butyl dicarbonate (1.3 equiv)

was added. The resulting reaction mixture was stirred at room temperature for 10 h and afterwards washed with brine (x 2), dried ($MgSO_4$), the solvent evaporated, and the mixture purified by flash column chromatography.

2-(5-Bromo-6-chloro-1*H*-indol-3-yl)acetonitrile (**9**)

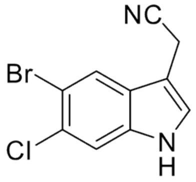

Prepared according to general procedure **1a** with 5-bromo-6-chloro-1*H*-indole (50.0 mg, 0.22 mmol) and *N*,*N*-dimethyl methyleneiminium chloride (26.4 mg, 0.28 mmol) in CH_2Cl_2 (1.6 mL). The dimethylamine derivative (48.9 mg, 0.17 mmol) was treated with NaCN (25.0 mg, 0.51 mmol) according to general procedure **1b**. The crude was purified by flash column chromatography, eluting with a 10-20% gradient of EtOAc in hexane (v/v) to yield the desired product as a white solid (17.3 mg, 38%).

R_f = 0.19 (30% EtOAc/hexane); **Mp** = 147-149 °C; **^{1}H NMR** (500 MHz, $CDCl_3$) δ 8.22 (s, 1H), 7.84 (s, 1H), 7.53 (d, *J* = 0.6 Hz, 1H), 7.27-7.25 (m, 1H, under the solvent peak), 3.79 (t, *J* = 1.0 Hz, 2H); **^{13}C NMR** (126 MHz, $CDCl_3$) δ 135.7, 128.8, 126.4, 124.8, 122.8, 117.6, 114.0, 113.1, 104.8, 14.4; **IR ν_{max}**: 3330, 2988, 2901, 1409, 1057, 829; **HRMS** (ESI^-) m/z found $[M-H]^-$ 266.9331, $C_{10}H_5BrClN_2$ required 266.9325.

5-Bromo-3-(cyanomethyl)-1*H*-indazol-2-ium chloride (**10**)

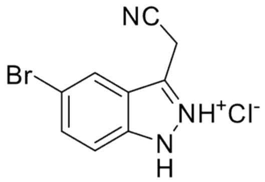

Trimethylsilyl cyanide (10.3 µL, 82.5 µmol) and TBAF (1 M in THF, 82.5 µL, 82.5 µmol) were added to a stirring solution of bromide **28** (21.4 mg, 55.0 µmol) in 550 µL of MeCN under an atmosphere of nitrogen. After consumption of the starting material (16 h, monitored by TLC), the light-yellow reaction mixture was concentrated *in vacuo*, and the resulting residue was purified by flash column chromatography, eluting with a 5-20% EtOAc in hexane (v/v) (R_f = 0.37, 20% EtOAc/hexane). The Boc-protected material, dissolved in the minimum amount of CH_2Cl_2, was treated with HCl (2 M in Et_2O, 275 µL, 550 µmol) and heated at 40 ˚C overnight. After cooling the mixture to rt, the precipitate was filtered and washed with cold Et_2O to yield the desired compound as a brown solid (13.4 mg, 90%).

Mp = 115-116 °C; **^{1}H NMR** (500 MHz, MeOD) δ 8.02 (dd, *J* = 1.7, 0.8 Hz, 1H), 7.52 (dd, *J* = 8.9, 1.7 Hz, 1H), 7.48 (dd, *J* = 8.9, 0.8 Hz, 1H), 4.23 (s, 2H); **^{13}C NMR** (126 MHz, MeOD) δ 141.5, 136.2, 131.2, 123.7,

123.0, 118.0, 114.9, 113.2, 16.3; **IR ν_{max}:** 3322, 2918, 15472, 1040, 803; **HRMS** (ESI^+) m/z found $[M+Na]^+$ 257.9626, $C_9H_6BrN_3Na$ required 257.9643.

2-(5-(Trifluoromethyl)-1*H*-indol-3-yl)acetonitrile (**14**)

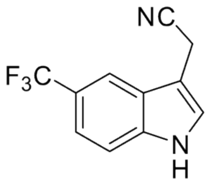

Prepared according to general procedure **1a** with 5-trifluoromethyl-1*H*-indole (100 mg, 0.54 mmol) and *N*,*N*-dimethyl methyleneiminium chloride (66.0 mg, 0.70 mmol) in CH_2Cl_2 (4.0 mL). The dimethylamine derivative (125 mg, 0.52 mmol) was treated with NaCN (75.9 mg, 1.55 mmol) according to general procedure **1b**. The crude was purified by flash column chromatography, eluting with a 10-20% gradient of EtOAc in hexane (v/v) to yield the desired product as a brown solid (76.0 mg, 66%).

R_f = 0.28 (30% EtOAc/hexane); **Mp** = 119-122 °C; **^{1}H NMR** (400 MHz, $CDCl_3$) δ 8.44 (s, 1H), 7.88 (s, 1H), 7.49 (m, 2H), 7.40-7.32 (m, 1H), 3.87 (d, *J* = 0.9 Hz, 2H); **^{13}C NMR** (101 MHz, $CDCl_3$) δ 137.8, 126.5, 125.5, 124.7, 123.0 (q, *J* = 32.0 Hz), 119.9 (q, *J* = 3.4 Hz), 117.8, 116.0 (q, *J* = 4.3 Hz), 112.1, 106.1, 14.4; **IR ν_{max}:** 3339, 2988, 1107, 1075; **HRMS** (ESI^+) m/z found $[M+H]^+$ 225.0639, $C_{11}H_8F_3N_2$ required 225.0640.

2-(5,6-Dibromo-1*H*-indol-3-yl)acetonitrile (**15**)

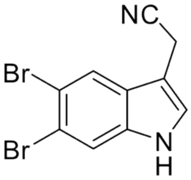

Prepared according to general procedure **1a** with 5,6-dibromo-1*H*-indole (124 mg, 0.45 mmol) and *N*,*N*-dimethyl methyleneiminium chloride (54.7 mg, 0.585 mmol) in CH_2Cl_2 (3.36 mL). The dimethylamine derivative (75.0 mg, 0.28 mmol) was treated with NaCN (41.2 mg, 0.84 mmol) according to general procedure **1b**. The crude was purified by flash column chromatography, eluting with a 10-20% gradient of EtOAc/hexane (v/v) to yield the desired product as a yellow solid (23.4 mg, 27%).

R_f = 0.29 (30% EtOAc/hexane); **Mp** = 164-166 °C; **^{1}H NMR** (400 MHz, $CDCl_3$) δ 8.21 (br s, 1H), 7.85 (s, 1H), 7.70 (s, 1H), 7.26-7.24 (m, 1H), 3.79 (s, 2H); **^{13}C NMR** (101 MHz, $CDCl_3$) δ 136.0, 127.0, 124.8, 122.7, 118.6, 117.6, 116.4, 116.0, 104.8, 14.4; **IR ν_{max}:** 3329, 1447, 1407, 1105, 860, 830; **HRMS** (ESI^-) m/z found $[M-H]^-$ 310.8826, $C_{10}H_5Br_2N_2$ required 310.8819.

Tert-butyl 6-bromo-3-methyl-1*H*-indazole-1-carboxylate (**32**)

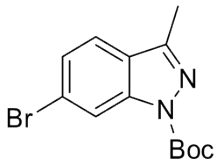

Prepared according to general procedure **2** with 6-bromo-3-methyl-1*H*-indazole (3.16 g, 15.0 mmol), CH_2Cl_2 (30 mL), Et_3N (2.72 mL, 19.5 mmol), DMAP (183 mg, 1.5 mmol) and di-*tert*-butyl dicarbonate (4.25 g, 19.5 mmol). The residue was purified by flash column chromatography, eluting with a 5-10% gradient of EtOAc/hexane (v/v) to give the pure product as a white solid (4.35 g, 93%).

R_f = 0.18 (5% EtOAc/hexane); **Mp** = 113-115 ˚C; **^{1}H NMR** (500 MHz, $CDCl_3$) δ 8.33 (br s, 1H), 7.49 (d, *J* = 8.4 Hz, 1H), 7.41 (dd, *J* = 8.4, 1.5 Hz, 1H), 2.57 (s, 3H), 1.71 (s, 9H); **^{13}C NMR** (126 MHz, $CDCl_3$) δ 149.1, 148.5, 141.1, 126.9, 124.9, 123.7, 121.4, 118.0, 85.2, 28.3, 12.4; **IR ν_{max}:** 1759, 1731, 1605, 1366, 1081, 786; **HRMS** ($ASAP^+$) m/z found $[M\text{-}Boc+H]^+$ 210.9870, $C_8H_8BrN_2$ required 210.9871.

Data in accordance with the literature.[1]

Tert-butyl 6-bromo-3-(bromomethyl)-1*H*-indazole-1-carboxylate (**33**)

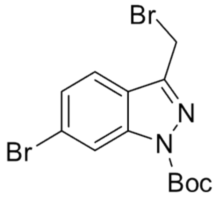

Indazole **32** (2.18 g, 7.0 mmol) was suspended in CCl_4 (70 mL), NBS (1.87g, 10.5 mmol) was added and the solution was degassed with a stream of nitrogen for 15 min. AIBN (230 mg, 1.4 mmol) was then added and the solution stirred at reflux for 18 h. The mixture was cooled to rt and the solvent evaporated *in vacuo*. The residue was purified by flash column chromatography, eluting with a 2-4% gradient of EtOAc/hexane (v/v) give the desired product as a white solid (1.73 g, 63%).

R_f = 0.49 (10% EtOAc/hexane); **Mp** 89-91 ˚C; **^{1}H NMR** (500 MHz, $CDCl_3$) δ 8.37 (s, 1H), 7.71 (d, *J* = 8.4 Hz, 1H), 7.49 (dd, *J* = 8.5, 1.6 Hz, 1H), 4.75 (s, 2H), 1.72 (s, 9H); **^{13}C NMR** (126 MHz, $CDCl_3$) δ 148.7, 147.2, 141.6, 127.5, 124.2, 123.0, 121.9, 118.2, 86.1, 28.2, 22.6; **IR ν_{max}:** 1773, 1308, 1147, 1083, 844, 738; **HRMS** (ESI^+) m/z found $[M\text{-}Boc+H]^+$ 288.8988, $C_8H_7Br_2N_2$ required 288.8976.

2-(6-Bromo-1*H*-indazol-3-yl)acetonitrile (**34**)

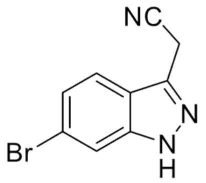

Trimethylsilyl cyanide (165 µL, 1.32 mmol) and TBAF (1 M in THF, 1.20 mL, 1.20 mmol) were added to a stirring solution of bromide **33** (468 mg, 1.20 mmol) in MeCN (12 mL) under an atmosphere of nitrogen. The mixture was stirred at rt for 2 h and then the solvent was removed *in vacuo*. The resulting residue was purified by flash column chromatography, eluting with a 10-20% gradient of EtOAc/hexane (v/v) (R_f = 0.31, 20% EtOAc/hexane) to give the desired compound as a white solid (293 mg, 72%). This material (102 mg, 0.30 mmol) was treated with a 1 M aqueous solution of KOH (0.60 µL, 0.60 mmol) in MeOH (1.50 mL) for 30 min and then the solvent was removed *in vacuo*. The residue was purified by flash column chromatography, eluting with a 10-20% gradient of EtOAc/hexane (v/v) providing the desired compound as a white solid (70 mg, 99%).

R_f = 0.28 (30% EtOAc/hexane); **Mp** 169-171 ˚C; **^{1}H NMR** (500 MHz, MeOD) δ 7.86-7.58 (m, 2H), 7.37-6.99 (m, 1H), 4.22 (s, 2H); **^{13}C NMR** (126 MHz, MeOD) δ 143.5, 137.1, 125.5, 122.4, 121.9, 121.1, 118.0, 114.3, 16.3; **IR ν_{max}:** 3370, 1611, 046, 873, 785, 667; **HRMS** (ASAP$^+$) m/z found $[M+H]^+$ 235.9817, $C_9H_7BrN_3$ required 235.9823.

2-(5,6-Dibromo-1*H*-indazol-3-yl)acetonitrile (**16**)

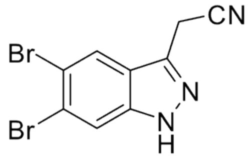

To a solution of compound **34** (50.0 mg, 0.21 mmol) in DMF (420 µL) was added NBS (37.4 mg, 0.21 mmol) and the reaction stirred at rt for 4 h. After the solvent was evaporated, the residue was triturated with EtOAc and filtered. The filtrate was purified by preparative HPLC, eluting with a 39-47% gradient of MeCN (0.05% TFA)/H_2O (0.1% TFA) (v/v) in 22 min, affording the desired product as a white solid (33.9 mg, 55%).

T_r = 17.0 min; **Mp** = 198-200 ˚C; **^{1}H NMR** (500 MHz, DMSO-*d$_6$*) δ 13.38 (s, 1H), 8.31 (s, 1H), 8.02 (s, 1H), 4.40 (s, 1H); **^{13}C NMR** (126 MHz, DMSO-*d$_6$*) δ 140.4, 135.3, 124.3, 121.9, 121.6, 117.7, 115.4, 114.9, 15.4; **IR ν_{max}:** 3363, 1608, 1410, 1204, 1055, 667, 618; **HRMS** (ESI$^+$) m/z found $[M+H]^+$ 313.8929, $C_9H_6Br_2N_3$ required 313.8928.

Tert-butyl 6-chloro-3-methyl-1*H*-indazole-1-carboxylate (**35**)

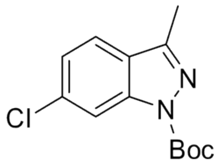

1-(4-Chloro-2-fluorophenyl)ethan-1-one (1.37 mL, 10.0 mmol) was dissolved in ethylene glycol (20 mL) and hydrazine monohydrate (970 µL, 20.0 mmol) was added. The mixture was stirred at rt for 30 min followed by 1 h at 200 ˚C under microwave irradiation. The reaction was quenched with brine and extracted with CH_2Cl_2. The organic layer was dried (Na_2SO_4) and the solvent evaporated to provide 6-chloro-3-methyl-1*H*-indazole as a light brown solid (1.58 g, 95%). The Boc-protected derivative was prepared according to general procedure **2** with the 6-chloro indazole derivative (1.58 g, 9.50 mmol), CH_2Cl_2 (19.0 mL), Et_3N (1.72 mL, 12.4 mmol), DMAP (116 mg, 0.95 mmol) and di-*tert*-butyl dicarbonate (2.70 g, 12.4 mmol). The residue was purified by flash column chromatography, eluting with a 10-15% gradient of EtOAc/hexane (v/v) to give the pure product as a white solid (2.26 g, 89%).

R_f = 0.34 (15% EtOAc/hexane); **Mp** = 92-94 ˚C; **^{1}H NMR** (400 MHz, $CDCl_3$) δ 8.15 (s, 1H), 7.55 (dd, *J* = 8.4, 0.5 Hz, 1H), 7.27 (dd, *J* = 8.4, 1.7 Hz, 1H, under the solvent peak), 2.57 (s, 3H), 1.72 (s, 9H); **^{13}C NMR** (101 MHz, $CDCl_3$) δ 149.1, 148.4, 140.8, 135.5, 124.6, 124.3, 121.2, 115.0, 85.2, 28.3, 12.4; **IR ν_{max}:** 2978, 1754, 1723, 1364, 1143, 1084, 849, 793; **HRMS** (ESI$^+$) m/z found $[M+H]^+$ 267.0904, $C_{13}H_{16}ClN_2O_2$ required 267.0900.

Tert-butyl 3-(bromomethyl)-6-chloro-1*H*-indazole-1-carboxylate (**36**)

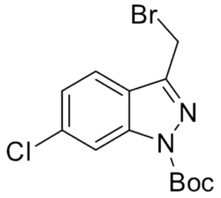

Indazole **35** (2.00 g, 7.50 mmol) was suspended in CCl_4 (75 mL), NBS (2.00 g, 11.2 mmol) was added and the solution was degassed with a stream of nitrogen gas for 15 min. AIBN (246 mg, 1.5 mmol) was then added and the solution stirred at reflux for 18 h. The mixture was cooled to rt and the solvent removed *in vacuo*. The residue was purified by flash column chromatography, eluting with a 2-4% gradient of EtOAc/hexane (v/v) to give the desired product as a white solid (1.38 g, 53%).

R_f = 0.63 (10% EtOAc/hexane); **Mp** = 94-96 ˚C; **^{1}H NMR** (500 MHz, $CDCl_3$) δ 8.18 (s, 1H), 7.77 (dd, *J* = 8.5, 0.7 Hz, 1H), 7.35 (dd, *J* = 8.5, 1.8 Hz, 1H), 4.75 (s, 2H), 1.72 (s, 9H); **^{13}C NMR** (126 MHz, $CDCl_3$) δ 148.7, 147.2, 141.4, 136.0, 124.9, 122.7, 121.7, 115.2, 86.1, 28.2, 22.6; **IR ν_{max}:** 1745, 1392, 1319, 1145, 1084, 802; **HRMS** (ESI$^+$) m/z found $[M+Na]^+$ 366.9822, $C_{13}H_{14}BrClN_2O_2Na$ required 366.9825

2-(6-Chloro-1*H*-indazol-3-yl)acetonitrile (**37**)

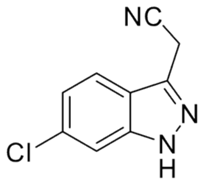

Trimethylsilyl cyanide (275 µL, 2.20 mmol) and TBAF (1 M in THF, 2.00 mL, 2.00 mmol) were added to a stirring solution of bromide **36** (691 mg, 2.00 mmol) in MeCN (20 mL) under an atmosphere of nitrogen. The mixture was stirred at rt for 2 h and then the solvent was removed *in vacuo*. The resulting residue was purified by flash column chromatography, eluting with a 10-20% gradient of EtOAc/hexane (v/v) (R_f = 0.24, 15% EtOAc/hexane) to give the desired compound as a white solid (336 mg, 57%). This material (204 mg, 0.70 mmol) was treated with a 1 M aqueous solution of KOH (1.40 mL, 1.40 mmol) in MeOH (3.50 mL) for 30 min and then the solvent was removed *in vacuo*. The residue was purified by flash column chromatography, eluting with a 20-30% gradient of EtOAc/hexane (v/v) providing the desired compound as a white solid (115 mg, 86%).

R_f = 0.18 (25% EtOAc/hexane); **Mp** = 171-173 ˚C; **^{1}H NMR** (500 MHz, MeOD) δ 7.80 (dd, *J* = 8.7, 0.7 Hz, 1H), 7.56 (dd, *J* = 1.7, 0.7 Hz, 1H), 7.19 (dd, *J* = 8.7, 1.7 Hz, 1H), 4.58 (s, 2H); **^{13}C NMR** (126 MHz, MeOD) δ 143.2, 137.0, 134.5, 123.0, 121.8, 120.9, 118.0, 111.1, 16.1; **IR ν_{max}:** 3373, 1612, 1336, 1046, 785, 657; **HRMS** (ESI$^+$) m/z found $[M+H]^+$ 192.0326, $C_9H_7ClN_3$ required 192.0328.

2-(5-Bromo-6-chloro-1*H*-indazol-3-yl)acetonitrile (**17**)

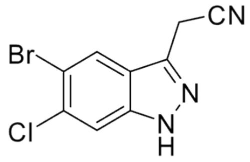

To a solution of compound **37** (50.8 mg, 0.265 mmol) in DMF (530 µL) was added NBS (47.2 mg, 0.265 mmol) and the reaction was stirred at rt for 4 h, after which the solvent was removed *in vacuo.* The residue was then triturated in EtOAc and filtered. The filtrate was purified by preparative HPLC eluting with a 40-50% gradient of MeCN (0.05% TFA)/H_2O (0.1% TFA) (v/v) in 22 min affording the desired product as a white solid (24.1 mg, 34%).

T_r = 14.1 min; **Mp** > 267 ˚C decomposition; **^{1}H NMR** (500 MHz, MeOD) δ 9.77 (s, 1H), 9.33-9.30 (m, 1H), 5.79 (s, 2H); **^{13}C NMR** (126 MHz, MeOD) δ 142.0, 136.5, 134.0, 125.4, 122.4, 117.8, 115.2, 112.9, 16.3; **IR ν_{max}:** 3372, 1612, 1414, 1319, 1231, 941, 670; **HRMS** (ASAP$^+$) m/z found $[M+H]^+$ 269.9429, $C_9H_6BrClN_3$ required 269.9434.

2-(5-Bromo-1*H*-indol-3-yl)ethanethioamide (**26**)

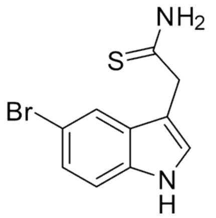

A mixture of 2-(5-bromo-1*H*-indol-3-yl)acetonitrile (129 mg, 0.55 mmol), diethyldithiophosphoric acid (115 µL, 0.66 mmol) in THF (2.75 mL) was heated at 80 °C under microwave irradiation. Further diethyldithiophosphoric acid (115 µL, 0.66 mmol) was added until the starting material was consumed (3 additions in 3 h). The reaction mixture was extracted with Et_2O (x 3) and the organic layers were washed with saturated $NaHCO_3$. The combined organic layers were dried ($MgSO_4$) and the solvent removed *in vacuo* to give the product as an off-yellow solid (109 mg, 74%).

Mp = 161-163 °C; **^{1}H NMR** (400 MHz, MeOD) δ 7.75 (d, *J* = 1.8 Hz, 1H), 7.30-7.23 (m, 2H), 7.19 (dd, *J* = 8.6, 1.8 Hz, 1H), 4.08 (s, 2H); **^{13}C NMR** (101 MHz, MeOD) δ 209.4, 136.8, 130.2, 126.7, 125.4, 122.3, 114.0, 113.2, 110.6, 42.8; **IR ν_{max}:** 3393, 2989, 1615, 1445, 1445, 1095, 1045, 795; **HRMS** (ESI^+) m/z found $[M+H]^+$ 268.9767, $C_{10}H_{10}BrN_2S$ required 268.9748.

2-((5-Bromo-1*H*-indol-3-yl)methyl)thiazole (**18**)

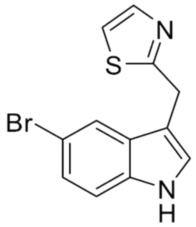

A mixture of carbothioamide **26** (26.9 mg, 0.10 mmol), 2-bromo-1,1-diethoxyethane (30.1 µL, 0.20 mmol), EtOH (110 µL) and *N*,*N*-dimethylacetamide (110 µL) was stirred at 100 °C for 4 h. The reaction mixture was cooled to rt, H_2O added and the resulting crystals were filtered, washed with H_2O and hexane and dried to give the title compound as yellow crystals (11.4 mg, 39%).

Mp = 152-154 ˚C; **^{1}H NMR** (500 MHz, DMSO-*d$_6$*) δ 11.22 (s, 1H), 7.70 (d, *J* = 3.3 Hz, 1H), 7.64 (d, *J* = 1.9 Hz, 1H), 7.51 (d, *J* = 3.3 Hz, 1H), 7.40 (d, *J* = 2.4 Hz, 1H), 7.34 (d, *J* = 8.6 Hz, 1H), 7.19 (dd, *J* = 8.6, 1.9 Hz, 1H), 4.42 (s, 2H); **^{13}C NMR** (126 MHz, DMSO-*d$_6$*) δ 171.0, 142.1, 135.0, 128.6, 125.7, 123.6, 120.8, 119.7, 113.6, 111.2, 111.0, 28.6; **IR ν_{max}:** 3159, 1678, 1134, 723; **HRMS** (ESI^+) m/z found $[M+H]^+$ 292.9738, $C_{12}H_{10}BrN_2S$ required 292.9748.

3-((1*H*-Tetrazol-5-yl)methyl)-5-bromo-1*H*-indole (**19**)

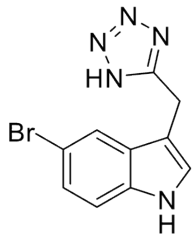

Anhydrous $AlCl_3$ (28.0 mg, 0.21 mmol) was suspended in dry THF (200 µL) at 0 °C. NaN_3 (36.4 mg, 0.56 mmol) was added and the off-white suspension refluxed for 2 h. Upon brief cooling, 2-(5-bromo-1*H*-indol-3-yl)acetonitrile (47.0 mg, 0.2 mmol) was added. The reaction mixture was stirred at reflux until consumption of the starting material. The resulting suspension was cooled to rt, poured into 1 M aqueous citric acid and extracted with EtOAc (x 3). The combined organic layers were washed with brine, dried (Na_2SO_4) and the solvent removed *in vacuo*. Purification by flash column chromatography, eluting with a 2-10% gradient of MeOH/CH_2Cl_2 (v/v) furnished the title product as an off-white solid (44.8 mg, 80%)

R_f = 0.12 (5% MeOH/CH_2Cl_2); **Mp** = 210-212 °C; **^{1}H NMR** (500 MHz, MeOD) δ 7.57 (dd, *J* = 1.9, 0.5 Hz, 1H), 7.29 (dd, *J* = 8.6, 0.6 Hz, 1H), 7.25-7.19 (m, 2H), 4.41 (d, *J* = 0.8 Hz, 2H); **^{13}C NMR** (126 MHz, MeOD) δ 157.9, 136.8, 129.8, 126.2, 125.6, 121.6, 114.2, 113.4, 109.2, 20.6; **IR v_{max}:** 3251, 1722, 1414, 1233, 1047, 882, 796; **HRMS** (ESI^-) m/z found $[M-H]^-$ 275.9893, $C_{10}H_7BrN_5$ required 275.9885

3-((1*H*-Pyrrol-2-yl)methyl)-5-bromo-1*H*-indole (**20**)

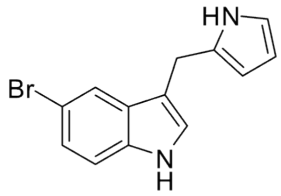

The dimethylamine derivative was prepared according to general procedure **1a** with 5-bromo-1*H*-indole (367 mg, 1.87 mmol), *N*,*N*-dimethyl methyleneiminium chloride (227 mg, 2.43 mmol), CH_2Cl_2 (14.0 mL). This material (50.6 mg, 0.20 mmol) in pyrrole (0.80 µL) was heated to 150 ˚C under microwave irradiation for 30 min. The reaction mixture was cooled to rt and the solvent removed *in vacuo*. The brown oily residue was purified by flash column chromatography, eluting with 10% EtOAc/hexane (v/v) to afford the product as a light brown solid (32.0 mg, 58%)

R_f = 0.48 (30% EtOAc/hexane); **Mp** = 120-122 ˚C; **^{1}H NMR** (500 MHz, MeOD) δ 7.51 (dd, *J* = 1.9, 0.5 Hz, 1H), 7.23 (dd, *J* = 8.6, 0.5 Hz, 1H), 7.17-7.11 (m, 1H), 7.03 (s, 1H), 6.59 (dt, *J* = 2.8, 1.5 Hz, 1H), 5.98 (t, *J* = 3.0 Hz, 1H), 5.87-5.82 (m, 1H), 4.00 (t, *J* = 0.7 Hz, 2H); **^{13}C NMR** (126 MHz, MeOD) δ 136.9, 132.0, 130.5, 125. 1, 124.9, 122.3, 117.2, 114.8, 113.7, 112.6, 108.2, 106.2, 24.6; **IR v_{max}:** 3398, 3346, 1443,790, 724; **HRMS** (ESI^+) m/z found $[M+H]^+$ 275.0109, $C_{13}H_{12}BrN_2$ required 275.0117.

Tert-butyl 5-bromo-3-((dimethylamino)methyl)-1*H*-indole-1-carboxylate (**30**)

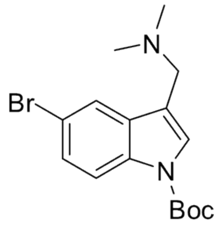

The dimethylamine derivative was prepared according to general procedure **1a** with 5-bromo-1*H*-indole (3.67 g, 18.7 mmol), *N*,*N*-dimethyl methyleneiminium chloride (2.27 g, 24.3 mmol) and CH_2Cl_2 (140 mL). The Boc-protected derivative was prepared according to general procedure **2** with the dimethylamine derivative (1.26 g, 5.0 mmol), CH_2Cl_2 (10 mL), Et_3N (657 µL, 6.5 mmol), DMAP (61 mg, 0.50 mmol) and di-*tert*-butyl dicarbonate (1.42 mg, 6.5 mmol). The residue was purified by flash column chromatography, eluting with 2% MeOH/CH_2Cl_2 (v/v) to give the Boc-protected compound as a yellow viscous oil (1.36 g, 77%).

R_f = 0.19 (5% MeOH/CH_2Cl_2); **^{1}H NMR** (400 MHz, $CDCl_3$) δ 8.00 (d, *J* = 7.9 Hz, 1H), 7.81 (d, *J* = 2.0 Hz, 1H), 7.48 (s, 1H), 7.39 (dd, *J* = 8.8, 2.0 Hz, 1H), 3.48 (s, 2H), 2.27 (s, 6H), 1.65 (s, 9H); **^{13}C NMR** (101 MHz, $CDCl_3$) δ 149.5, 134.5, 132.4, 127.3, 125.5, 122.6, 117.8, 116.7, 116.1, 84.1, 54.6, 45.7, 28.3; **IR ν_{max}:** 1731, 1447, 1154, 1092, 1007, 800; **HRMS** (ESI$^+$) m/z found $[M+H]^+$ 353.0828, $C_{16}H_{22}BrN_2O_2$ required 353.0865.

Tert-butyl 3-(azidomethyl)-5-bromo-1*H*-indole-1-carboxylate (**31**)

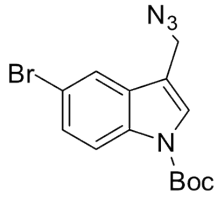

To a solution of **30** (680 mg, 1.92 mmol) in Et_2O (7.70 mL), MeI (360 µL, 5.78 mmol) was added. The resulting reaction mixture was stirred at rt for 8 h. The suspension was then filtered off and washed with dry Et_2O to give the trimethylammonium salt as a pale pink solid (879 mg, 92%). Trimethylsilylazide (239 µL, 1.82 mmol) and TBAF (1 M in THF, 1.82 mL, 1.82 mmol) were added to a stirring solution of ammonium salt (600 mg, 1.21 mmol) in THF (12.1 mL) and the mixture was stirred overnight at 60 ˚C. The light-yellow reaction mixture was concentrated *in vacuo*, and the resulting residue purified by flash column chromatography, eluting with 1% Et_2O/hexane (v/v) to provide the desired compound as a white solid (300 mg, 70%).

R_f = 0.19 (2% Et_2O/hexane); **Mp** = 97-99 ˚C; **^{1}H NMR** (500 MHz, $CDCl_3$) δ 8.03 (d, *J* = 8.1 Hz, 1H), 7.73 (dd, *J* = 2.0, 0.5 Hz, 1H), 7.54 (d, *J* = 73.9 Hz, 2H), 7.45 (ddd, *J* = 8.8, 2.0, 0.3 Hz, 1H), 4.43 (d, *J* = 0.8 Hz, 2H), 1.67 (s, 9H); **^{13}C NMR** (126 MHz, $CDCl_3$) δ 149.2, 134.6, 130.8, 128.0, 126.1, 122.0, 117.0, 116.6, 114.3, 84.8, 46.1, 28.3; **IR ν_{max}:** 2091, 1728, 1367, 1151, 1095, 807.

5-Bromo-3-((4-propyl-1*H*-1,2,3-triazol-1-yl)methyl)-1*H*-indole (**21**)

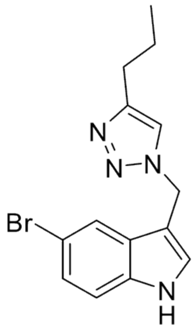

1-Pentyne (39.4 µL, 0.40 mmol) was dissolved in a 1:1:1 mixture of ^{t}BuOH, H_2O and CH_2Cl_2 (5.45 mL). To this solution, azide **31** (112 mg, 0.32 mmol) was added while stirring vigorously at rt. Argon was bubbled through the solution for 30 min after which copper (II) sulfate pentahydrate (100 mg, 0.40 mmol), sodium ascorbate (238 mg, 1.20 mmol) and THTPA (86.9 mg, 0.20 mmol) were added sequentially to the solution. The reaction mixture was allowed to stir until completion, monitored *via* TLC analysis (18 h). The suspension was extracted with CH_2Cl_2, the organic layer dried (Na_2SO_4) and concentrated *in vacuo*, and the residue was purified by flash column chromatography, eluting with a 20-30% gradient of EtOAc/hexane (v/v) (R_f = 0.48, 50% EtOAc/hexane) to give the pure product as a pale pink solid (85.8 mg, 51%). The residue (41.9 mg, 0.10 mmol) was suspended in CH_2Cl_2 (1.40 mL) and treated with TFA (300 µL, 0.30 mmol) for 3 h at rt after which time the solvent was removed *in vacuo.* The resulting residue was purified by flash column chromatography, eluting with 1% MeOH/CH_2Cl_2 (v/v) to furnish the desired compound as a pale pink solid (28mg, 88%).

R_f = 0.27 (2% MeOH/CH_2Cl_2); **Mp** = 142-144 ˚C; **^{1}H NMR** (500 MHz, MeOD) δ 7.58 (s, 1H), 7.46-7.42 (m, 1H), 7.30 (dd, *J* = 8.6, 0.6 Hz, 1H), 7.21 (ddd, *J* = 8.7, 1.9, 0.3 Hz, 1H), 5.67 (d, *J* = 0.6 Hz, 2H), 2.62-2.57 (m, 2H), 1.66-1.58 (m, 2H), 0.90 (t, *J* = 7.4 Hz, 3H); **^{13}C NMR** (126 MHz, MeOD) δ 149.3, 136.9, 129.3, 127.6, 125.9, 122.7, 121.8, 114.3, 113.8, 110.1, 46.7, 28.3, 23.7, 13.9; **IR v_{max}:** 2956, 2918, 1455,1056, 863, 783; **HRMS** (ESI$^+$) m/z found $[M+H]^+$ 319.0545, $C_{14}H_{16}BrN_4$ required 319.0558.

1-(5-Bromo-1*H*-indol-3-yl)-*N*-methylmethanaminium chloride (**22**)

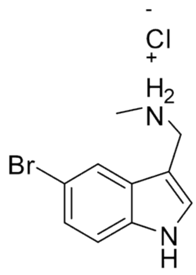

5-Bromo-1*H*-indole-3-carbaldehyde (67.2 mg, 0.30 mmol) and methylamine (225 µL, 2 M in MeOH, 0.45 mmol) were combined in anhydrous 1,2-dichloroethane (1.07 mL) under an atmosphere of nitrogen and stirred for 2 h. Sodium triacetoxyborohydride (89.0 mg, 0.42 mmol) was added in two

portions with a 30 min interval and the reaction was stirred at rt for 18 h. The reaction mixture was poured into 2 M aqueous Na_2CO_3 and extracted with CH_2Cl_2 (x 3). The combined organic extracts were dried (Na_2SO_4), the solvent removed *in vacuo* and purified by flash column chromatography, eluting with CH_2Cl_2/MeOH/NH_3 90:9:1 (R_f = 0.19, 90:9:1 CH_2Cl_2/MeOH/NH_3). The residue (33.5 mg) was dissolved in the minimum amount of CH_2Cl_2 and treated with HCl (2 M in Et_2O, 700 µL, 1.41 mmol). The precipitate was filtered and washed with cold Et_2O to yield the desired compound as an off-white solid (35 mg, 42%).

Mp = 154-156 °C; **^{1}H NMR** (500 MHz, MeOD) δ 7.87 (dd, *J* = 1.9, 0.5 Hz, 1H), 7.48 (s, 1H), 7.34 (dd, *J* = 8.7, 0.5 Hz, 1H), 7.26 (dd, *J* = 8.7, 1.9 Hz, 1H), 4.23 (s, 2H), 2.61 (s, 3H); **^{13}C NMR** (126 MHz, MeOD) δ 136.7, 129.9, 128.8, 125.9, 121.8, 114.3, 114.0, 107.9, 45.0, 33.3; **IR ν_{max}:** 3214, 1459, 1440, 1059; **HRMS** (ESI^-) m/z found [M] 238.0110, $C_{10}H_{11}BrN_2$ required 238.0106.

2-(5-Bromo-1*H*-indol-3-yl)-*N*-hydroxyacetimidamide (**23**)

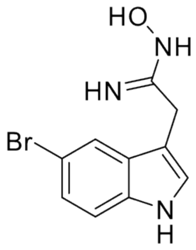

A stirred mixture of NaOMe (59.2 mg, 1.10 mmol), hydroxylamine hydrochloride (76.4 mg, 1.10 mmol) and MeOH (860 µL) was heated under reflux for 30 min. The mixture was cooled to rt before 2-(5-bromo-1*H*-indol-3-yl)acetonitrile (235 mg, 1.00 mmol) was added and heating was continued for 18 h. The mixture was concentrated and partitioned between H_2O and Et_2O. The organic phase was dried (Na_2SO_4) and the solvent evaporated to give the product as a pale yellow solid (132 mg, 49%).

Mp > 170 °C (decomp.); **^{1}H NMR** (500 MHz, DMSO-*d$_6$*) δ 12.67 (s, 1H), 11.38 (s, 1H), 10.87 (s, 1H), 7.92 (d, *J* = 1.7 Hz, 1H), 7.48 (d, *J* = 2.4 Hz, 1H), 7.37 (d, *J* = 8.6 Hz, 1H), 7.22 (dd, *J* = 8.6, 1.9 Hz, 1H), 3.80 (s, 2H); **^{13}C NMR** (126 MHz, DMSO-*d$_6$*) δ 161.8, 134.7, 128.2, 126.9, 123.9, 120.6, 113.7, 111.7, 105.8, 24.8; **IR ν_{max}:** 3263, 1678, 780, 635; **HRMS** (ESI^+) m/z found $[M+H]^+$ 268.0028, $C_{10}H_{11}BrN_3O$ required 268.0085.

Data in accordance with the literature.[2]

2-(5-Bromo-6-fluoro-1*H*-indol-3-yl)acetonitrile (**27**)

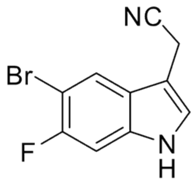

The title compound was prepared according to general procedure **1a** with 5-bromo-6-fluoro-1*H*-indole (50.0 mg, 0.23 mmol) and *N*,*N*-dimethyl methyleneiminium chloride (28.4 mg, 0.30 mmol) in CH_2Cl_2 (1.7 mL). The dimethylamine derivative (40.1 mg, 0.15 mmol) was treated with NaCN (22.0 mg, 0.45 mmol) according to general procedure **1b**. The crude was purified by flash column chromatography, eluting with a 10-20% gradient of EtOAc/hexane (v/v) to yield the desired product as an off-white solid (29.0 mg, 76%).

R$_f$ = 0.17 (30% EtOAc/hexane); **Mp** = 141-143 °C; **^{1}H NMR** (500 MHz, $CDCl_3$) δ 8.21 (s, 1H), 7.74 (d, *J* = 6.5 Hz, 1H), 7.26-7.24 (m, 1H), 7.18 (d, *J* = 8.8 Hz, 1H), 3.79 (d, *J* = 1.1 Hz, 2H); **^{13}C NMR** (126 MHz, $CDCl_3$) δ 156.0 (d, *J* = 240.6 Hz), 135.4 (d, *J* = 11.2 Hz), 124.2, 123.9, 122.3, 117.7, 104.8, 102.2 (d, *J* = 23.6 Hz), 99.2 (d, *J* = 27.6 Hz), 14.5; **IR ν_{max}:** 2988, 2901, 1453, 1326, 1102, 1066, 833,812, 667; **HRMS** (ESI^-) m/z found $[M-H]^-$ 250.9606, $C_{10}H_5BrFN_2$ required 250.9620.

Tert-butyl 5-bromo-3-methyl-1*H*-indazole-1-carboxylate (**28**)

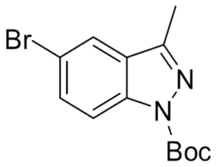

The title compound was prepared according general procedure **2** with 5-bromo-3-methyl-1*H*-indazole (211 mg, 1.00 mmol), CH_2Cl_2 (2.00 mL), Et_3N (132 µL, 1.30 mmol), DMAP (12.2 mg, 0.10 mmol) and di-*tert*-butyl dicarbonate (284 mg, 1.30 mmol). The mixture was purified by flash column chromatography, eluting with a 50-70% gradient of CH_2Cl_2/hexane (v/v) to yield the pure product as a white solid (300 mg, 96%).

R$_f$ = 0.41 (CH_2Cl_2); **Mp** = 120-122 °C; **^{1}H NMR** (500 MHz, $CDCl_3$) δ 8.00 (d, *J* = 8.8 Hz, 1H), 7.79 (dd, *J* = 1.9, 0.6 Hz, 1H), 7.59 (dd, *J* = 8.9, 1.9 Hz, 1H), 2.57 (s, 3H), 1.71 (s, 9H); **^{13}C NMR** (126 MHz, $CDCl_3$) δ 149.2, 147.7, 139.2, 132.0, 127.7, 123.2, 116.3, 85.2, 28.3, 12.4; **IR ν_{max}:** 2980, 1727, 1396, 1369, 1245, 1160, 1049, 1020, 806; **HRMS** (ESI^+) m/z found $[M+Na]^+$ 333.0212, $C_{13}H_{15}BrN_2O_2Na$ required 333.0214.

Data in accordance with the literature.[3]

Tert-butyl 5-bromo-3-(bromomethyl)-1*H*-indazole-1-carboxylate (**29**)

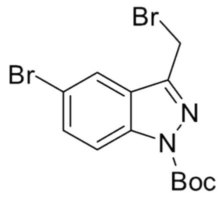

Indazole **28** (249 mg, 0.80 mmol) was suspended in CCl_4 (15 mL), NBS (214 mg, 1.20 mmol) was added and the solution was degassed with a stream of nitrogen for 15 min. AIBN (87.0 mg, 0.53 mmol) was then added and the solution stirred at reflux for 18 h. The mixture was then cooled to rt and the solvent removed *in vacuo*. The residue was purified by flash column chromatography, eluting with a 2-10% gradient of EtOAc/hexane (v/v) to yield the title compound as a white gum (230 mg, 74%).

R$_f$ = 0.48 (10% EtOAc/hexane); **^{1}H NMR** (500 MHz, $CDCl_3$) δ 8.03 (d, *J* = 8.9 Hz, 1H), 8.00 (dd, *J* = 1.9, 0.6 Hz, 1H), 7.63 (dd, *J* = 8.9, 1.9 Hz, 1H), 4.74 (s, 2H), 1.72 (s, 9H); **^{13}C NMR** (126 MHz, $CDCl_3$) δ 148.7, 146.4, 139.7, 132.5, 125.8, 123.5, 117.2, 116.5, 86.0, 28.3, 22.6; **IR ν$_{max}$:** 1737, 1343, 1277, 1152, 1090, 8445; **HRMS** (ESI$^+$) m/z found $[M+Na]^+$ 410.9317, $C_{13}H_{14}Br_2N_2O_2Na$ required 410.9320.

2,6-Dichloro-4-formylphenyl trifluoromethansulfonate

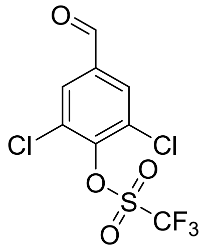

The compound was synthesised as previously described.[4]

To a solution of 3,5-dichloro-4-hydroxybenzaldehyde (200 mg, 1.05 mmol, 1.0 equiv) in anhydrous CH_2Cl_2 (1.50 mL) was added anhydrous pyridine (0.15 mL, 1.88 mmol, 1.6 equiv). The solution was cooled to 0 °C and trifluoromethanesulfonic anhydride (0.26 mL, 1.52 mmol, 1.4 equiv) added dropwise over 30 min. The reaction was allowed to warm to rt and stirred overnight. The volatiles were removed *in vacuo* and the residue was diluted with H_2O and extracted with EtOAc (2 x 5 mL). The organic layer was washed with 10% aqueous HCl, 5% aqueous Na_2CO_3, a saturated aqueous solution of NaCl and H_2O, then dried ($MgSO_4$), filtered and concentrated *in vacuo*. The crude product was then purified by flash column chromatography, eluting with 10% EtOAc/hexane (v/v) to provide the 2,6-dichloro-4-formylphenyl trifluoromethansulfonate as a colourless oil (191 mg, 0.60 mmol, 57%).

R$_f$ = 0.19 (10% EtOAc/Hexane); **IR ν$_{max}$**: 1706, 1429, 1210, 1129, 855, 744, 709; **^{1}H NMR** (500 MHz, $CDCl_3$): δ 9.97 (1H, s), 7.97 (2H, s); **^{13}C NMR** (126 MHz, DMSO-*d$_6$*): δ 190.1, 141.1, 137.2, 131.0, 117.0 (q, *J* = 320 Hz, 1C); **^{19}F NMR** (376 MHz, $CDCl_3$): δ -71.4.

Data in accordance with the literature.[4]

(2,6-dichloro-2'-ethyl-[1,1'-biphenyl]-4-yl)methanamine TFA salt (αD-binder)

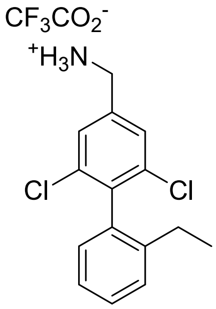

The compound was synthesised as previously described.[4]

A mixture of 2,6-dichloro-4-formylphenyl trifluoromethansulfonate (771 mg, 2.40 mmol, 1.0 equiv), 2-ethyl-phenylboronic acid (430 mg, 2.87 mmol, 1.2 equiv), $PdCl_2$(dppf)·CH_2Cl_2 (98 mg, 0.12 mmol, 0.05 equiv) and K_3PO_4 (1.02 g, 4.80 mmol, 2.0 equiv) were solvated with DME (5.60 mL), EtOH (0.35 mL) and H_2O (0.06 mL). The reaction mixture was degassed by bubbling nitrogen through the solution for 15 min and then heated to reflux for 3 h. The reaction was allowed to cool to rt, filtered through Celite®, washed with Et_2O, and the solvent removed *in vacuo*. A solution of crude 2,6-dichloro-2'-ethyl-[1,1'-biphenyl]-4-carbaldehyde (200 mg), *tert*-butylcarbamate (170 mg, 4.30 mmol), EtSiH (0.66 mL, 4.30 mmol) and TFA (0.21 mL, 2.72 mmol) in MeCN (3.20 mL) was stirred at rt for 14 h. The mixture was diluted with Et_2O and washed with an aqueous solution of $NaHCO_3$ and a saturated aqueous solution of NaCl. The combined organic layers were dried ($MgSO_4$), filtered and concentrated *in vacuo*. To the *N*-Boc derivative was added TFA (4.5 mL) and the mixture stirred at room temperature, with monitoring by TLC. After 15 min, the excess TFA was blown off under nitrogen and the residue stirred in Et_2O to give (2,6-dichloro-2'-ethyl-[1,1'-biphenyl]-4-yl)methanamine TFA salt as a white solid (24 mg, 0.09 mmol).

R_f = 0.05 (amine) (10% MeOH/CH_2Cl_2); **Mp** 218-222 °C; **IR ν_{max}**: 3373, 1674, 1118, 970; **^{1}H NMR** (500 MHz, MeOD): δ 7.66 (2H, s), 7.41-7.39 (2H, m), 7.39-7.27 (1H, m), 6.99 (1H, d, *J* = 7.4 Hz), 4.19 (2H, s), 2.36 (2H, q, *J* = 7.6 Hz), 1.08 (2H, t, *J* = 7.6 Hz); **^{13}C NMR** (126 MHz, MeOD): δ 141.6, 135.6, 135.4, 135.1, 128.9, 128.7, 128.3, 128.2, 125.8, 125.7, 41.7, 25.9, 13.9; **^{19}F NMR** (376 MHz, MeOD): δ -76.4.

Data in accordance with the literature.[4]

2.2 NMR spectra

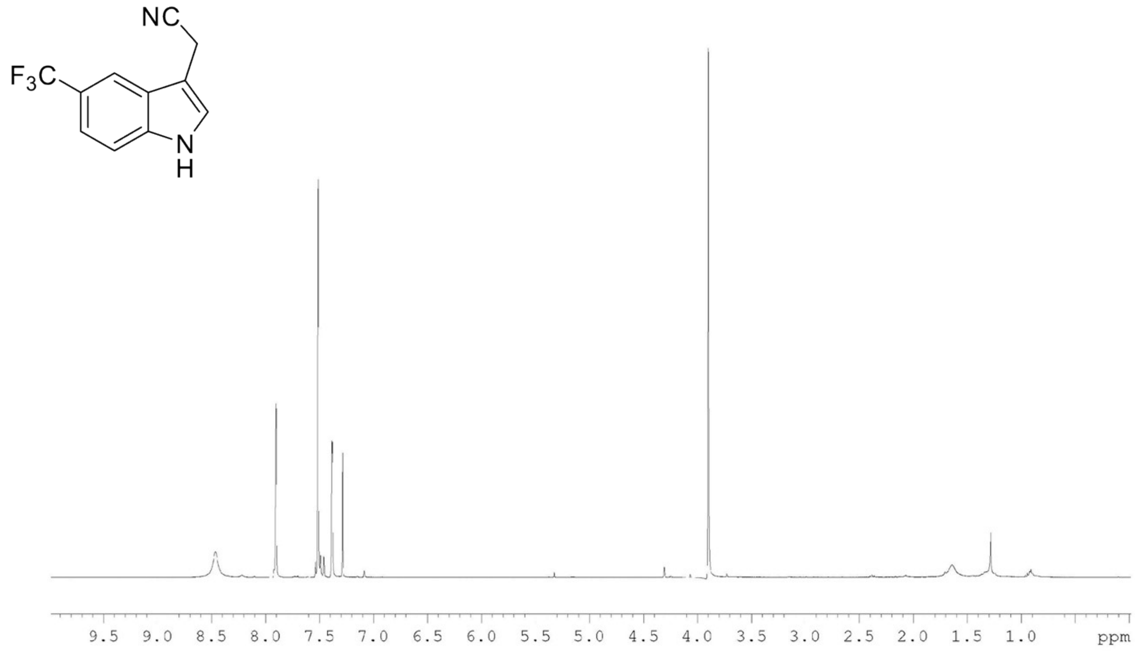

400 MHz, $CDCl_3$, ^1H NMR spectrum

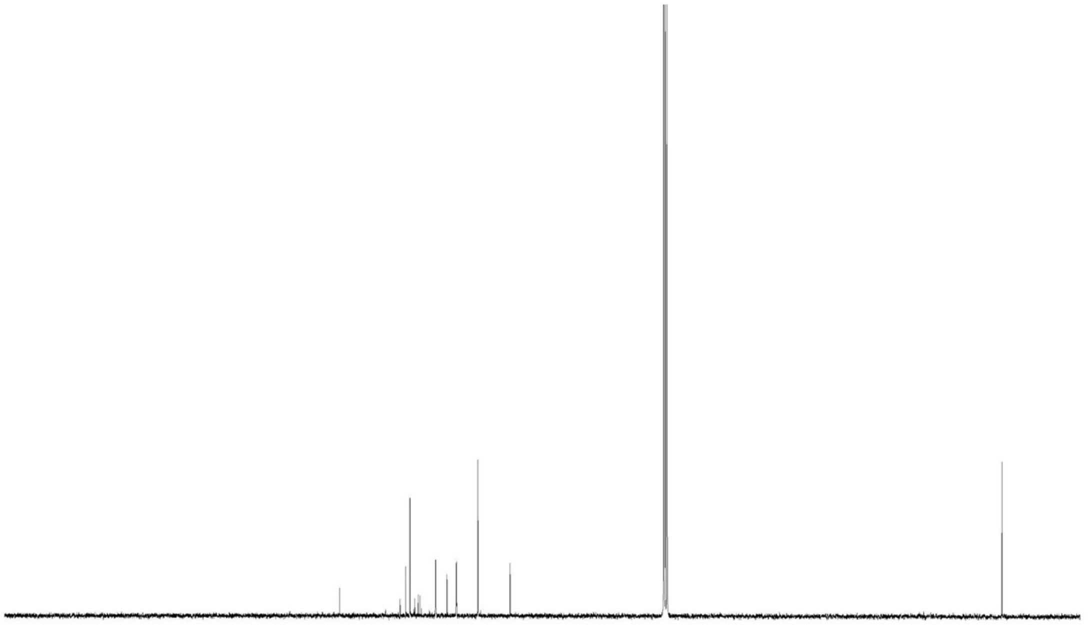

101 MHz, $CDCl_3$, ^{13}C NMR spectrum

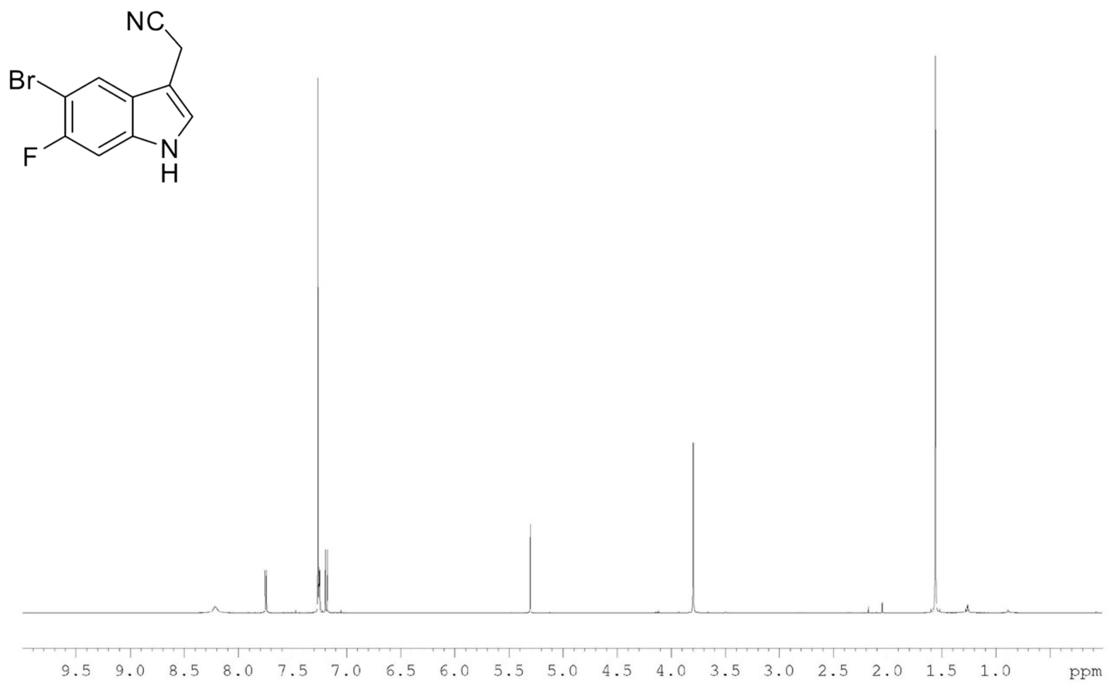

500 MHz, $CDCl_3$, ^1H NMR spectrum

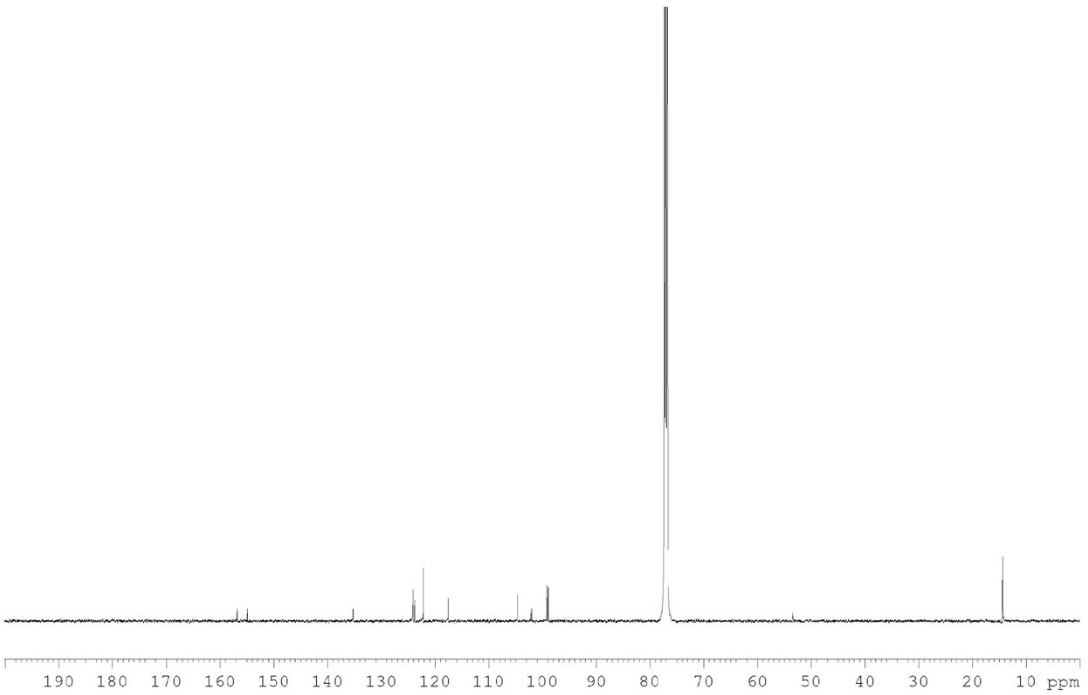

126 MHz, $CDCl_3$, ^{13}C NMR spectrum

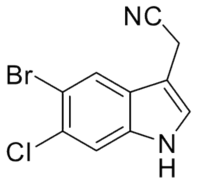

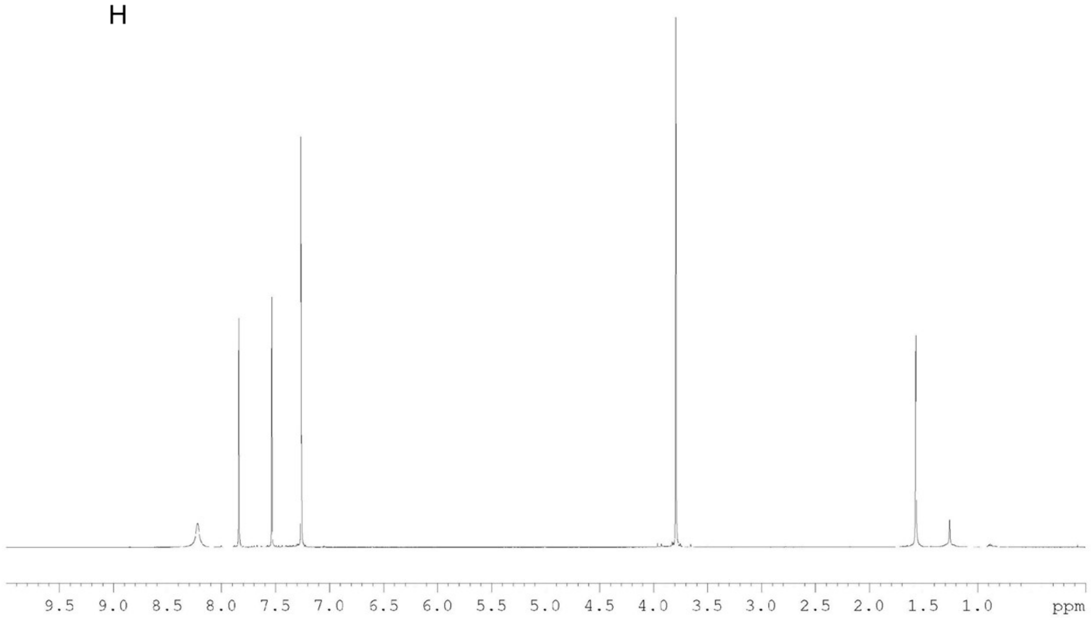

500 MHz, $CDCl_3$, ^{1}H NMR spectrum

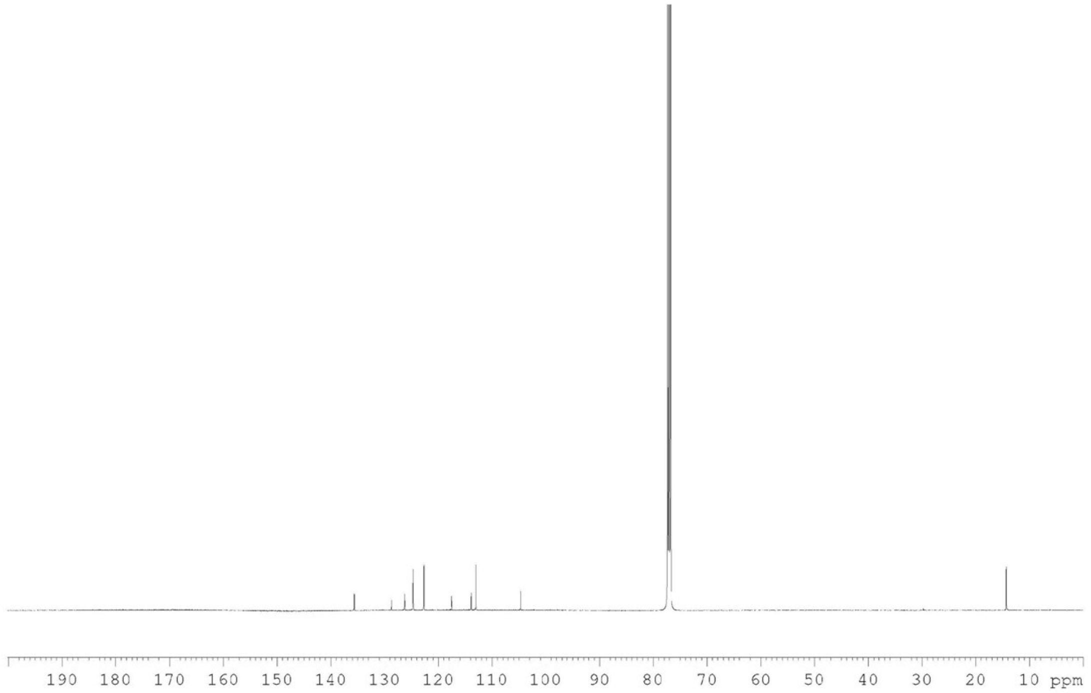

126 MHz, $CDCl_3$, ^{13}C NMR spectrum

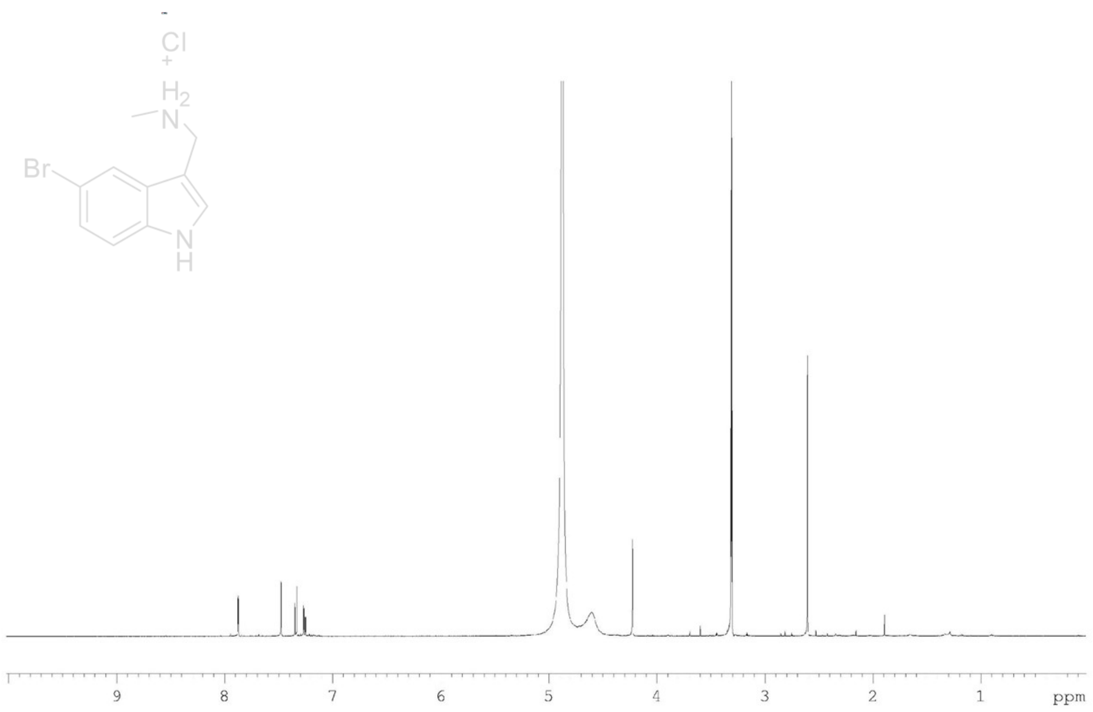

500 MHz, MeOD, ^{1}H NMR spectrum

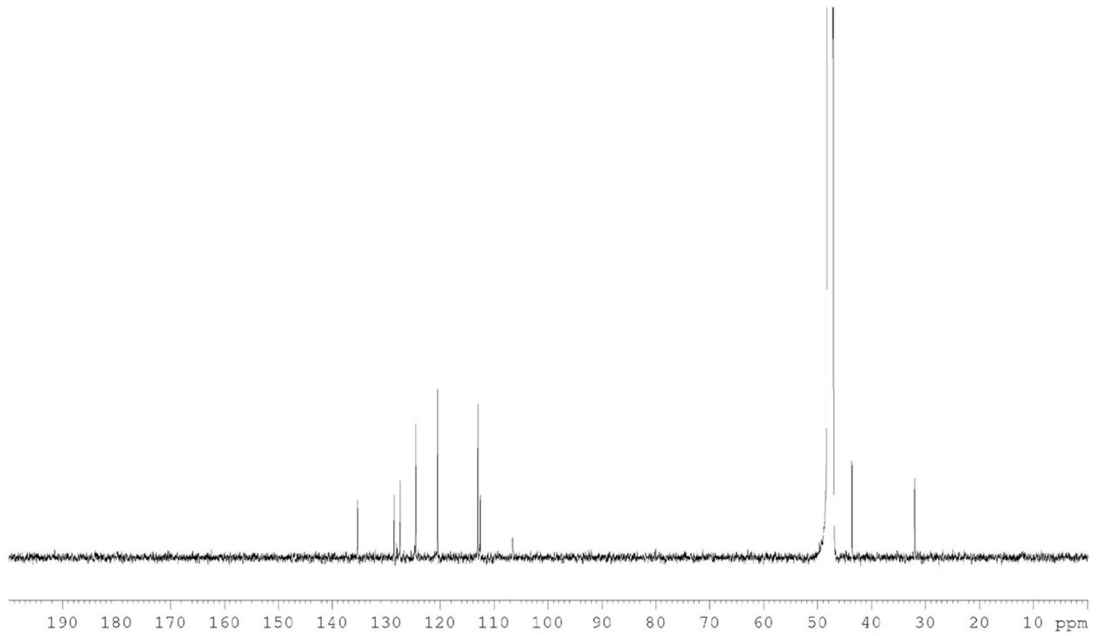

126 MHz, MeOD, ^{13}C NMR spectrum

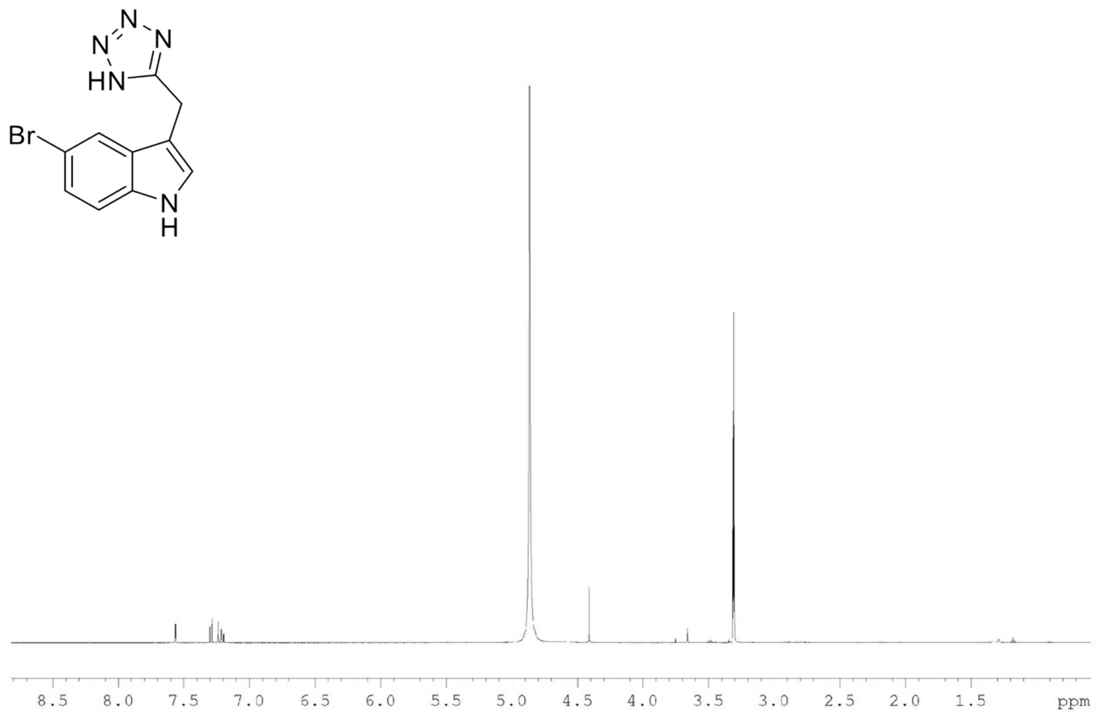

500 MHz, MeOD, ^{1}H NMR spectrum

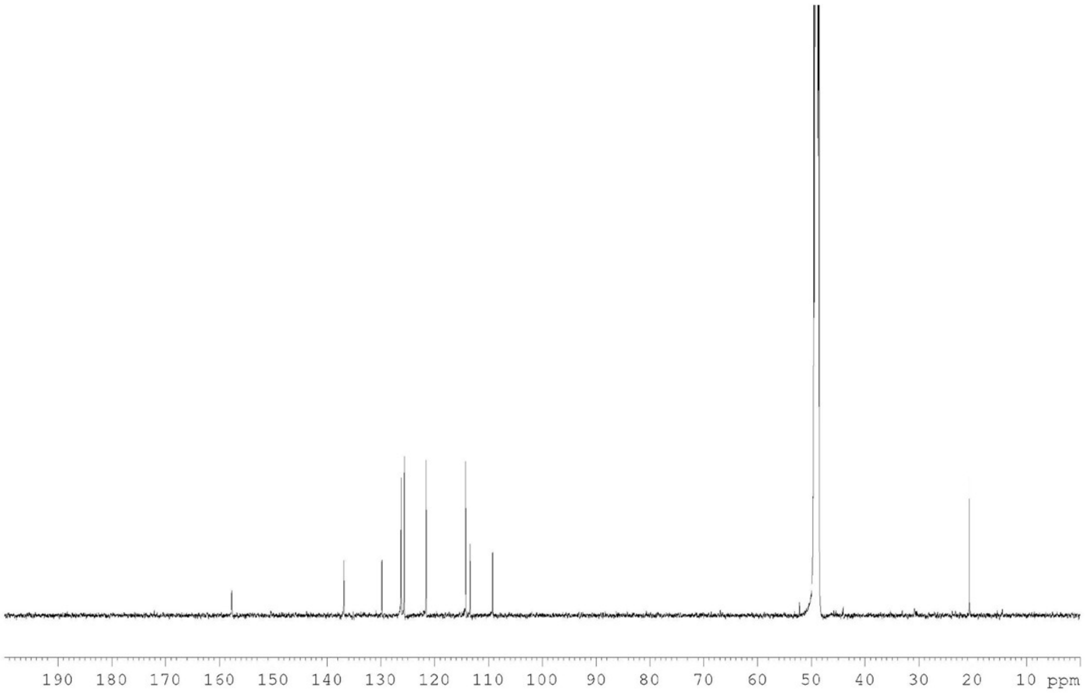

126 MHz, MeOD, ^{13}C NMR spectrum

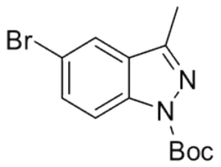

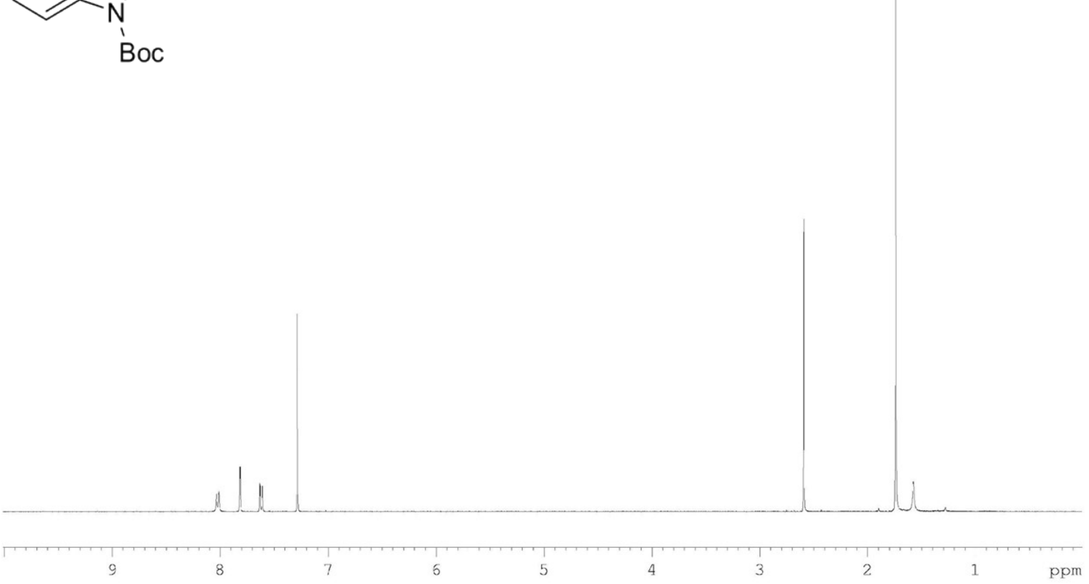

500 MHz, $CDCl_3$, ^1H NMR spectrum

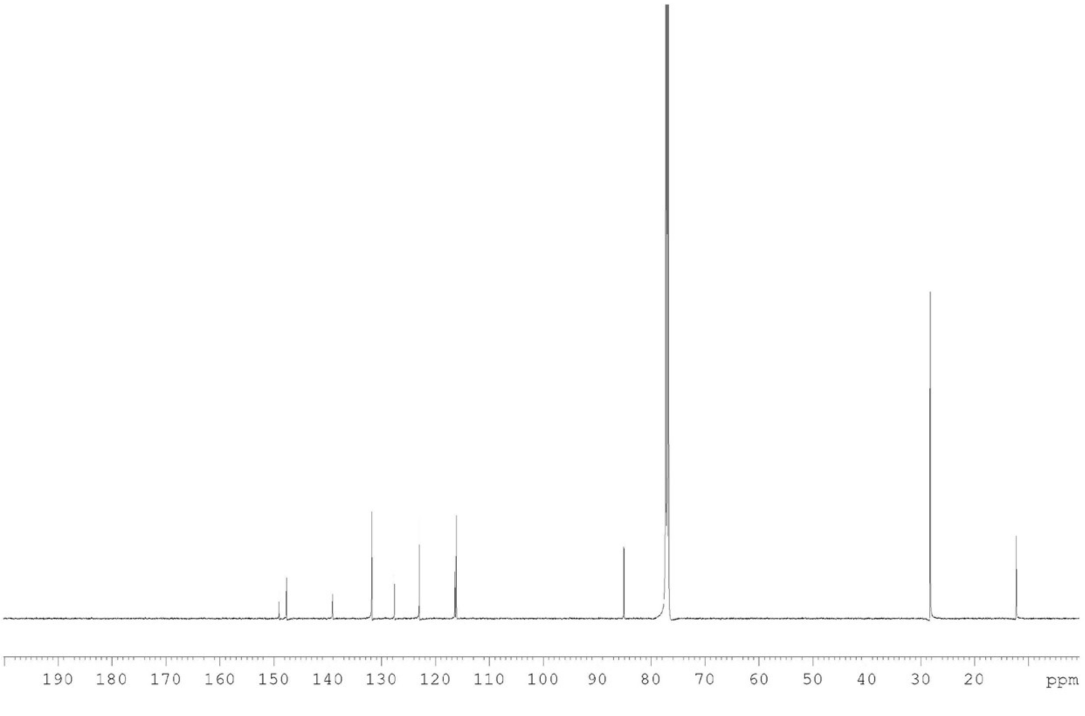

126 MHz, $CDCl_3$, ^{13}C NMR spectrum

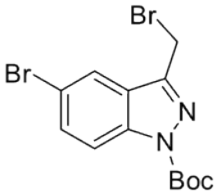

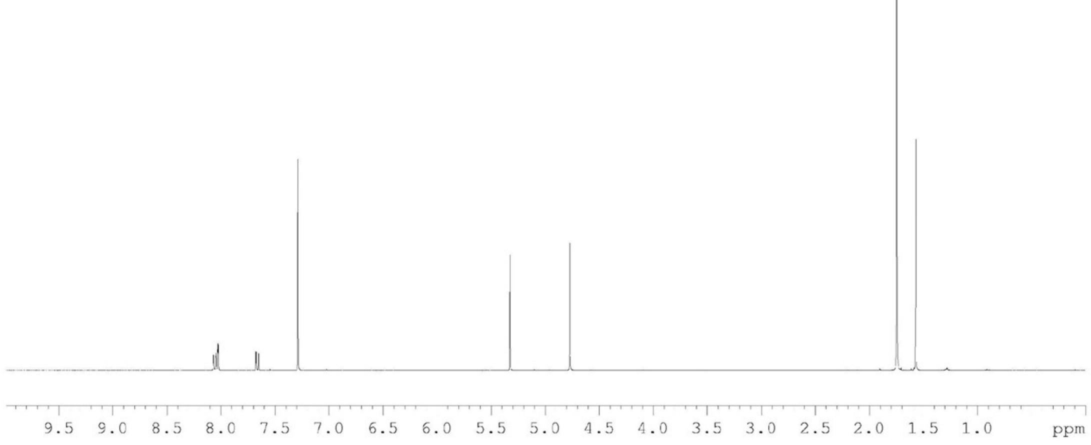

500 MHz, $CDCl_3$, ^1H NMR spectrum

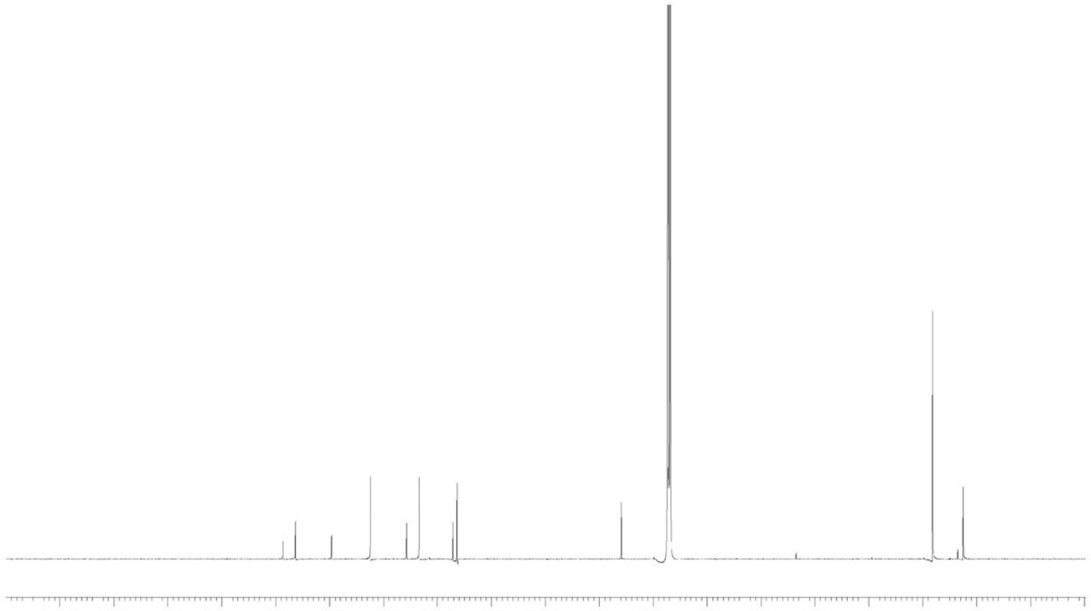

126 MHz, $CDCl_3$, ^{13}C NMR spectrum

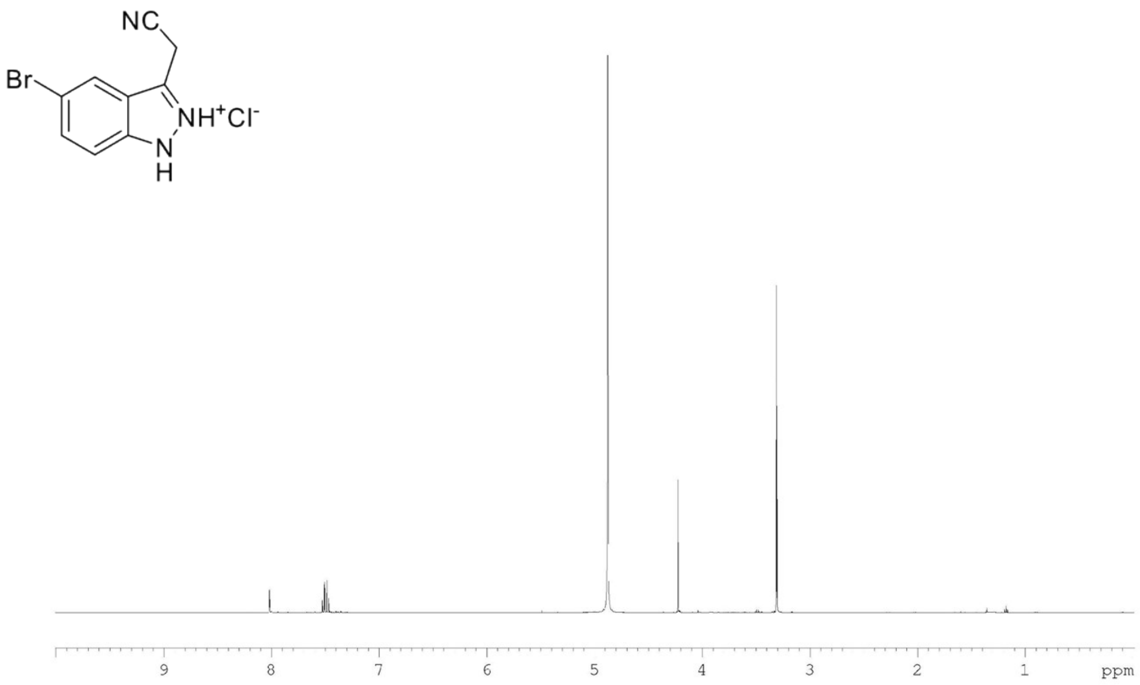

500 MHz, MeOD, ^{1}H NMR spectrum

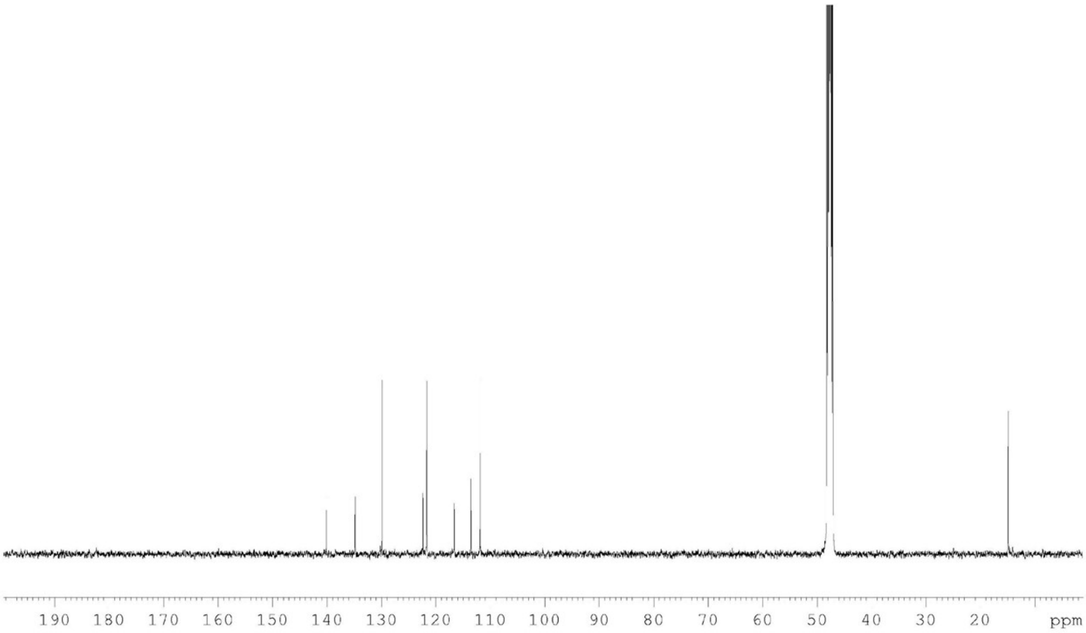

126 MHz, MeOD, ^{13}C NMR spectrum

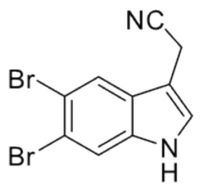

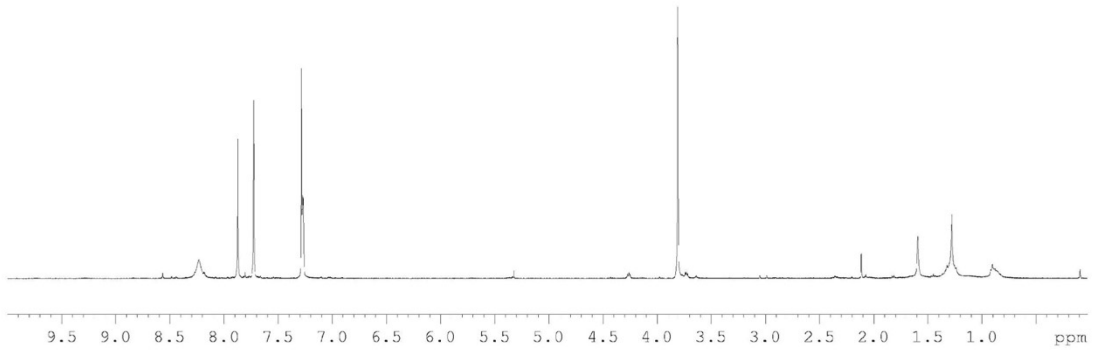

400 MHz, $CDCl_3$, ^{1}H NMR spectrum

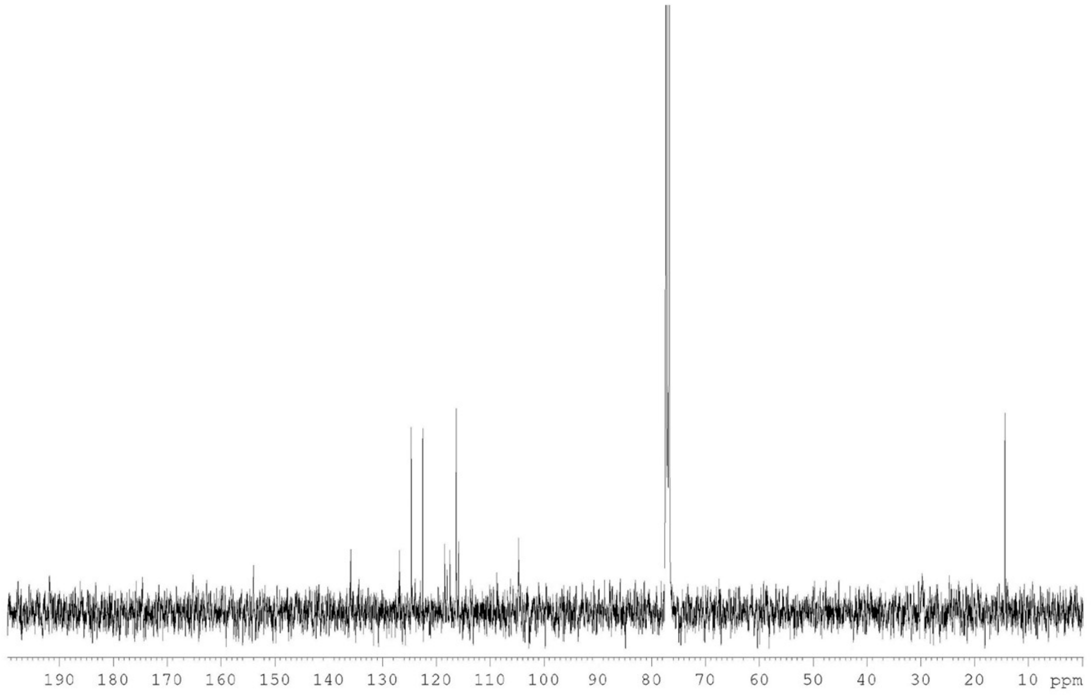

101 MHz, $CDCl_3$, ^{13}C NMR spectrum

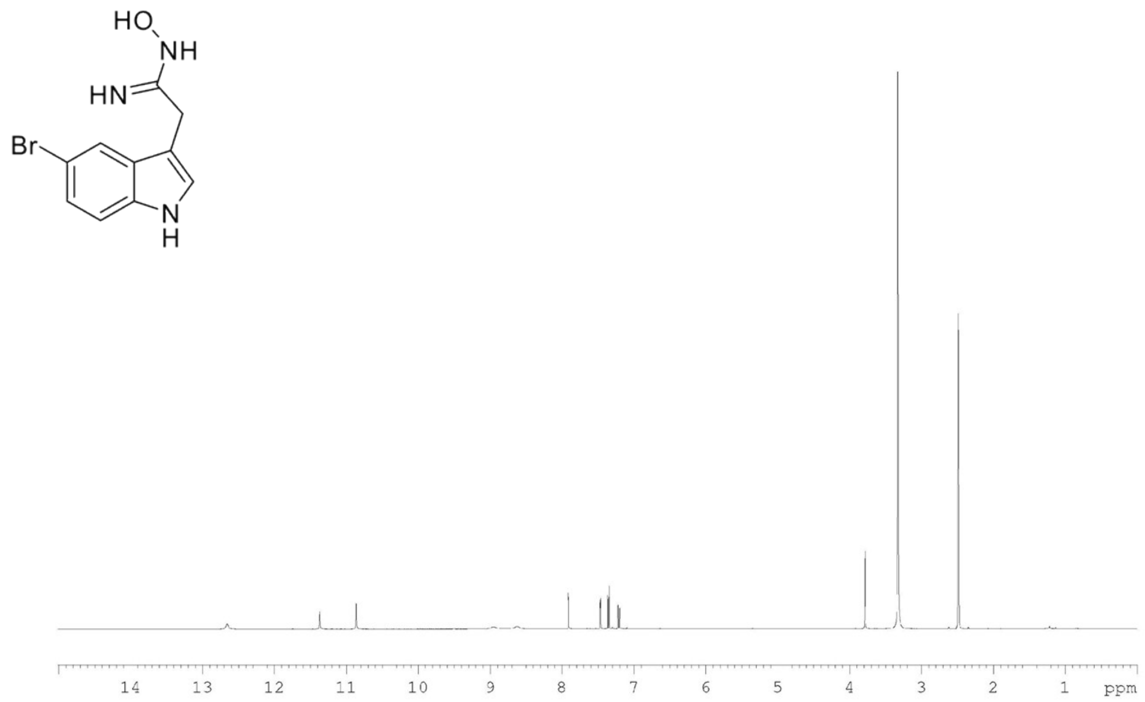

500 MHz, DMSO-d_6, ^{1}H NMR spectrum

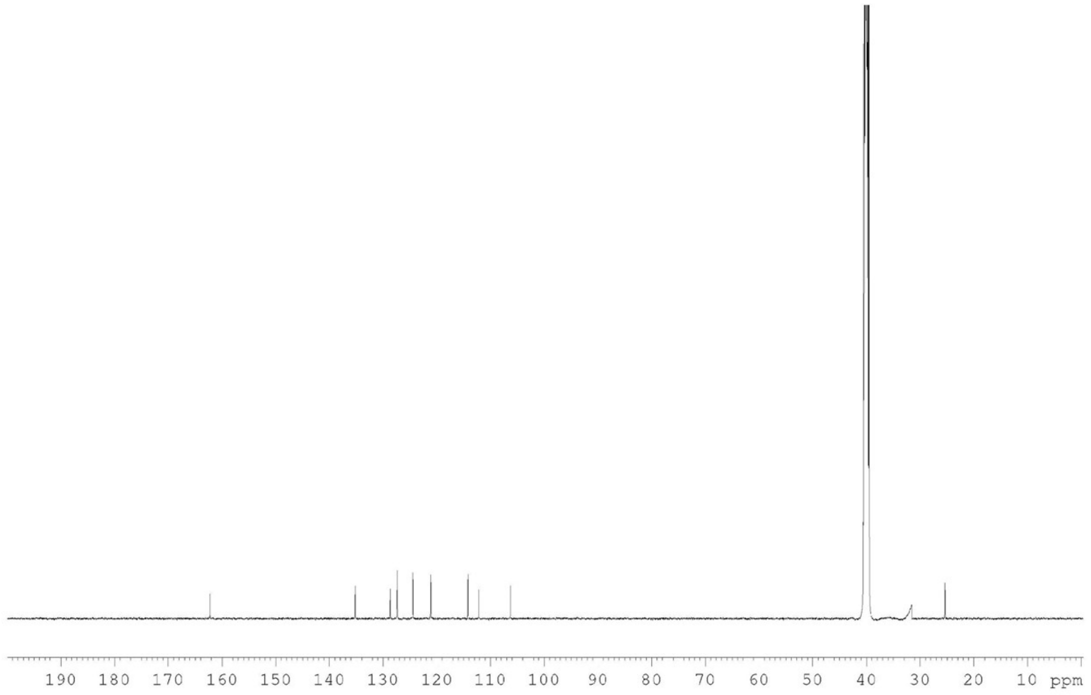

126 MHz, DMSO-d_6, ^{13}C NMR spectrum

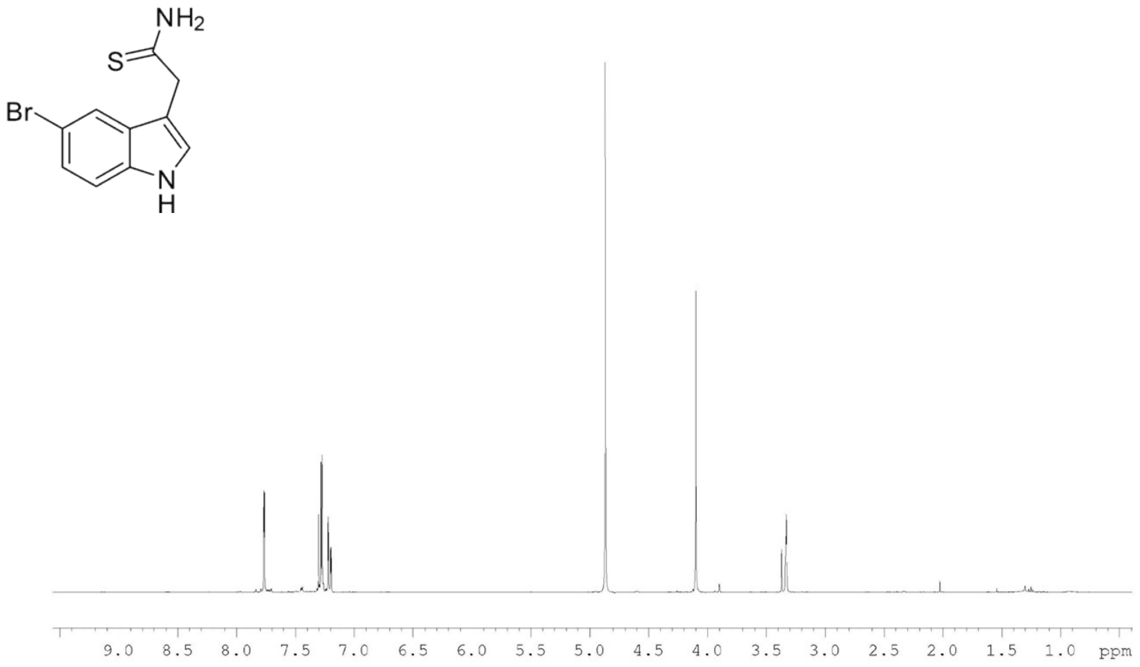

400 MHz, MeOD, ^{1}H NMR spectrum

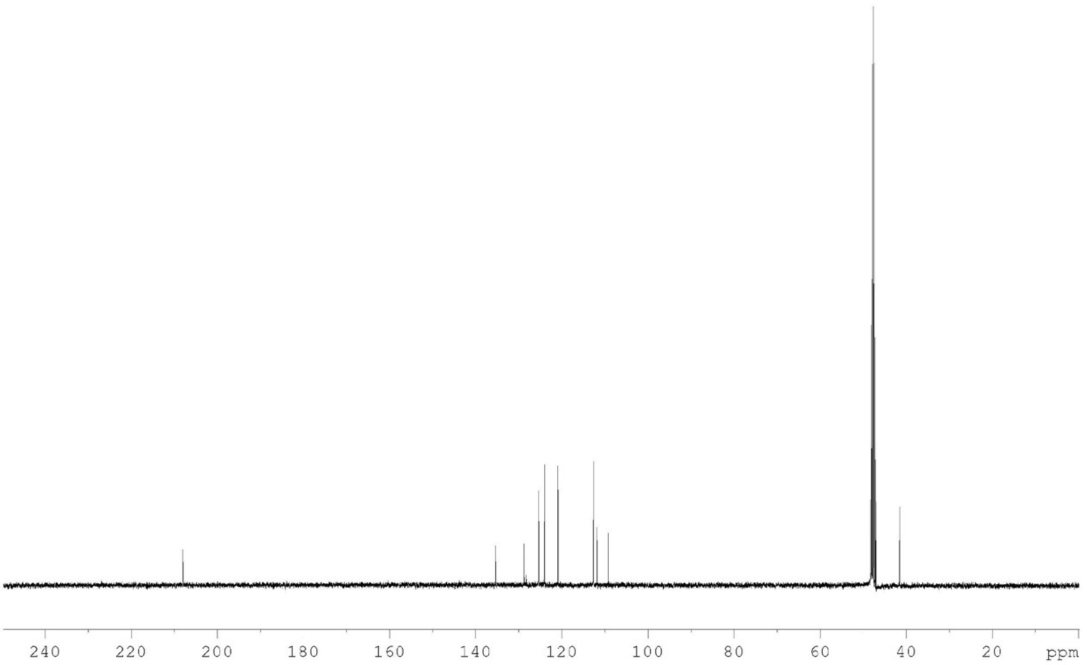

101 MHz, MeOD, ^{13}C NMR spectrum

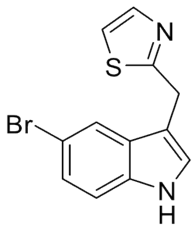

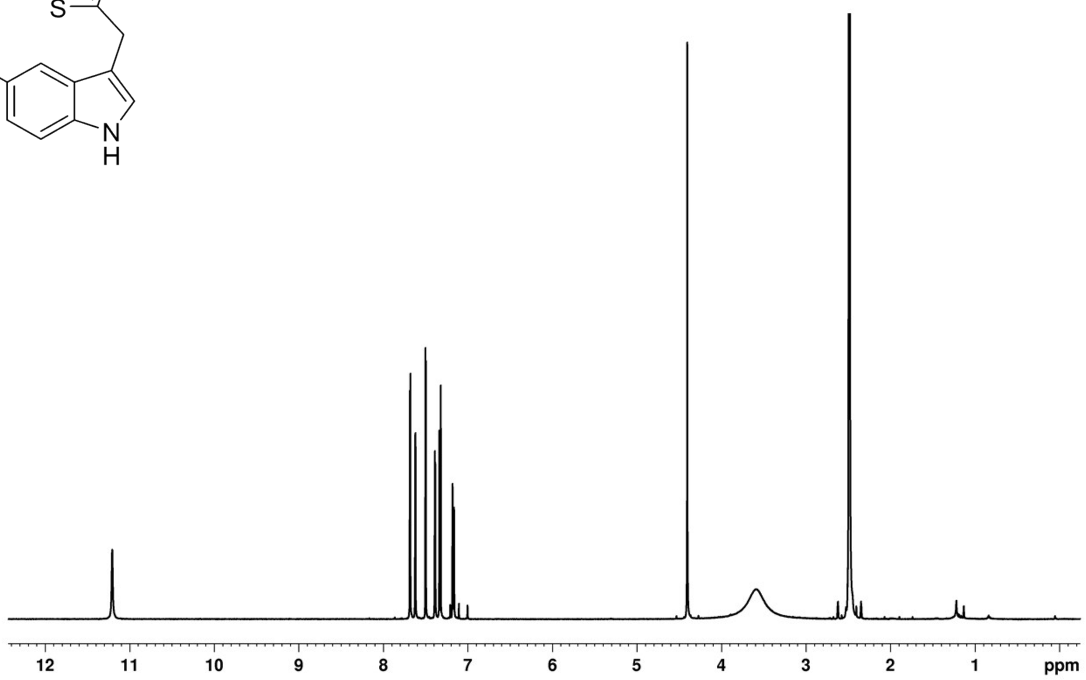

500 MHz, DMSO-d_6, ^{1}H NMR spectrum

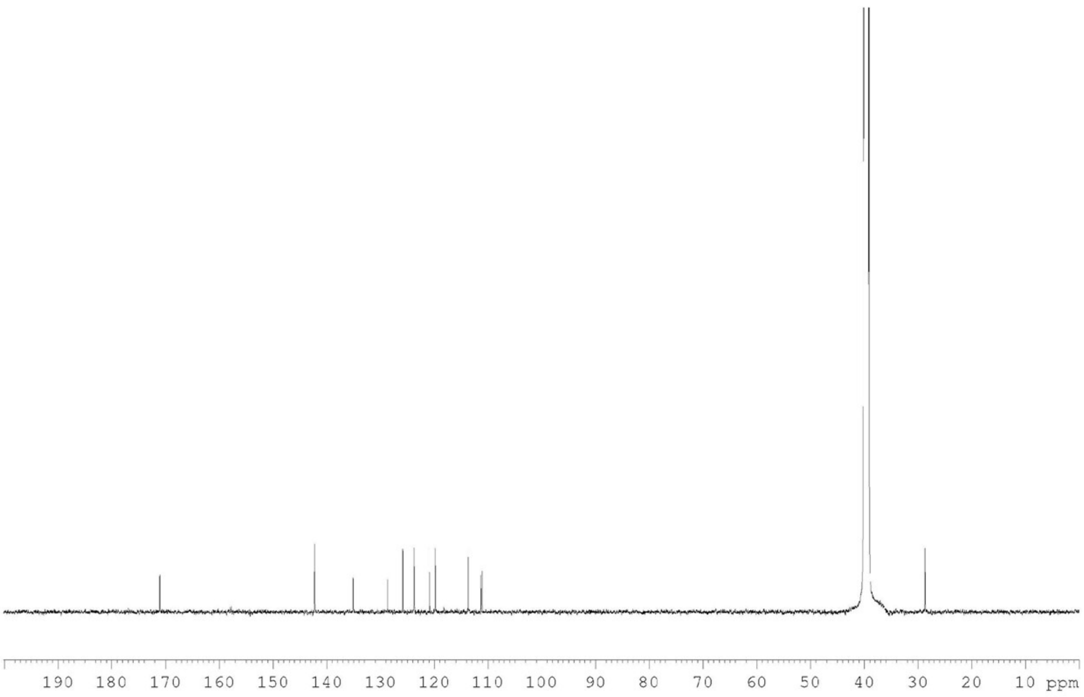

126 MHz, DMSO-d_6, ^{13}C NMR spectrum

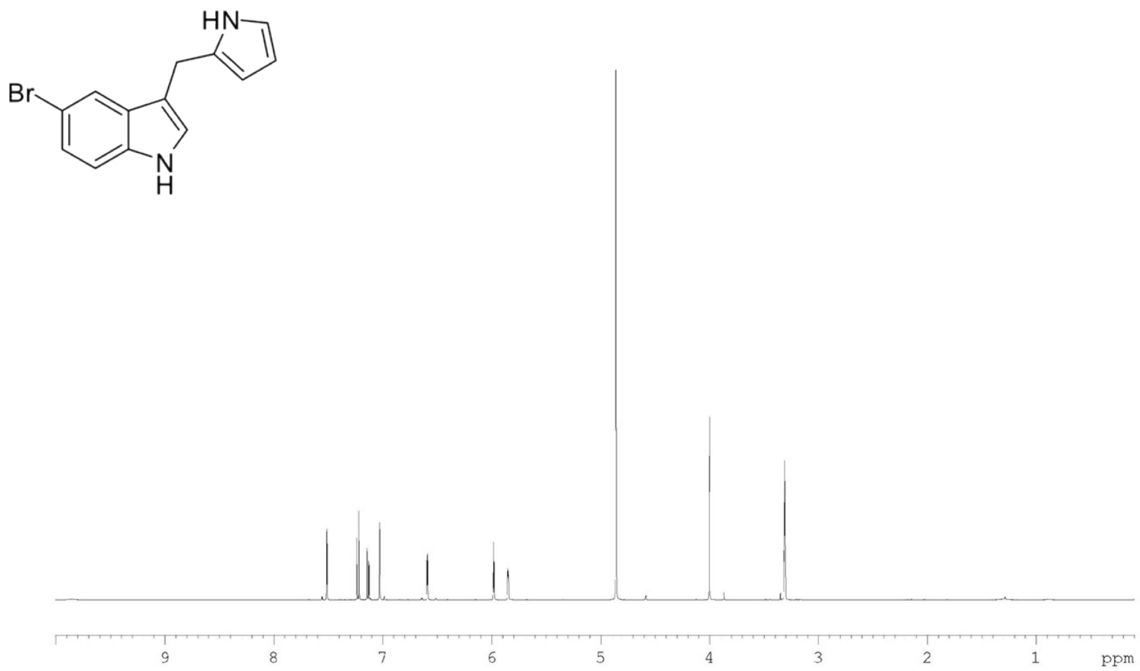

500 MHz, MeOD, ^{1}H NMR spectrum

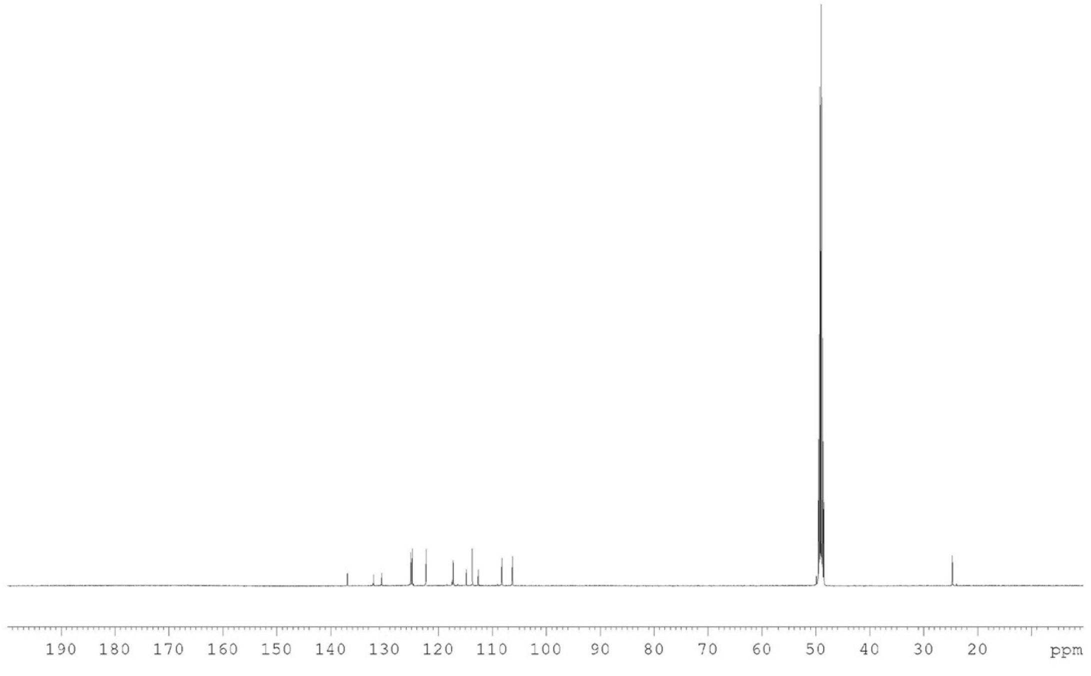

126 MHz, MeOD, ^{13}C NMR spectrum

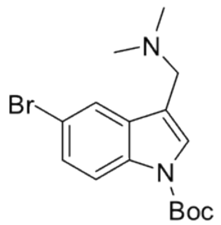

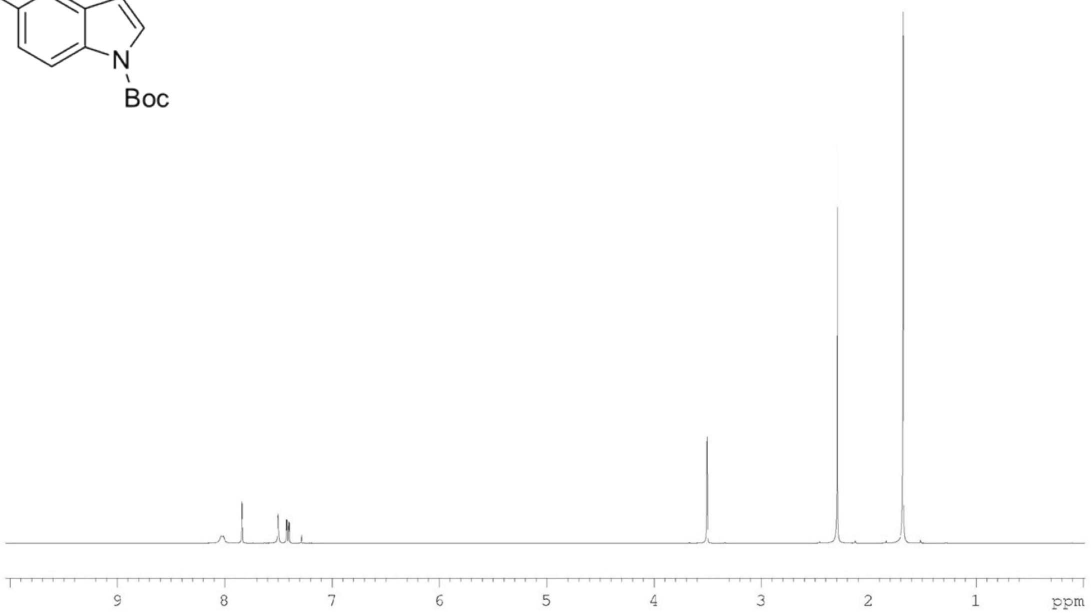

400 MHz, $CDCl_3$, ^1H NMR spectrum

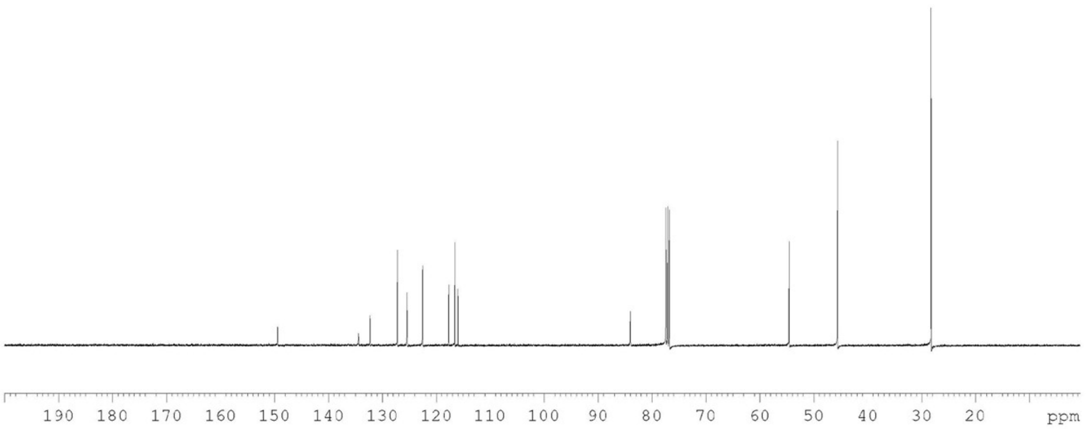

101 MHz, $CDCl_3$, ^{13}C NMR spectrum

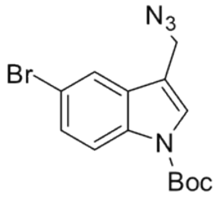

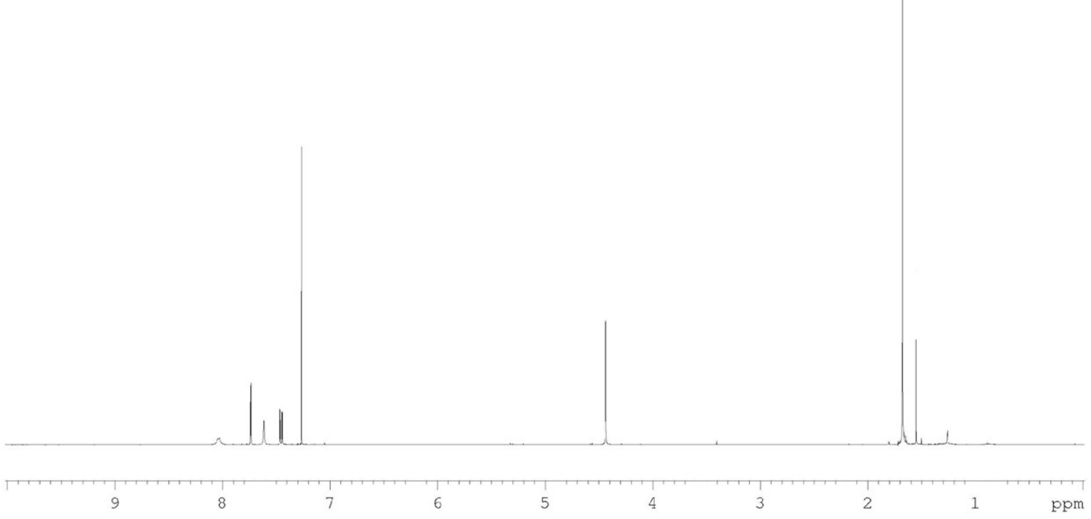

500 MHz, $CDCl_3$, ^{1}H NMR spectrum

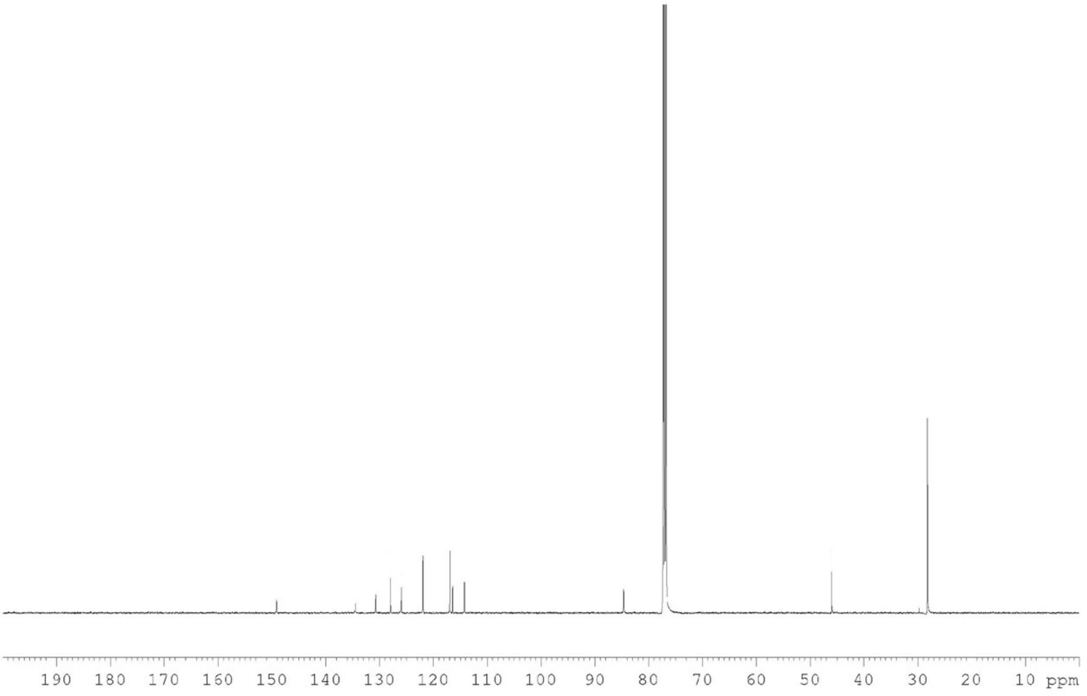

126 MHz, $CDCl_3$, ^{13}C NMR spectrum

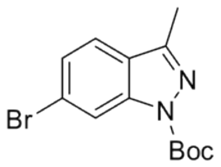

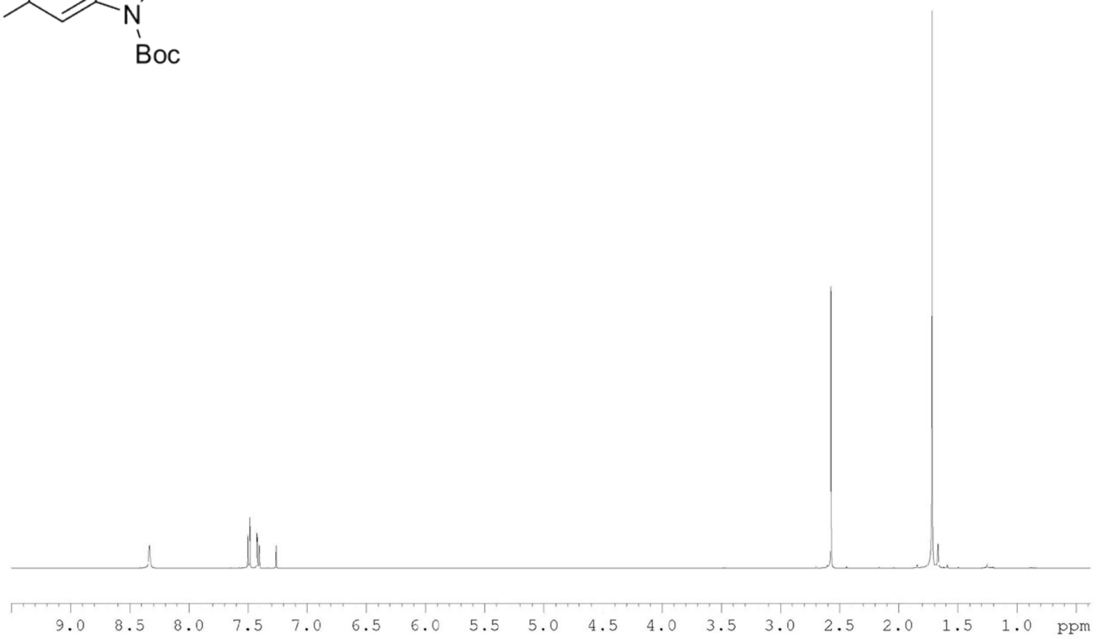

500 MHz, $CDCl_3$, ^1H NMR spectrum

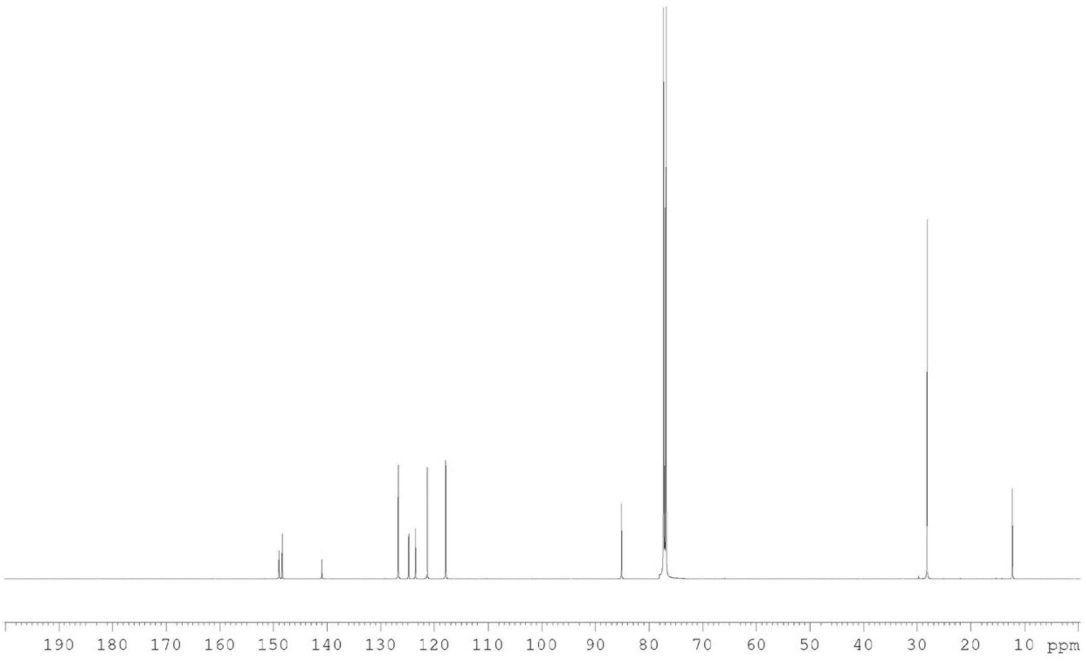

126 MHz, $CDCl_3$, ^{13}C NMR spectrum

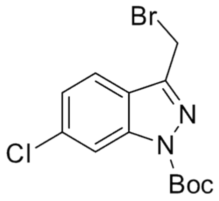

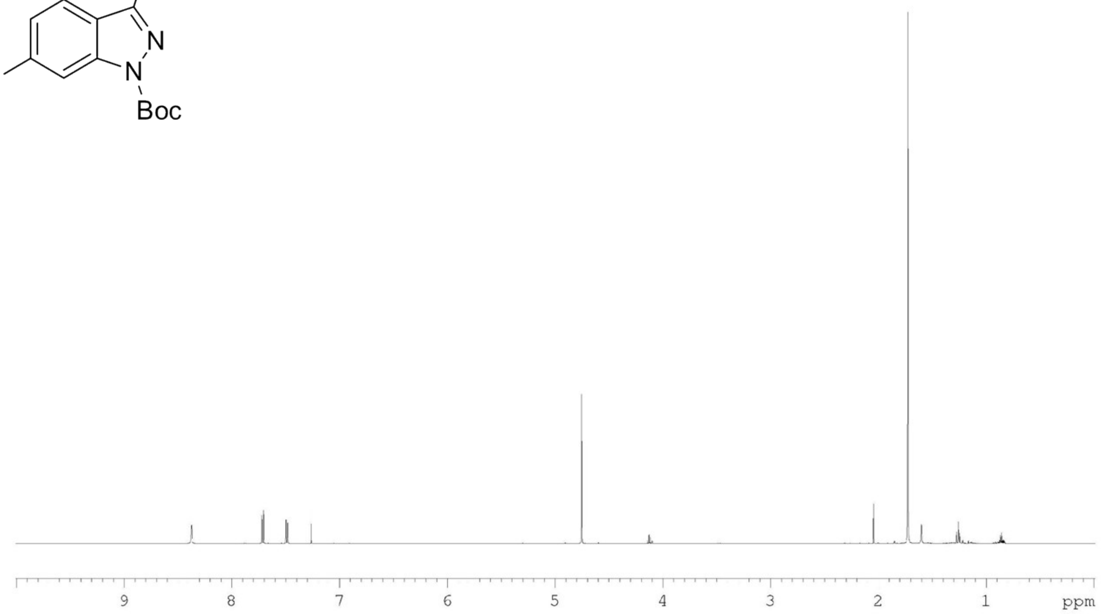

500 MHz, $CDCl_3$, ^1H NMR spectrum

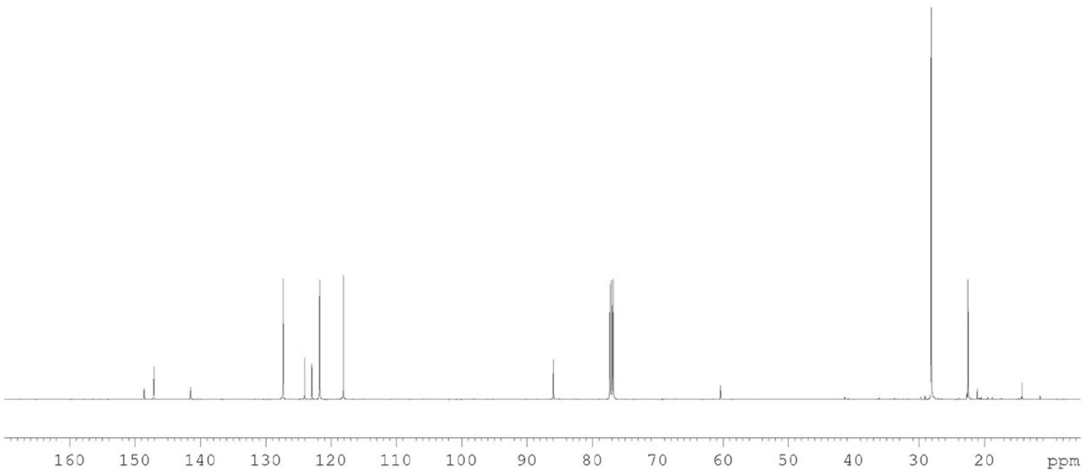

126 MHz, $CDCl_3$, ^{13}C NMR spectrum

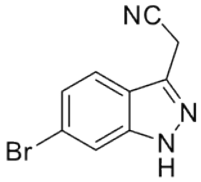

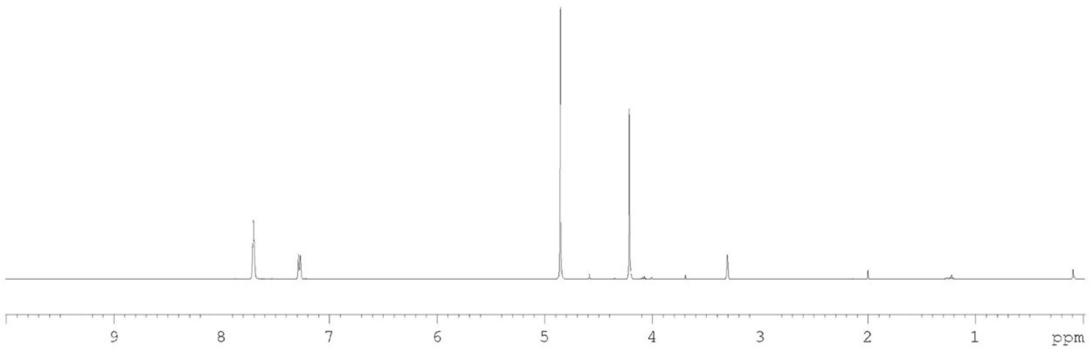

500 MHz, MeOD, ^{1}H NMR spectrum

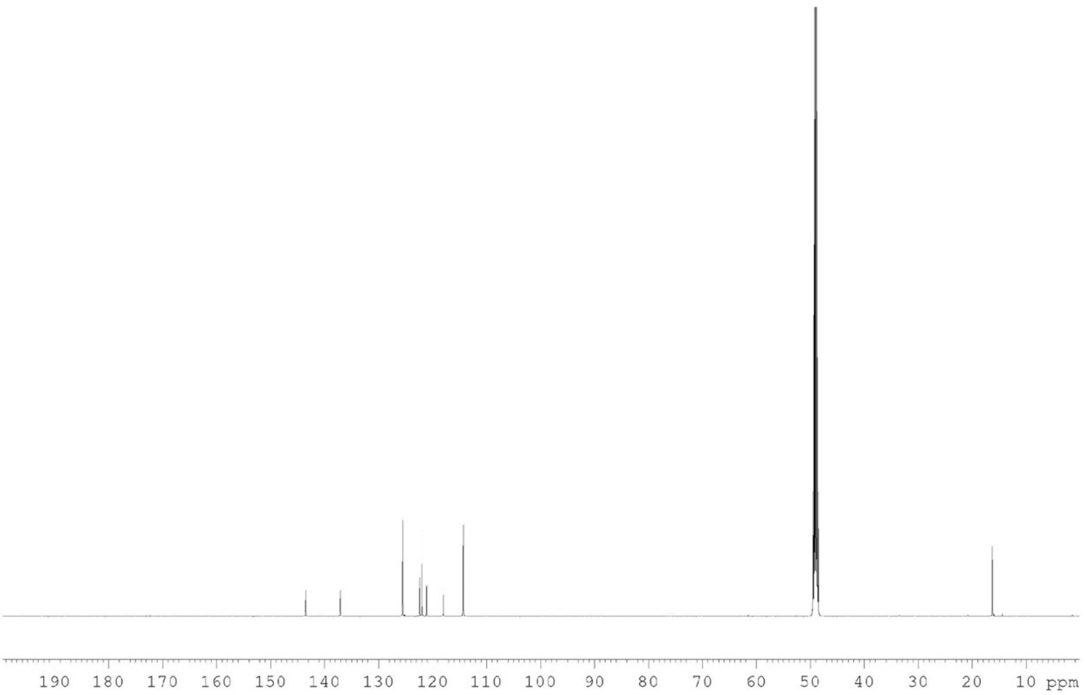

126 MHz, MeOD, ^{13}C NMR spectrum

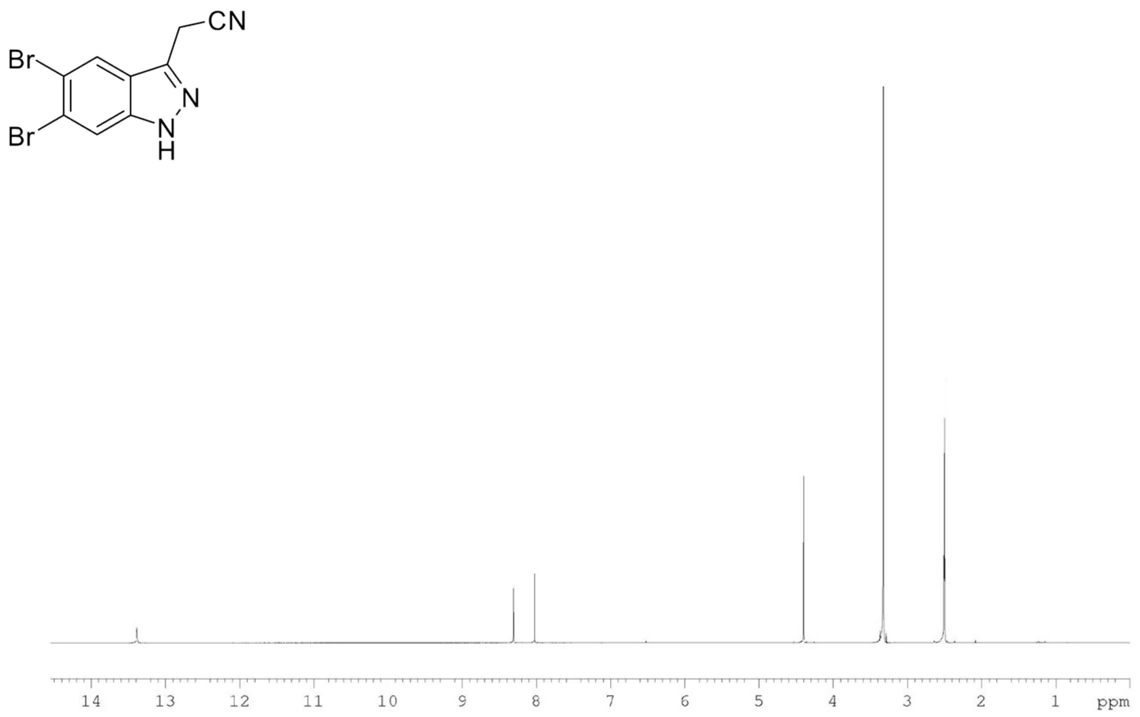

500 MHz, DMSO-d_6, ^{1}H NMR spectrum

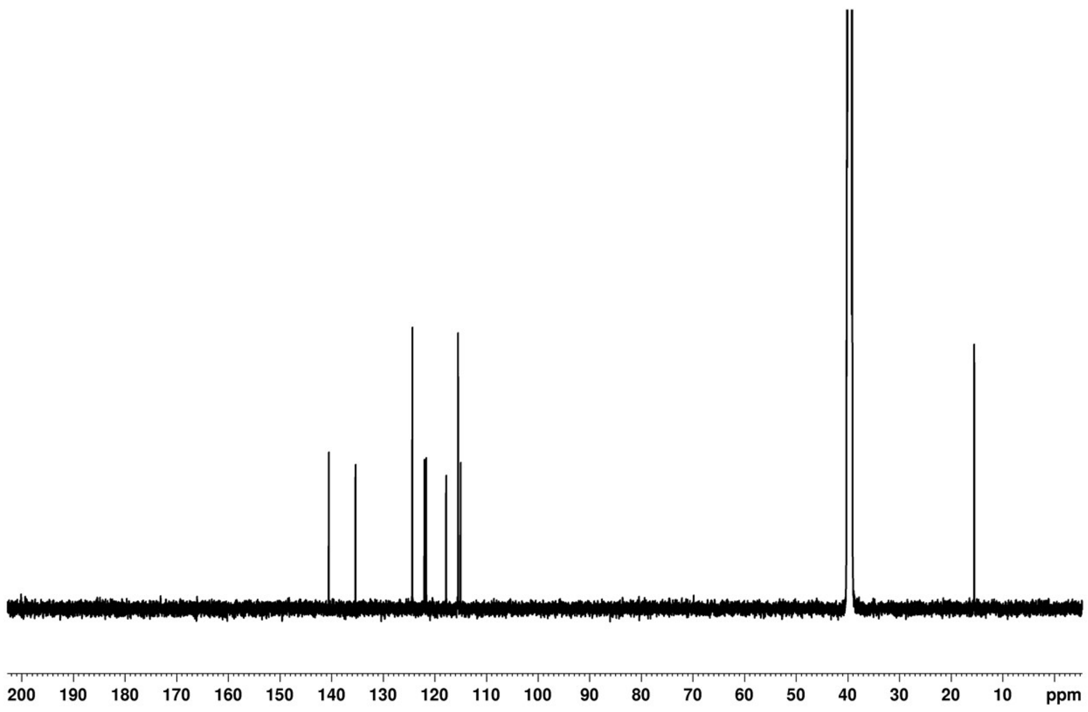

126 MHz, DMSO-d_6, ^{13}C NMR spectrum

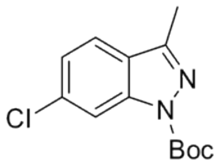

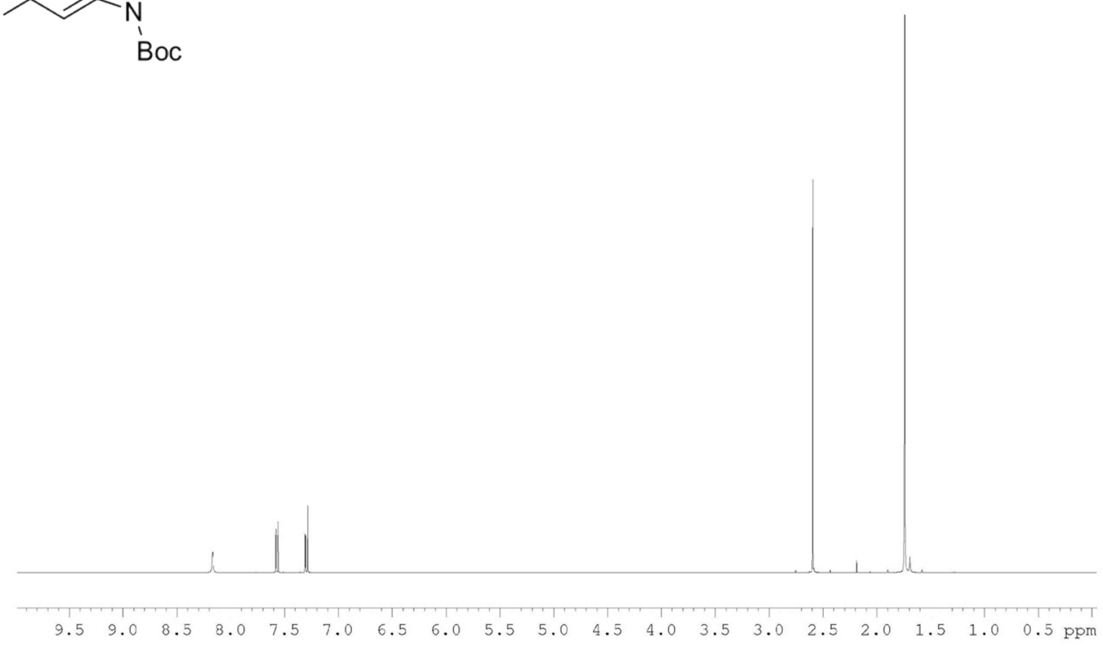

400 MHz, $CDCl_3$, ^{1}H NMR spectrum

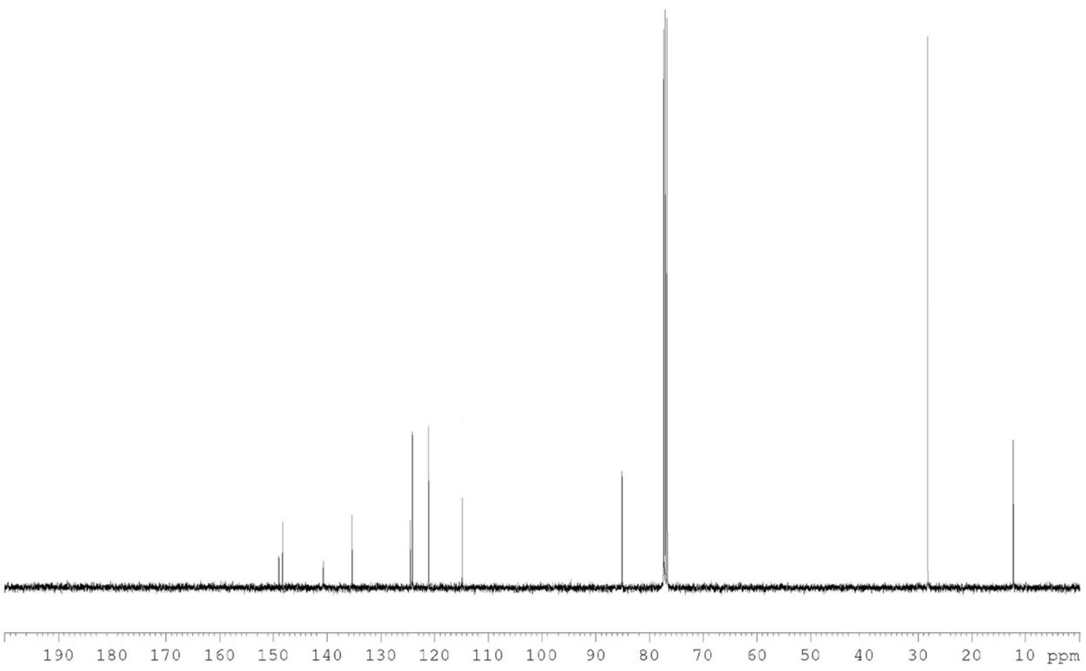

101 MHz, $CDCl_3$, ^{13}C NMR spectrum

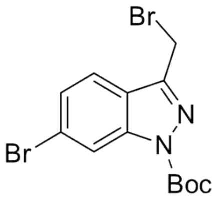

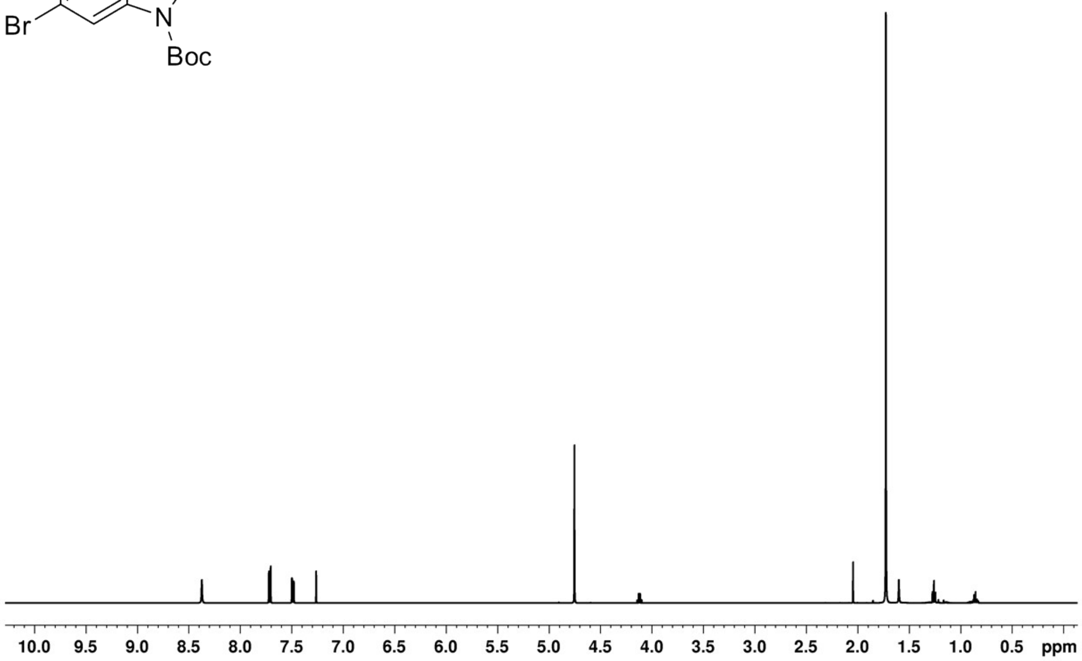

500 MHz, $CDCl_3$, ^1H NMR spectrum

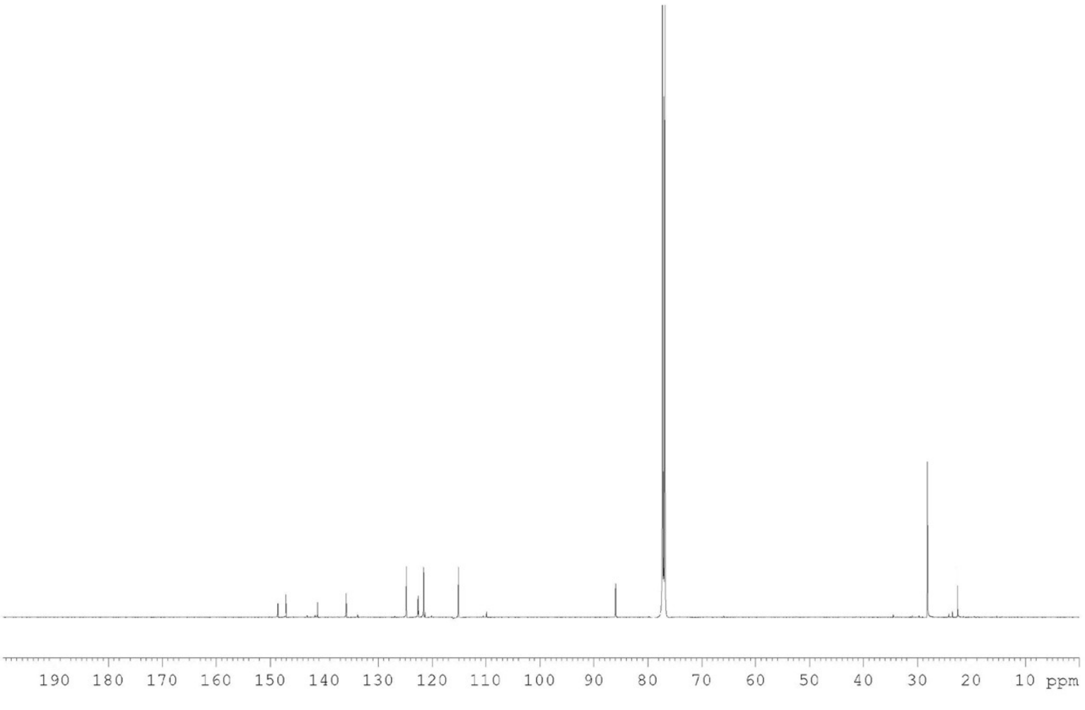

126 MHz, $CDCl_3$, ^{13}C NMR spectrum

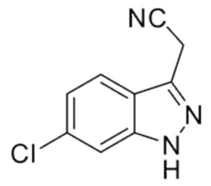

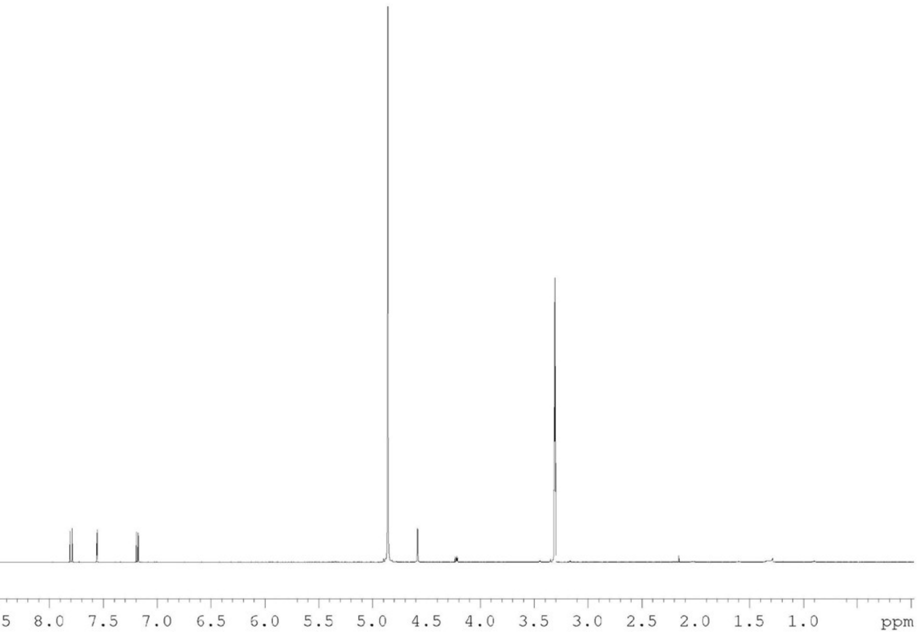

500 MHz, MeOD, ^{1}H NMR spectrum

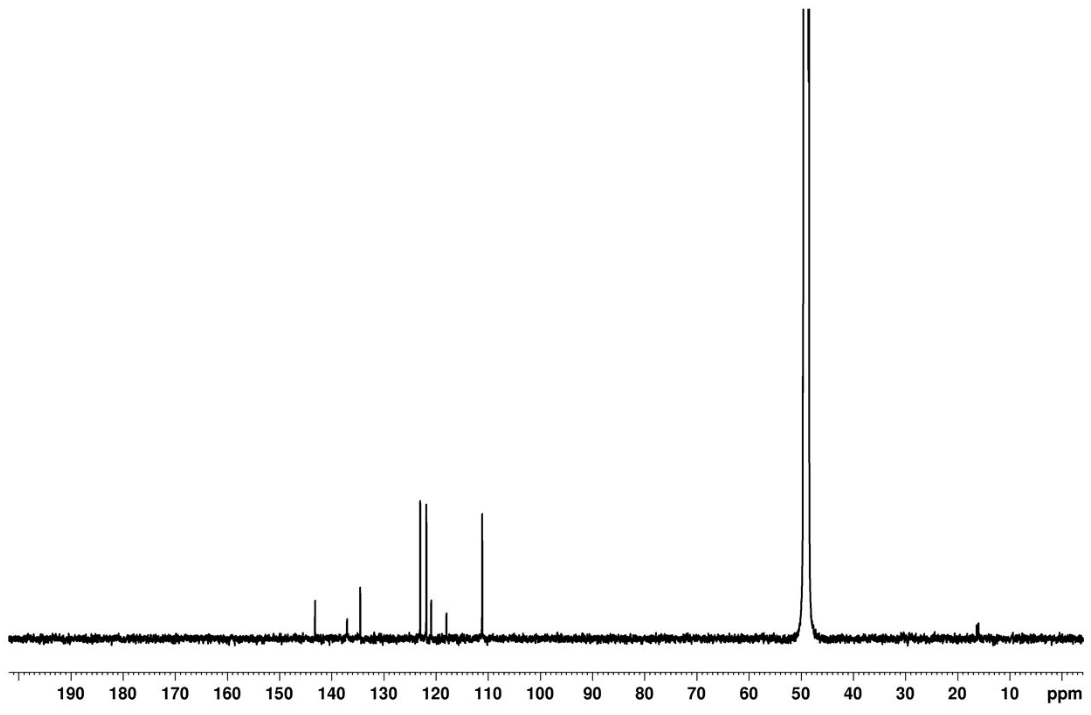

126 MHz, MeOD, ^{13}C NMR spectrum

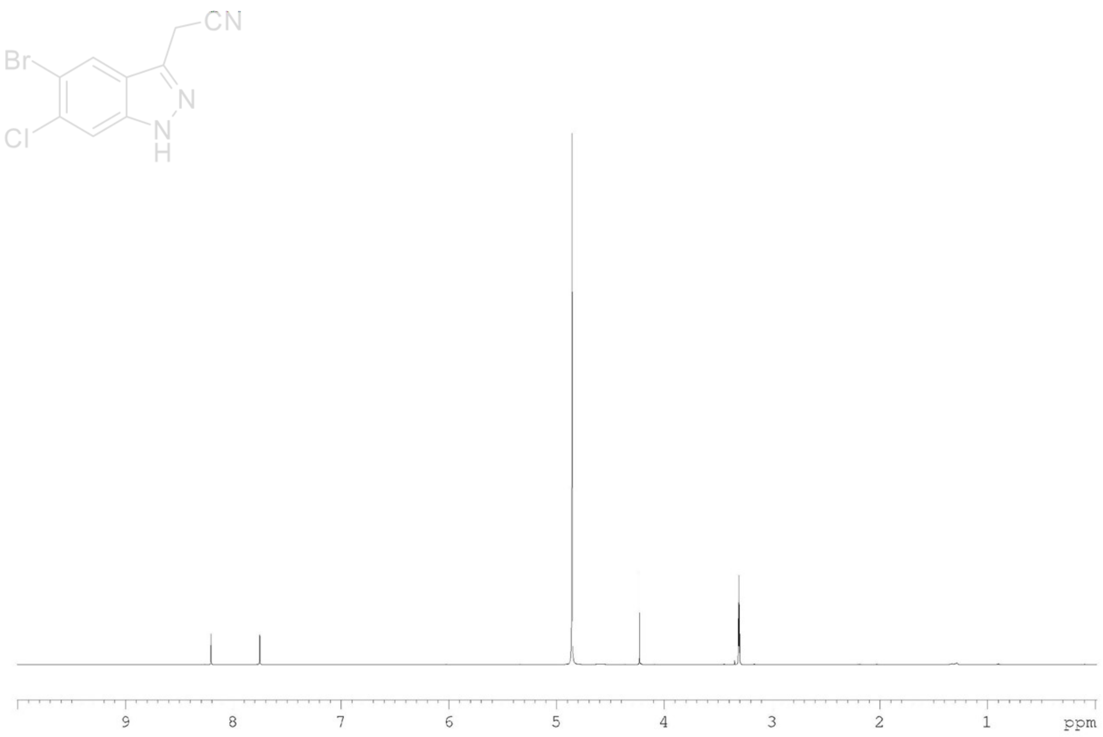

500 MHz, MeOD, ^{1}H NMR spectrum

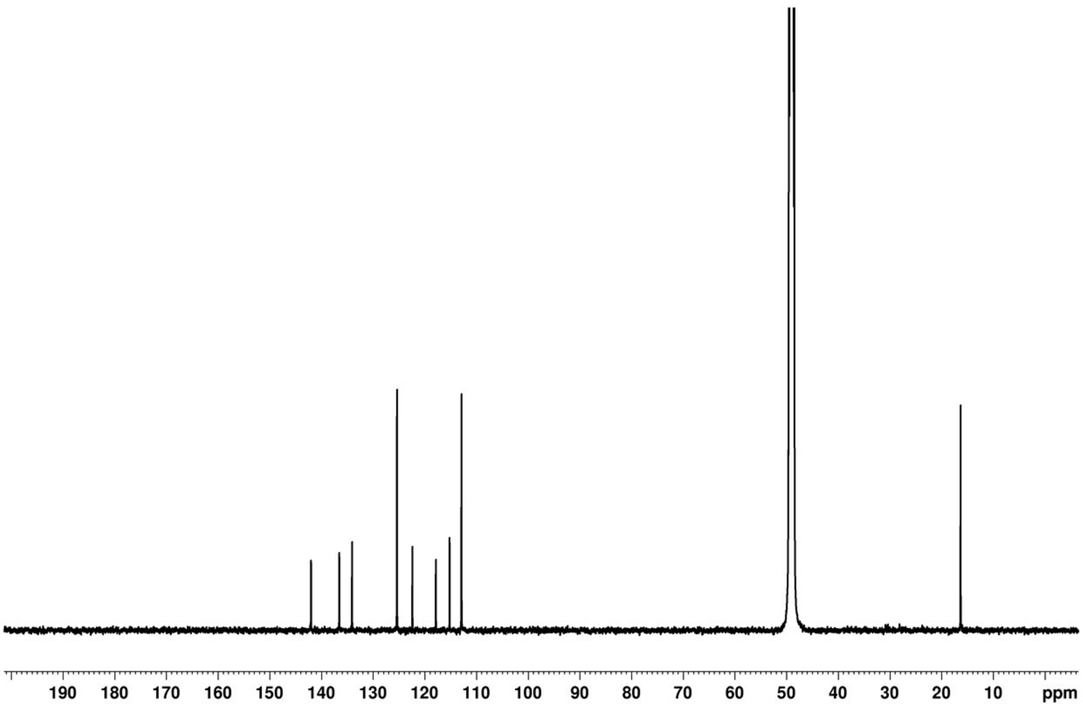

126 MHz, MeOD, ^{13}C NMR spectrum

3. Biophysical experiments

3.1 Protein expression and purification

Expression and purification of CK2α (wild-type, KKK/AAA and SF) was performed as previously described.[5]

3.2 Isothermal titration calorimetry

All ITC experiments were performed at 25 °C using a MicroCal iTC200 instrument (GE Healthcare). CK2αWT (20 mg/mL, 20 mM Tris pH 8.0, 500 mM NaCl) was diluted in Tris buffer (200 mM Tris, 300 mM NaCl, 10% DMSO) and concentrated to 20−50 µM. The compounds in 100× stock solutions were diluted into the buffer, ensuring that the DMSO concentrations were carefully matched. In a typical experiment, CK2αWT (10 µM) was loaded into the sample cell and 0.4−2.0 mM of the ligand was titrated in eighteen 2 µL injections of 2 s duration at 150 s intervals, stirring at 750 rpm. Heats of dilution were determined in identical experiments, but without protein in the cell. The data fitting was performed with a single-site binding model using the Origin software package.

3.3 X-ray crystallography

Crystallization, soaking of ligands, and structure determination were done as described before. X-ray diffraction data were collected at the Diamond Light Source and data from automated data processing with autoProc were used for the structure determination.[6] All coordinates have been deposited to the Protein Data Bank under the accession numbers in the table below.

Table S4: Data collection and refinement statistics

**Pdb ID**	7ZY0	7ZY2	7ZY5
**Ligand**	8	3	2
**Data processing:**			
Source	Diamond Beamline IO4	Diamond Beamline iO4	ESRF ID14-4
Date of datacollection	08/03/2015	11/05/2014	23/11/2013
Wavelength (Å)	0.9323	0.9180	0.9793
Spacegroup	P 1 21 1	P 1 21 1	C 2 2 21
Unit cell (a, b, c [Å],	58.679 45.828 63.283	58.782 45.916 63.286	64.740 68.680 332.880
α,β,γ [°])	90.00 111.58 90.00	90.00 111.88 90.00	90.00 90.00 90.00
Resolution limits [Å]	58.85-1.44 (1.58-1.44)	58.73-1.51 (1.59-1.51)	47.55-1.82 (1.88-1.82)
Number of molecules in ASU			
No of total/unique reflections	187051 / 56651	243427 / 48182	233850 / 63451
Multiplicity	3.3 (3.2)	5.1 (5.2)	3.7 (3.5)
Rmerge	0.043 (0.375)	0.061 (0.671)	0.051 (0.681)
Rmeas	0.051 (0.451)	0.069 (0.748)	0.059 (0.790)
I/σI	14.0 (2.8)	15.4 (2.4)	16.9 (1.8)
CC1/2	0.999 (0.838)	0.997 (0.781)	0.999 (0.677)
Completeness [%]	99.6 (99.9)	97.2 (96.1)	94.7 (90.9)
**Refinement:**			
Rwork/Rfree [%]	0.1992	0.1878 / 0.2104	0.1989 / 0.2191
Number of unique/free reflections used	56595 / 2865	47870 / 2415	63359 / 3207
R.m.s deviations:			
bond lengths [Å]	0.010	0.010	0.010
bond angles [°]	0.96	0.95	0.92
Ramachandran analysis:			
Most favoured	318 (97.8%)	316 (97.2%)	636 (98.1%)
Allowed	7 (2.2%)	8 (2.5%)	12 (1.9%)
Outliers	0 (0.0%)	1 (0.3%)	0 (0.0%)
Number of atoms:			
Protein	2769	2862	5550
Solvent atoms	160	242	346
Ligand atoms	219	281	417
Mean/Wilson B-factor	22.94 / 18.64	24.86 / 20.38	40.86 / 27.90

7ZY8	7ZYD	7ZYK	7ZYO	7ZYR
4	6	9	19	20
Diamond Beamline i24	Diamond beamline IO4-1	Diamond beamline IO2	in house	in house
17/04/2014	25/04/2015	07/10/2015	13/03/2020	20/08/2016
0.96861	0.9173Å	0.97949	1.54	1.54
C 2 2 21	P 1 21 1	P 1 21 1	P 1 21 1	P 1 21 1
64.952 67.966 333.866	58.720 45.895 63.501	58.343 45.842 63.266	58.576 45.502 63.283	58.721 45.756 63.204
90.00 90.00 90.00	90.00 111.66 90.00	90.00 111.38 90.00	90.00 111.51 90.00	90.00 111.68 90.00
166.93-1.85 (1.95-1.85)	166.93-1.85 (1.95-1.85)	54.328-1.305 (1.310-1.305)	Inf-1.58 (1.68-1.58)	Inf-1.85 (1.95-1.85)
381744 / 63759	381744 / 63759	335253 / 65260	37562	26904
6.0 (4.8)	6.0 (4.8)	5.1 (3.6)	5.41 (2.54)	4.16 (2.89)
0.109 (0.630)	0.109 (0.630)	0.063 (0.631)	0.0745 (0.4812)	0.1069 (0.4441)
0.120 (0.708)	0.120 (0.708)	0.070 (0.741)	0.549 (0.3784)	0.1020 (0.4788)
13.9 (2.8)	13.9 (2.8)	15.1 (2.6)	14.95 (2.28)	9.77 (2.09)
0.997 (0.779)	0.997 (0.779)	0.996 (0.648)	-	-
99.8 (99.7)	99.8 (99.7)	86.1 (36.5)	87.8 (79.8)	100.0 (100.0)
0.1963 / 0.2189	0.1979 / 0.2139	0.1903 / 0.2064	0.1750 / 0.2040	0.1674/ 0.2094
63564 / 3227	61388 / 3092	64749 / 3265	37538 / 1886	26875/ 1355
0.010	0.019	0.010	0.010	0.010
0.93	1.42	0.99	0.94	0.93
632 (97.7%)	318 (97.8%)	317 (97.5%)	318 (97.8%)	317 (97.5%)
14 (2.2%)	6 (1.8%)	8 (2.5%)	6 (1.8%)	8 (2.5%)
1 (0.2%)	1 (0.3%)	0 (0.0%)	1 (0.3%)	0 (0.0%)
5545	2797	2847	2820	2825
308	159	209	283	265
355	209	256	354	328
40.83 / 30.91	26.12 / 20.43	22.36 / 16.27	18.81	16.49/ 14.64

8AE7	8AEC	8AEK	8AEM
16	17	14	13
Diamond Beamline IO4	Diamond Beamline IO4	In house	In house
16/09/2016	16/09/2016	21/09/2015	20/03/2022
0.9184	0.9184	1.54	1.54
P 1 21 1	P 1 21 1	P 1 21 1	P 1 21 1
58.553 45.896 63.471	58.578 45.706 63.503	58.460 45.858 63.117	58.720 45.886 63.339
90.00 111.52 90.00	90.00 111.41 90.00	90.00 111.65 90.00	90.00 111.63 90.00
59.02-1.28 (1.31-1.28)	54.54-1.09 (1.12-1.09)	Inf - 1.65 (1.75-1.65)	Inf - 1.60 (1.70 - 1.60)
517476 / 80979	468202 / 130148	37717	41472
6.4 (6.2)	3.6 (3.5)	9.64 (3.38)	5.66 (2.94)
0.074 (1.422)	0.075 (0.739)	0.0613 (0.3099)	0.0682 (0.3348)
0.081 (1.551)	0.088 (0.875)	0.0348 (0.3218)	0.0508 (0.4492)
10.3 (1.1)	7.0 (1.5)	23.0 (3.36)	13.58 (2.26)
0.999 (0.492)	0.995 (0.591)	-	-
100.0 (100.0)	99.9 (99.9)	99.5 (97.1)	99.0(100.0)
0.2040/ 0.2317	0.1992 / 0.2139	0.1717	0.1886 / 0.2125
80700/ 4039	129904/ 6577	37615	41380 / 2101
0.010	0.010	0.010	0.017
0.98	1.01	0.94	1.33
316 (97.2%)	319 (98.2%)	319 (98.2%)	319 (98.2%)
8 (2.5%)	5 (1.5%)	5 (1.5%)	5 (1.5%)
1 (0.3%)	1 (0.3%)	1 (0.3%)	1 (0.3%)
2832	2873	2797	2831
342	395	261	199
388	453	320	267
20.20/ 12.98	17.11/ 8.91	17.98	23.02 / 18.75

3.4 NMR competition experiment

In a typical competition experiment, a stock solution of 50−500 µM competing ligand, 2.5% DMSO-d_6, 7 µM CK2α WT in 0.2 mM Tris, 100 mM NaCl, pH 8.0 was prepared. This was again split into two samples. One sample contained 5% DMSO-d6 and 50−500 µM competing ligand with a total volume of 180 µL. The second sample contained 5% DMSO-d_6, 500 µM 1 and 50−500 µM competing ligand. NMR data were recorded at 300 K with a Bruker AVANCE spectrometer operating at 500 MHz and equipped with a room temperature probe. CPMG-filtered one-dimensional (1D) 1-H experiments were run with 600 loops of length 1 ms. Water suppression was achieved with low-power presaturation and DPFGSE WATERGATE W5.[7] Saturation transfer difference (STD) experiments[8] were acquired with 2 s of alternating on- and off-resonance saturation (20 ms Gaussian pulse, +0.8 and −5 ppm), a T1ρ filter to suppress protein signals, and DPFGSE WATERGATE W5.[7] Water-LOGSY experiments were recorded using a 20 ms Gaussian to invert the water resonance, a mixing time of 1.2 s, a T2 filter to suppress protein signals, and DPFGSE WATERGATE W5.[7]

4. Cellular biology

4.1 Cell culture

All cell lines used were obtained from ATCC and were supplied as mycoplasma free. HCT116 colon carcinoma cells were maintained in McCoy's 5A (1x) + Glutamax-I growth medium (Gibco, 36600- 021) supplemented with fetal bovine serum (FBS, Gibco Life Technologies, 10270-106) at a final concentration of 10%. A549 lung adenocarcinoma cells were cultured in Dulbecco Modified Eagle medium (DMEM) (1x) +Glutamax-I (Gibco Life Technologies, 31966-021) with 10% FBS. Jurkat T-cell lymphoblastic cells were cultured in RPMI 1640 (Gibco Life Technologies, 72400-021) growth medium supplemented with penicillin-streptomycin (Sigma, P0781) and FBS with the final concentrations of each being 1% and 10% v/v respectively. All cells were grown at 37 °C/5% CO2 in a humidified environment and all the assays were performed using these culturing conditions.

4.2 Cell viability assay

Adherent cell lines (HCT116 and A549 cells) were seeded into flat-bottomed tissue culture 96-well plates in a volume of 150 µL of growth medium. HCT116 cells were seeded at 750 cells per well and A549 cells were seeded at 1000 cells per well. After 24 hours, compounds dissolved in DMSO were diluted in growth medium and were added to cells such that the final DMSO concentration was 1% (v/v) and the final volume in the well was 200 µL. Cells were then incubated in the presence compound for 72 hours before fixation. Medium was removed from cells and 100 µL of cold 1% (v/v) trichloroacetic acid was added and the plates were incubated for 30 minutes at 4 °C after which the

acid was removed and the plates were washed three times in tap water and left to dry at room temperature. The fixed cells were stained in a 0.057% sulforhodamine B/1% acetic acid solution (w/v) and incubated at room temperature with agitation for 30 minutes after which the dye was removed and the plates washed in 1% (v/v) acetic acid and left to dry. The dye was then solubilised in 10 mM Tris solution (pH8) and incubated for 30 minutes under agitation. The plates were then read on a PHERAstar plus plate reader (BMG Labtech) using the fluorescence intensity module (540-590 nm). Growth inhibition was calculated relative to DMSO controls and GI_{50} values were calculated using Graphpad Prism. Jurkat cells were seeded (20,000 cells per well) in growth medium in a 96-well flat-bottomed plate and immediately dosed with compounds (dissolved in DMSO) such that the final volume in the well was 200 µL and 1% DMSO (v/v). Cells were incubated for a further 72 hours. After this time, 5% (v/v) of CellTitre-Blue reagent (Promega) was added to each well and incubated for a further 2 hours under normal tissue culture condition, described above. The fluorescence was then measured using the PHERAstar plus plate reader (BMG Labtech) using the fluorescence intensity module (540-590 nm). Growth inhibition was calculated relative to DMSO control and GI_{50} values were calculated using Graphpad Prism.

4.3 Western Blotting

HCT116 cells (2 mL) were seeded into 6-well tissue culture plates at a seeding density of $3x10^5$ cells/mL and cultured for 24 hours prior to the addition of compound. Compound was diluted in culture medium to the desired concentration and a final DMSO concentration of 1% (v/v). Cells were harvested by trypsination (growth medium was collected and included in the analysis), washed in PBS and the pellet collected. Cells were lysed using a NP-40 lysis buffer (50 mM Tris pH 8, 150 mM NaCl, 1% NP-40) with the addition of Proteoblock protease inhibitor (Fermentas), and the phosphatase inhibitors #2 and #3 (Sigma Aldrich) at the recommended concentrations. The cell pellet was incubated in lysis buffer on ice for 2 hours and then centrifuged for 10 minutes at 4 °C at 13000 rpm on a bench top centrifuge for 10 minutes and the supernatant collected and stored at -80 °C. Protein levels were quantified using the Pierce BCA protein assay kit (Pierce, Thermo Fisher Scientific). A total of 30 µg of protein was loaded onto a 4-12% Bis-Tris gel (Invitrogen) and run for 1 hour at a constant 200 V. The gel was transferred onto PVDF membrane at 4 °C overnight at a constant 50 V. Transfer efficiency was confirmed by staining the membrane with Ponceau S (Sigma-Aldrich) after which it was incubated in blocking buffer (either 5 % Milk-TBS-0.1 % Tween 20 or 5 % BSA-TBS-0.1 % Tween 20) for 1 hour. Membranes were then incubated with either anti-AKT1 (phosphoS129) (Abcam, ab133458) or anti-Cdc37 (phospho S13) (Abcam, ab108360) antibodies diluted in 5% BSATBST for 24 hours at 4 °C. Anti-β actin was used as a loading control (Sigma-Aldrich, A5441) diluted in Milk-TBS-0.1% Tween 20. After washing, membranes were then incubated in HRP-labelled antirabbit antibody for 1 hour at room

temperature and then visualised using ECL (GE Healthcare). Where membranes were stripped for re-probing, membranes were immersed in Restore Western Blot stripping buffer (Thermo Scientific) for 15 minutes at room temperature.

5. Modelling

Modelling was performed using Glide. The modelling was based upon PDB: 7ZY2, the binding site was defined as a 10 Angstrom sphere around compound **3** bound in the αD site. Ligand structures were generated using Knime and results were visualised in Schrodinger.

6. References

1 N. K. Anand, S. D. Brown, Z. Tesfai and C. A. Zaharia, *WO2012037132A1*, 2012-03–22.

2 T. R. Alessi and J. W. Ellingboe, *US4895860A*, 1990-10–24.

3 A. Garofalo, S. De Lombaert, J. Schwarz, D. Andreotti, F. Sabbatini, E. Serra, S. Bernardi, M. Migliore, F. Budassi and C. Beato, *WO2021007477*, 2021-01–14.

4 J. Iegre, P. Brear, C. De Fusco, M. Yoshida, S. L. Mitchell, M. Rossmann, L. Carro, H. F. Sore, M. Hyvönen and D. R. Spring, *Chem. Sci.*, 2018, **9**, 3041–3049.

5 J. Iegre, P. Brear, D. J. Baker, Y. S. Tan, E. L. Atkinson, H. F. Sore, D. H. O' Donovan, C. S. Verma, M. Hyvönen and D. R. Spring, *Chem. Sci.*, 2019, **10**, 5056–5063.

6 C. Vonrhein, C. Flensburg, P. Keller, A. Sharff, O. Smart, W. Paciorek, T. Womack and G. Bricogne, *Acta Crystallogr. D. Biol. Crystallogr.*, 2011, **67**, 293–302.

7 K. Furihata, S. Shimotakahara and M. Tashiro, *Magn. Reson. Chem.*, 2008, **46**, 799–802.

8 A. Viegas, J. Manso, F. L. Nobrega and E. J. Cabrita, *J. Chem. Educ.*, 2011, **88**, 990–994.
